# A global molecular code for birth order and neuronal identity in *Drosophila*

**DOI:** 10.1038/s41586-026-10797-w

**Published:** 2026-07-22

**Authors:** Sebastian Cachero, Myrto Mitletton, Isabella R. Beckett, Elizabeth C. Marin, Laia Serratosa Capdevila, Marina Gkantia, Jelly H. M. Soffers, Haluk Lacin, Gregory S. X. E. Jefferis, Erika Donà

**Affiliations:** 1 Neurobiology Division, MRC Laboratory of Molecular Biology, Cambridge, UK; 2 Department of Zoology, University of Cambridge, Cambridge, UK; 3 Aelysia, Bristol, UK; 4 Division of Biological and Biomedical Systems, School of Science and Engineering, University of Missouri-Kansas City, Kansas City, MO USA; 5 Institute of Neuroscience, Consiglio Nazionale delle Ricerche (CNR), Vedano al Lambro, Italy

**Keywords:** Cellular neuroscience, Molecular neuroscience, Neural patterning, Cell type diversity, Sexual dimorphism

## Abstract

The assembly of functional neural circuits relies on the generation of diverse neural types with precise molecular identity and connectivity. Unlocking general principles of neuronal specification and wiring across the nervous system requires a systematic and high-resolution characterization of its diversity, recently enabled by advances in single-cell transcriptomics and connectomics. However, linking the molecular identity of neurons to circuit architecture remains a key challenge. Here we present a high-resolution developmental transcriptional atlas for the *Drosophila melanogaster* nerve cord, the central hub for sensory–motor circuits. With a considerable 38× aggregate coverage relative to its reference connectome^1,2^, our atlas captures extensive molecular diversity and enables robust alignment to the adult connectome. We identified three developmental principles underlying neuronal diversity in the nerve cord. First, the timing of neurogenesis shapes diversification of molecular identity: embryonic-born neurons diverge faster than larval-born neurons, as also observed in the adult connectome. Second, 17 transcription factors common to neurons from all lineages provide a global molecular identity code for birth order. Lastly, by mapping sex-specific transcriptional profiles to the connectome, we identified female-specific apoptosis and transcriptional divergence as key global drivers of sex specification. By revealing key organizational axes of molecular identity, this atlas opens avenues to dissect the molecular mechanisms underpinning the development and evolution of neural circuits.

## Abstract (web summary)

A high-resolution developmental transcriptional atlas of the *Drosophila melanogaster* nerve cord identifies a shared transcriptional code that records neuronal birth order and organizes neuronal diversity.

## Main

The brain’s ability to process information and produce adaptive behaviours relies on its cellular composition and circuit organization. Throughout development, neurons adopt specific molecular identities that define their physiological function and guide their integration into precise circuit architectures. While spatial and temporal patterning mechanisms coordinating neuronal fate are starting to be elucidated^3–5^, we cannot yet account for the vast cellular complexity of the brain. To gain a comprehensive understanding of how neurons are specified across the nervous system we need systematic, high-resolution characterization of this diversity. Over the past decade, this need has driven two types of large-scale atlasing efforts: single-cell transcriptomics to define molecular identities^6–10^, and connectomics to reveal anatomical and synaptic relationships of neurons^1,11–14^.

The dimensionality of these large datasets can be reduced by grouping individual neurons into cell types, which represent the functional units of neuronal circuits^14–16^. In vertebrates, recent mouse and human brain atlases have identified more than 5,000 and 3,000 transcriptional types, respectively^6,9^. Eventually it will be essential to integrate such molecular taxonomies with other key modalities including neuroanatomy and connectivity^15,16^. There is currently no clear path to establish a comprehensive correspondence between transcriptomic and anatomical types in vertebrates. However, the situation is more promising for the highly stereotyped and numerically smaller brain of *D. melanogaster*. In this case, the systematic analysis and annotation of the combined datasets of the central nervous system, the largest connectomes yet completed, revealed over 11,000 neuronal types across 160,000 neurons^2,14,17^. This complete description of the texture of a complex nervous system provides an exciting opportunity to better understand both the developmental and organizational logic of neural circuits. Nevertheless, comprehensive cross-identification of anatomical and molecular cell types remains challenging.

The most complete matching to date has been obtained in sensory systems: for olfactory projection neurons^18^ and in the visual system (optic lobes)^19–22^, where around 800 repeated ommatidia result in hundreds of copies for a relatively small number of cell types. Outside the optic lobes, where most cell types are represented by around 2 neurons per hemisphere, transcriptional coverage becomes a limiting factor: empirical observations and simulation suggest that a minimum of 20–30 cells is needed to identify a cell type in single-cell RNA-sequencing (scRNA-seq) datasets^15,23^. As a consequence, low-coverage atlases are inherently unsuited to resolve the full connectomic diversity, explaining why large-scale atlases outside the optic lobes^24–26^ reported ten times fewer neuronal types^2,14,15^. However, high coverage alone might not be sufficient to resolve connectomic types, as work in the olfactory and visual system has shown that transcriptional diversity peaks during development and decreases in adults^5,18,20,27^, making developmental atlases better suited to capture the diversity in the connectome.

Stimulated by these considerations, we have generated a developmental transcriptional atlas of the *D. melanogaster* ventral nerve cord (VNC), which houses the sensory–motor circuits that form the core interface between the nervous system and the body. The atlas is available for browsing at Scope (https://scope.aertslab.org/#/HundredDrills/*/welcome) and at a dedicated website (https://flyem.mrc-lmb.cam.ac.uk/VNCatlas). Its deep coverage (38× aggregate coverage) enabled high-resolution mapping of neuronal diversity as well as emergent organizational features of molecular identity; comprehensive annotation allowed most cells to be linked to the connectome. Together, these properties of the atlas enabled the identification of a global molecular temporal coordinate system: a sequence of 17 transcription factors (TFs) that define a persistent lineage-independent correlate of neuronal birth order. Comparison of male and female data identified apoptosis and transcriptional divergence as key developmental mechanisms of sex differences and also enabled precise matching with connectomic cell types. This atlas provides a resource and intellectual framework for identifying principles of circuit assembly.

## Developmental atlas of the nerve cord

**Fig. 1:**
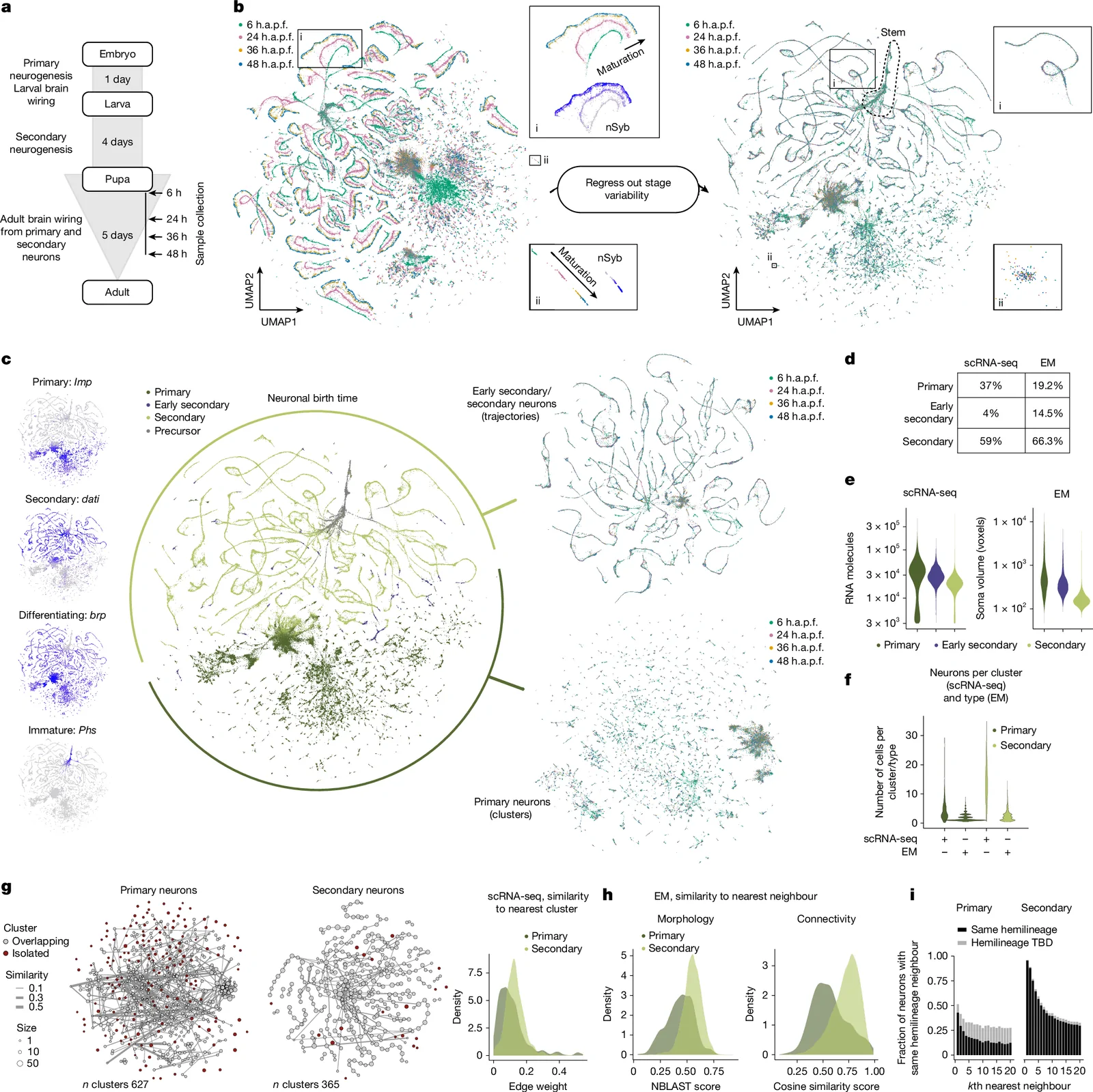
A developmental atlas of the *Drosophila* VNC identifies two modes of neuronal diversification. **a**, Two neurogenic waves in *Drosophila* development. **b**, UMAPs showing neurons coloured by stage before (left) and after (right) regression of maturation-driven variability. Insets: the same subsets of cells (i and ii) in either embedding. *nSyb* expression increases with stage. The dotted line indicates the immature neuron stem region. **c**, UMAPs for markers of neurogenic wave and maturation status (left). Middle, neurogenic wave and maturation status annotation. Right, UMAPs of primary and secondary neuronal subsets. **d**, Fractions of primary, secondary and early secondary neurons in the atlas and the connectome. EM, electron microscopy. **e**, RNA abundance in the atlas (unique molecular identifiers, left) and soma volume in the connectome (right) by neurogenesis wave. **f**, Neurons per scRNA-seq cluster after normalizing for coverage (24 + 36 + 48 h.a.p.f., 28×) and per connectomic type. **g**, Constellation plots showing intercluster overlaps. Nodes represent the cluster centroids, and the size is proportional to the cluster size. Brown indicates isolated clusters after weight filtering < 0.1. Edge widths are proportional to the summed bidirectional fraction shared between the two clusters. Given clusters A and B, the edge weight is the fraction of A’s nearest neighbours belonging to B, plus the fraction of B’s nearest neighbours belonging to A. Density plot of the strongest edge for each cluster. *n* = 627 (primary),* n* = 365 (secondary) clusters. Statistical analysis was performed using two-sided Wilcoxon rank-sum test; *P* < 1.3 × 10^−14^. **h**, Distribution of NBLAST morphology scores to the top-match neuron (left). *n* = 1,386, median = 0.45 (primary); *n* = 9,919, median = 0.55 (secondary). Right, distribution of cosine similarity connectivity scores to the nearest neighbour. *n* = 1,356, median = 0.55 (primary); *n* = 9,726, median = 0.74 (secondary). For both comparisons, secondary neurons are more similar than primary neurons. Statistical analysis was performed using two-sided Wilcoxon rank-sum test; *P* < 2.2 × 10^−16^. **i**, Fraction of neurons sharing hemilineage with the *k*th neighbour at increasing neighbour rank. Black, the same hemilineage; grey, hemilineage identity of neighbour to be determined (TBD).

**Extended Data Fig. 1:**
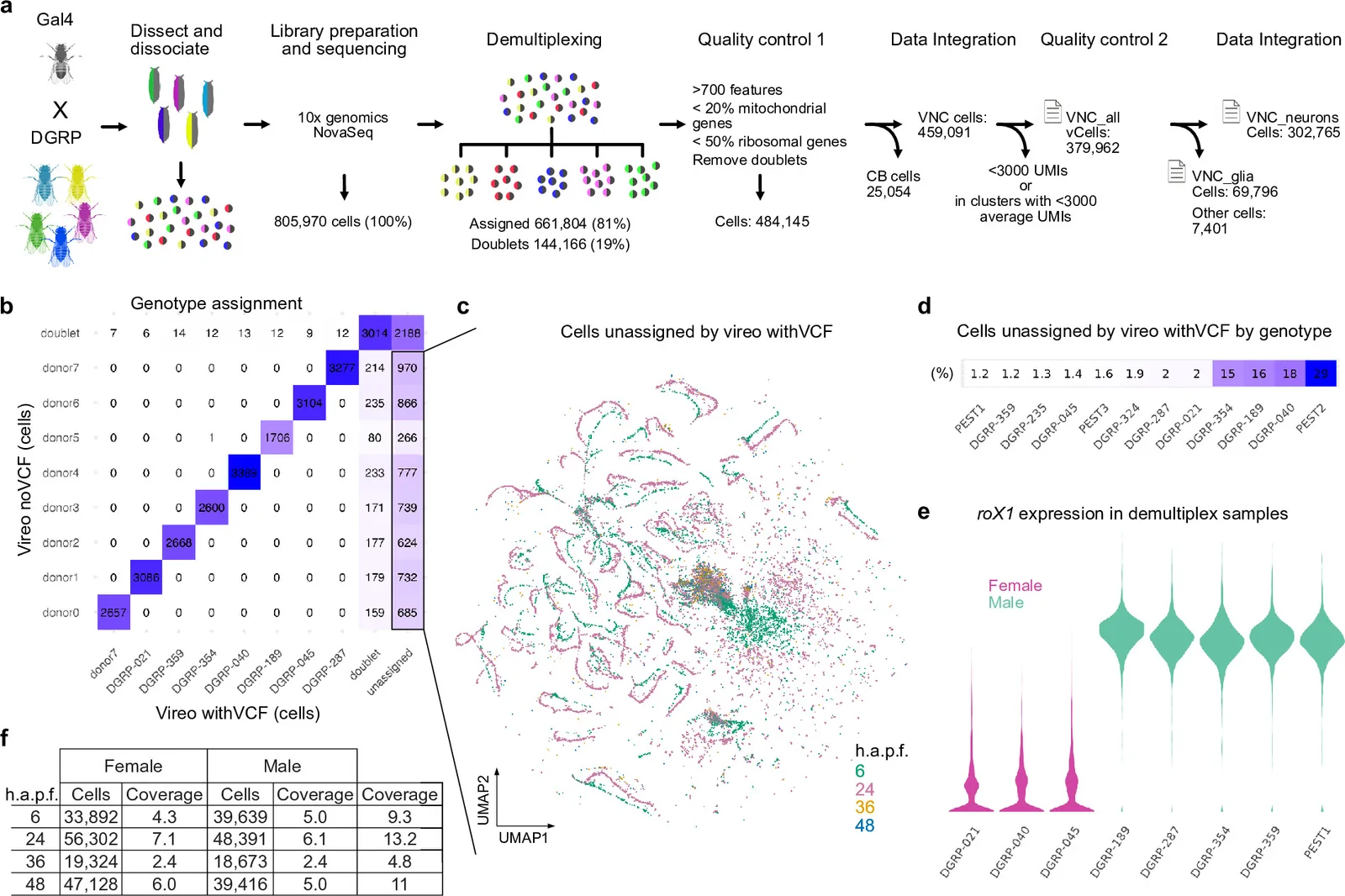
Generation of the scRNAseq dataset. **a**, Data acquisition and processing pipeline. Flies from Gal4 and DGRP lines were crossed, staged, dissected and dissociated. Cell suspensions were used to make 10X genomics libraries and sequenced. Cellranger analysis generated 805,970 cells, resulting in 661,804 singlets after demultiplexing using Vireo. 484,145 cells passed the first quality control step. After removing 25,054 cells from unrelated brain samples we merged samples from different genotypes using Seurat and removed non-neuronal cells. Following a second quality control step, 302,765 neurons were left, which form the final dataset used. **b**, Confusion matrix for one sample comparing Vireo demultiplexing results with and without a Variant Call Format (VCF) file. Box marks cells assigned to a group only in the run without the VCF, included in (c). **c**, UMAP plot showing the distribution of unassigned neurons by Vireo run with VCF, but assigned by Vireo without VCF reference across all experiments. **d**, Percentage of cells per genotype that were unassigned by Vireo, when used with a VCF, but assigned when not using a VCF reference, showing a genotype bias. **e**, Violin plots showing expression level of the long non-coding RNA roX1, a marker for male cells, in the cells from (b). Violin colour indicates the sex of the corresponding dissected animals. Correspondence between high roX1 level and male animal identity indicates the quality of the demultiplexing results. **f**, Table showing the number of neurons passing QC by stage and sex, along with coverage estimates based on the 7,880 non-glial, soma-positioned neurons per side in the male connectome (MANC).

To reveal the transcriptional diversity of the 16,000 intrinsic neurons of the VNC, we built an atlas using scRNA-seq (Extended Data Fig. 1). After quality control, we obtained 459,091 cells from four developmental stages (6, 24, 36 and 48 h after puparium formation (h.a.p.f.)), spanning the period when neuronal morphology and connectivity are established^28^ (Fig. 1a). The atlas includes a balanced number of male and female cells, making it suitable for studying the development and function of sexually dimorphic circuits (Extended Data Fig. 1f).

While the atlas contains neurons and glia (see Supplementary Fig. 1 for glia), we focused our attention on the neuronal subset. One of the main drivers of transcriptional diversity is maturation, which results in the layered organization of neurons in uniform manifold approximation and projection (UMAP) space according to the developmental stage at which they were sampled (Fig. 1b). This pattern is mirrored by graded expression of nSyb, a synaptic protein associated with neuronal maturation (Fig. 1b (i and ii)).

**Extended Data Fig. 2:**
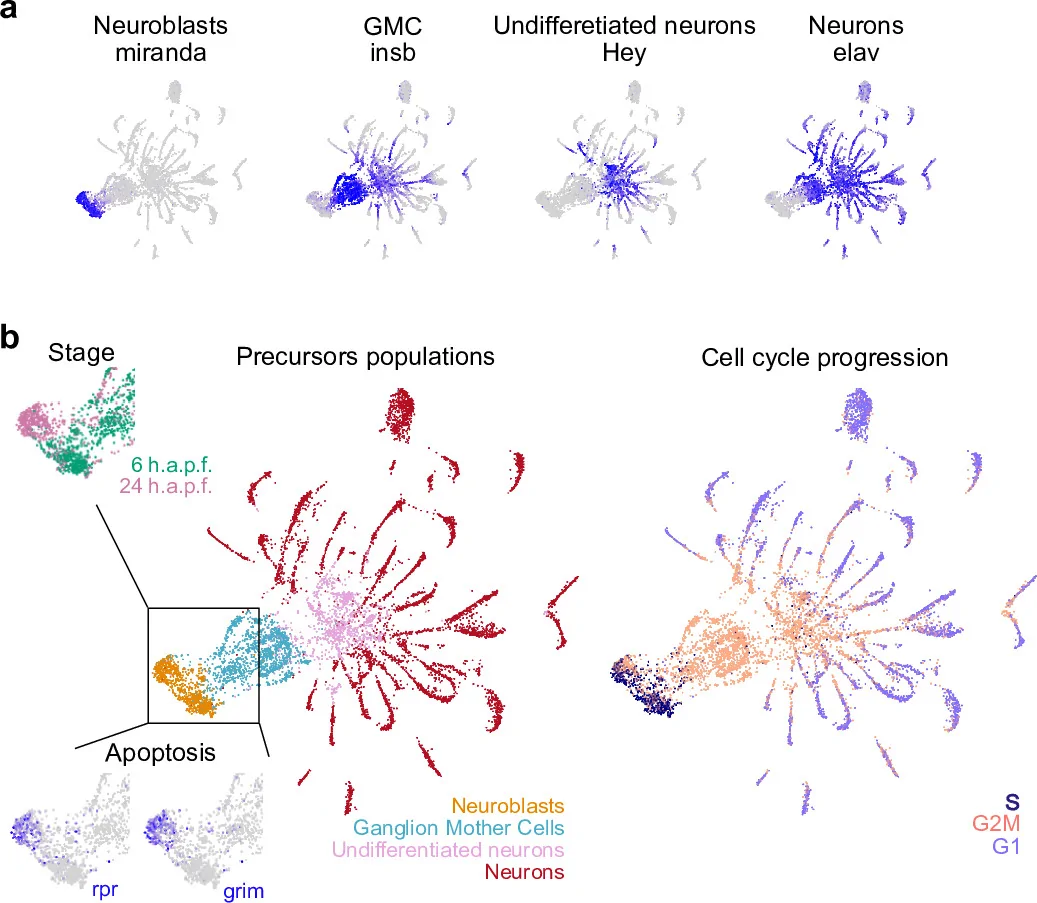
Annotation of immature cells in the stem. **a**, UMAP embedding of precursor cells from Fig. 1c showing the expression of marker genes for neuroblasts, ganglion mother cells (GMC), undifferentiated neurons and differentiating neurons. **b**, UMAP plot of precursors coloured by assigned precursor category (left) and predicted cell cycle phase (right). Insets show expression of *rpr* and *grim* in neuroblasts (bottom) according to stage.

This embedding captures the molecular dynamics of maturation but it hinders matching cell types across development (Extended Data Fig. 6c). By regressing out maturation-related variability, we integrated transcriptomes from the same cell types across different stages. One exception is the stem region, which contains neuronal precursors and less differentiated neurons from samples from the 6 h.a.p.f. and 24 h.a.p.f. that do not integrate with those from the 36 h.a.p.f. and 48 h.a.p.f. (Extended Data Fig. 2), as those stages lack the corresponding immature cells (Fig. 1b). Our neuronal atlas contains 302,765 cells representing the 7,880 neurons per lateral half of the male VNC connectome (MANC)^2^, yielding 38× aggregate coverage (per-stage coverage is shown in Extended Data Fig. 1a,f).

## Two modes of neuronal diversification

In insects, there are two waves of neurogenesis. Primary neurons are born from stem cell progenitors (neuroblasts) in the embryo where they build the larval brain and are later remodelled through pruning and regrowth of arbours during metamorphosis. The same neuroblasts produce secondary neurons during larval stages, which integrate into adult circuits only in the pupal stage^29^ (Fig. 1a). We identified primary and secondary populations in the atlas by the mutually exclusive expression of *Imp* and *dati* (Fig. 1c) and found primaries segregate into a region of punctate small groupings (clusters) and secondaries into a region of elongated low-dimensional manifolds (trajectories), typically radiating from the stem region (Extended Data Fig. 2). Outside small clusters, we found *Imp* expression at the tips of trajectories, distal to the stem. These are likely to be the first born neurons produced when the neuroblasts resume division after larval hatching and were annotated as ‘early secondaries’. Neurogenesis wave annotation is supported by the observation that secondary neurons make up 59% of the transcriptional atlas, similar to the 60% reported in a recent scRNA-seq larval atlas^30^ and to the 66% observed in MANC (Fig. 1d). Moreover, there is a similar trend in cell size, where primary > early secondary > secondary, as measured by number of RNA molecules per cell (transcriptome) and soma volume (connectome) (Fig. 1e).

Spatial segregation in UMAP space of primary and secondary neurons highlights neurogenesis wave as a strong driver of transcriptional divergence. However, local organization is also different (puncta versus trajectories) suggesting that these two populations differ in the degree of separation between one cell type and its closest neighbour, with primary neurons diverging more rapidly than secondary neurons. This distinct organization is not an artifact of the joint analysis of primary and secondary neurons, as it is preserved when the two subpopulations are analysed independently (Fig. 1c). Notably, after adjusting for coverage, many primary clusters have sizes compatible with one cell, which is the median number of cells per type in the connectome (Fig. 1f,g), while the median cluster size is three cells. By contrast, secondary neurons yielded a median of 13 cells per cluster at the same clustering resolution, compared to 2 in the connectome (Fig. 1f,g). To better compare primary and secondary neuron clusters, we quantified their degree of similarity using an overlap metric defined as the fraction of neurons of which the nearest neighbours belong to a different cluster. Using this measure, secondary neuron clusters showed greater intermixing than primary clusters, with intercluster relationships forming ordered, trajectory-like structures, in contrast to the more heterogeneous organization of primary clusters (Fig. 1g). This observation aligns with the MANC connectome, in which we find that primary neurons are statistically more distinct from their most similar neighbour than secondary ones (Fig. 1h) and that the nearest neighbour of a secondary neuron is more likely to have the same developmental origin (that is, hemilineage identity) than for primary neurons (Fig. 1i). Together, these results strongly support the idea that secondary neurons diverge more gradually than primary neurons.

## Hemilineages link the atlas to the connectome

**Fig. 2:**
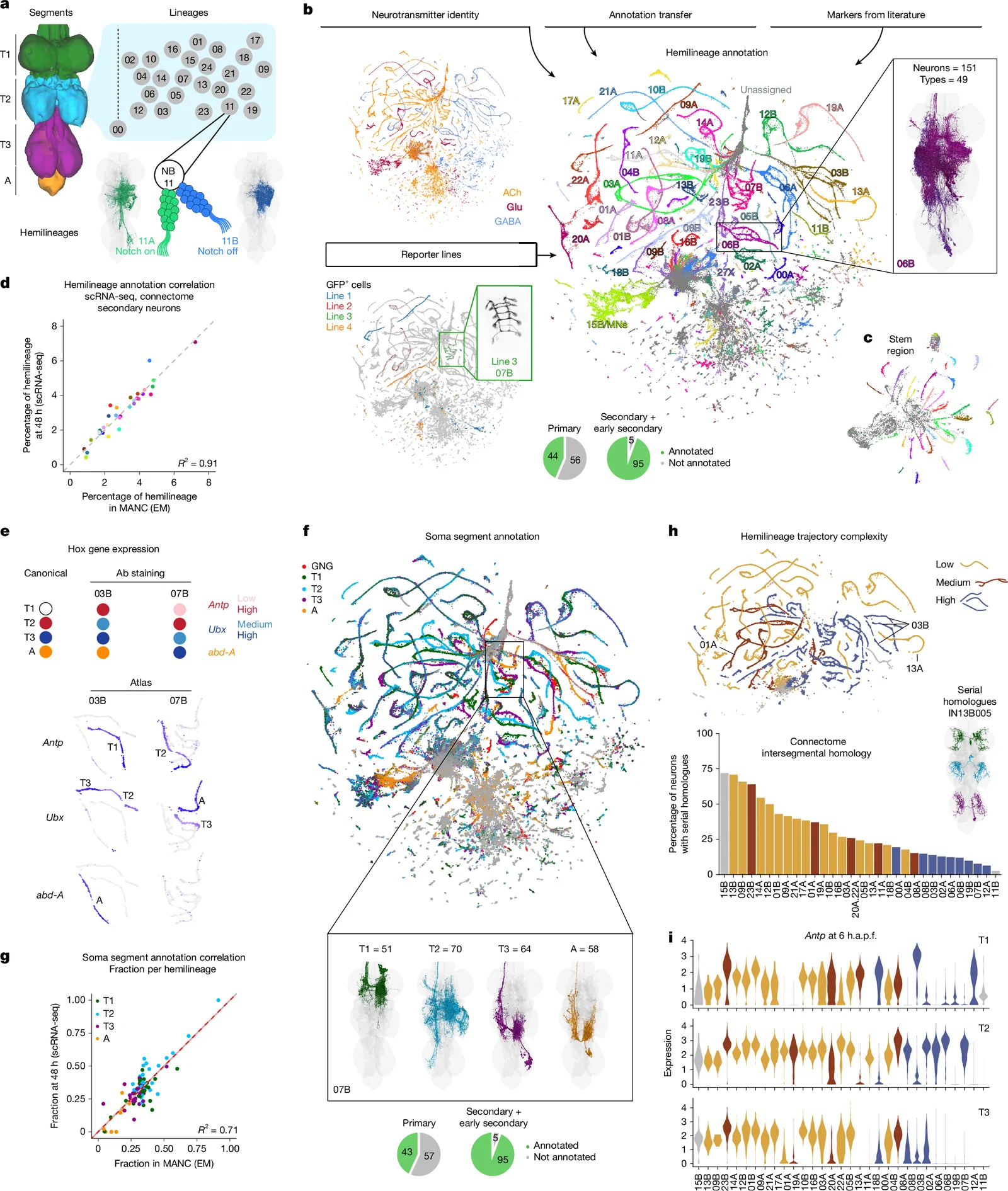
Hemilineage and segment annotations link the transcriptome to the connectome. **a**, Adult VNC showing thoracic (T1, T2, T3) and abdominal (A) segments. 25 stem cells (neuroblasts, NB) per segment generate secondary lineages, their progeny splitting into A and B hemilineages determined by Notch signalling. Male connectome (MANC) renderings for hemilineages 11A and 11B. **b**, The central UMAP shows hemilineage identity assignments based on cumulative evidence (Extended Data Fig. 4): neurotransmitter predictions, adult VNC scRNA-seq atlas^26^ annotation, markers from the literature and GFP^+^ cells from reporter lines in the atlas. Cross-matching between transcriptomic and connectomics atlases is shown for hemilineage 06B (MANC neurons in purple). The fraction of annotated neurons is shown. MNs, motor neurons. **c**, Precursor cell UMAP with manual annotation based on markers. **d**, Hemilineage annotation correlation (secondary neurons) between MANC and 48 h.a.p.f. atlas neurons. The dotted grey line indicates identity. **e**, Expression of Hox genes across segments: canonical (top left) and observed by larval stainings in hemilineages 03B and 07B (top right). Bottom, expression in 03B and 07B secondary neurons at 48 h.a.p.f. The labels show predicted segmental identities. **f**, Soma segment annotation. GNG neurons are only at 6 h.a.p.f. Inset: MANC 07B secondary/early secondary neurons (left side). The fraction of annotated neurons is shown. **g**, Segment annotation correlation showing the fraction of secondary neurons per segment per hemilineage annotated in MANC and 48 h.a.p.f. secondary neurons. The dotted grey line indicates identity. The solid red line is the fitted linear model. **h**, UMAP of secondary neurons colour-coded by hemilineage trajectory complexity (top). Bottom, the per-hemilineage proportion of secondary neurons with serial homologues in MANC coloured by trajectory complexity. Trajectory complexity in the atlas is significantly correlated with intersegmental homology in the connectome. Statistical analysis was performed using the one-sided Jonckheere–Terpstra test; *P* = 2 × 10^−4^. A neuronal type with serial homologues across thoracic segments is shown as an example. **i**, *Antp* expression at 6 h.a.p.f. per hemilineage across segments and coloured by trajectory complexity. Hemilineage order is as in **h**.

Secondary neurons are born from a defined set of larval neuroblasts (on average 25 per thoracic segment) each defining a stereotyped neuronal lineage. Sibling neurons from each lineage form two hemilineages based on Notch signalling during asymmetric cell division: A (Notch on) and B (Notch off)^28,31,32^ (Fig. 2a). Crucially, neurons of the same hemilineage bundle together to enter the neuropile, leaving a structural footprint that has been exploited to group them in the connectome achieving over 90% hemilineage annotation coverage^2^.

As hemilineage identity is one of the main sources of transcriptional heterogeneity in the VNC^26^, we examined whether the trajectories in UMAP space reflected hemilineage identity. First, we assigned neurotransmitter identity, as neurons from the same hemilineage share neurotransmitter use^2,33,34^. We found that cells in each trajectory expressed primarily just one fast-acting neurotransmitter (Fig. 2b and Supplementary Fig. 2), supporting the idea that trajectories represent hemilineages.

**Extended Data Fig. 3:**
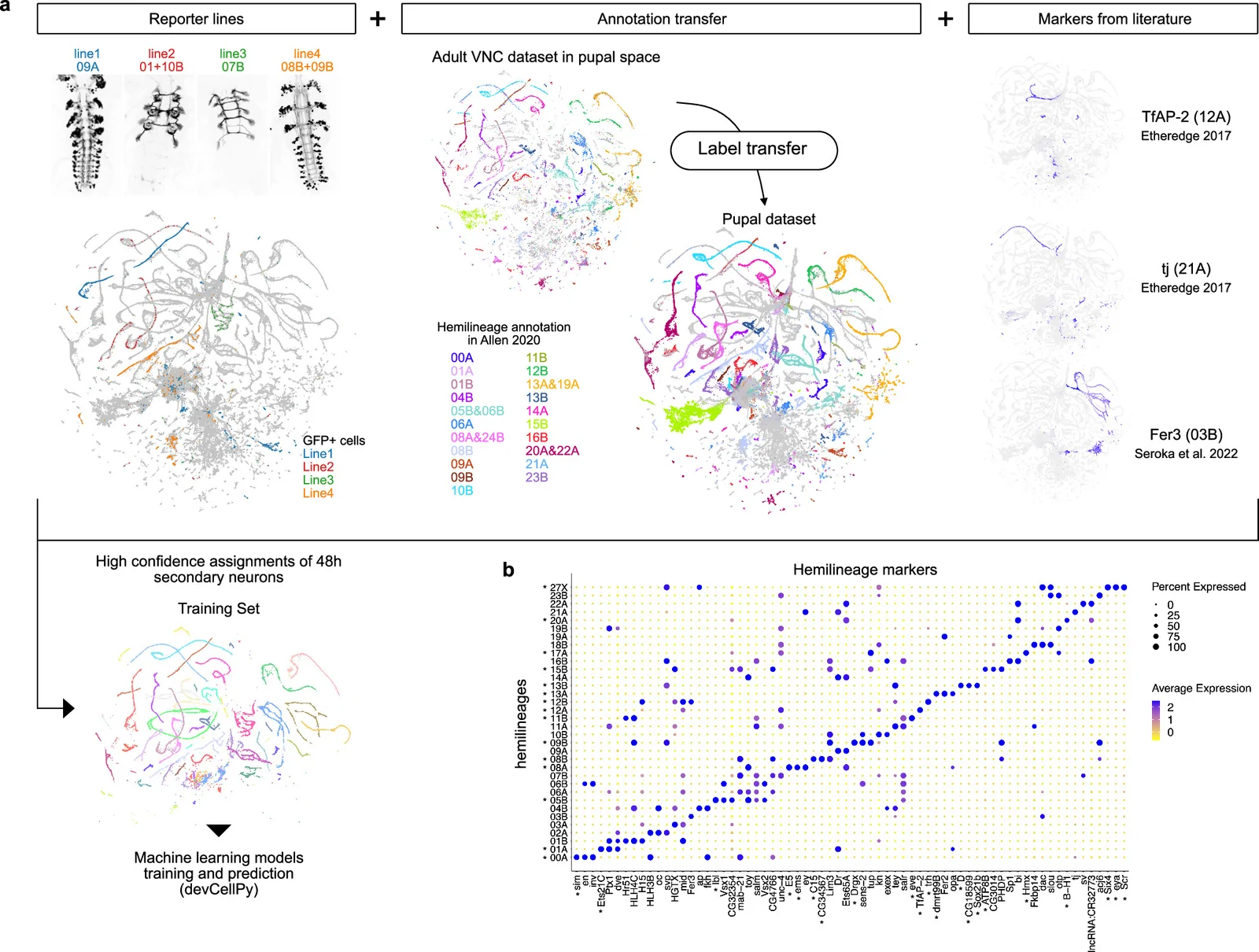
Evidence for hemilineage annotation. **a**, Cumulative evidence implemented in the pipeline to assign hemilineage identity. Left: Maximum intensity projections of confocal stacks showing GFP expression pattern of Gal4 lines used for generating the atlas. GFP positive cells coloured by the genotype of origin. Centre: adult VNC scRNAseq data from Allen et al.^26^ in the UMAP space of our atlas and the corresponding labels transferred to our atlas. Right: markers from the literature used to de-orphanize remaining hemilineages. Cumulative evidence guided preliminary hemilineage assignments used to train a machine learning classifier to predict hemilineage identity throughout the atlas. **b**, Dot plot showing expression of hemilineage-selective marker genes in secondary neurons. Top 3 positive markers expressed with a log2FC > 0.5, in more than 70% of cells in the hemilineage and less than 20% of cells in the rest of the dataset. Asterisks indicate hemilineages with unique markers and the markers themselves.

To provide direct experimental evidence for hemilineage identity, we included samples from four GFP reporter lines that label specific hemilineages (09A, 01+10B, 07B, 08B+09B)^35^ throughout development and into adulthood; the distribution of GFP-positive cells along specific trajectories confirms that trajectories correspond to hemilineages (Fig. 2b). We next used multiple sources of evidence (Fig 2b and Extended Data Fig. 3a) to annotate hemilineage identity for over 95% of secondary and 44% of primary neurons, linking them to groups of, on average, 153 neurons and 60 neuronal types in the connectome (for example, 151 neurons and 49 types in 06B). We next extracted markers for hemilineage identity, of which 88% were TFs (Extended Data Fig. 3b), and Notch status (Supplementary Fig. 3). We found that 15 out of 34 hemilineages can be distinguished by the expression of a single marker, while the rest are identified by marker combinations; these markers provide genetic access to hemilineages and helped extend hemilineage annotation to neurons in the stem region, poorly predicted by our annotation pipeline (Fig. 2c).

The accuracy of our atlas annotation is supported by a very strong correlation between the observed sizes of hemilineages in the atlas and the connectome (*R*^2^ = 0.9; Fig. 2d).

## Segmental identity links the atlas to the connectome

**Extended Data Fig. 4:**
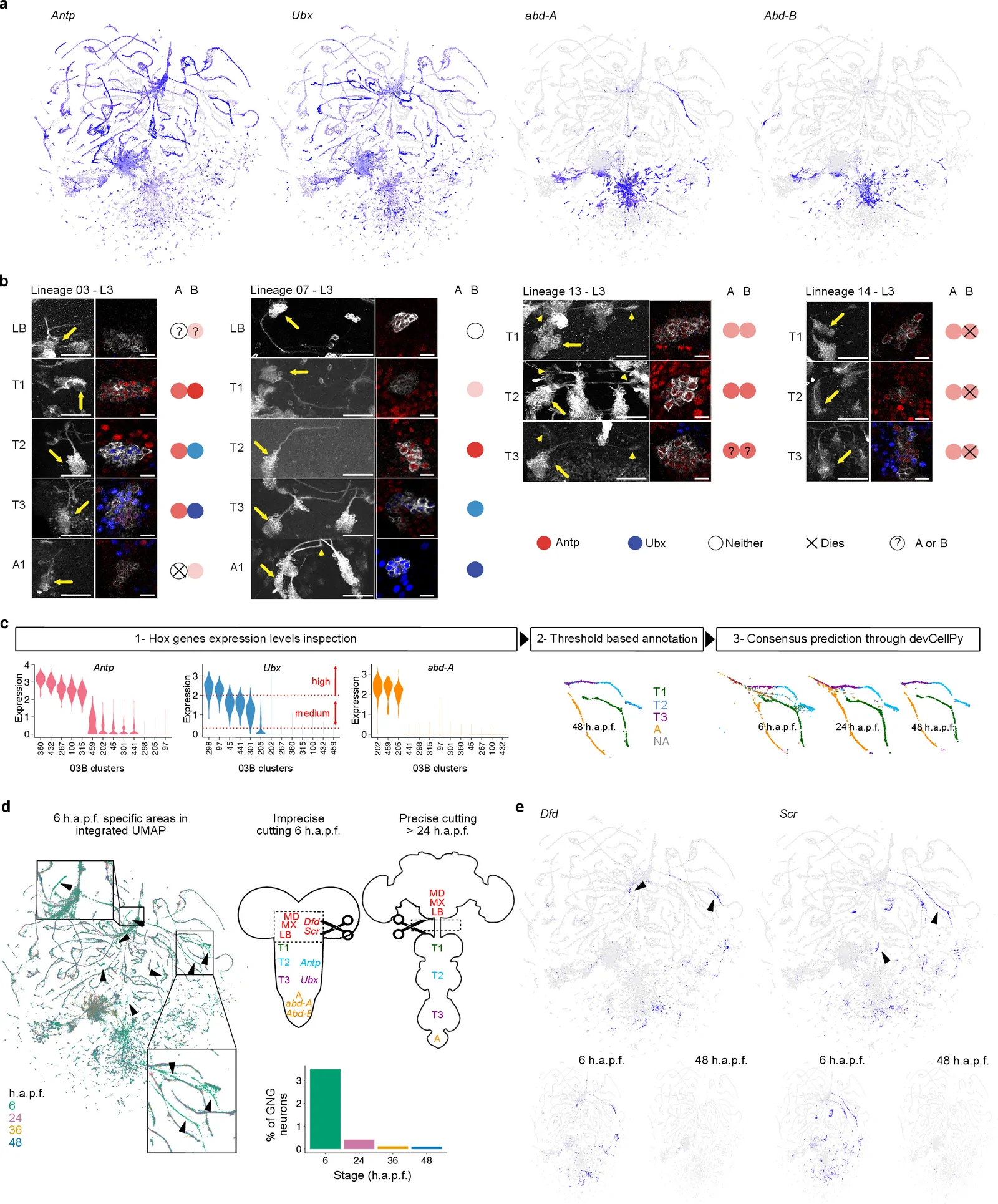
Expression of Hox genes. **a**, UMAP plots showing Hox genes expression across the dataset. **b**, Double staining for Antp and Ubx showing their protein levels across segments in specific lineages labelled by MARCM clones in late L3 larvae. Yellow arrows point to the somas of the lineages under consideration that can be identified by the location of the primary neurite. Images show partial max intensity projections. Images are representative of at least 3 stained individuals across 2 or more independent experiments. Scale bars: 40 µm grayscale images, 10 µm insets. **c**, Pipeline to annotate soma segments. Left: violin plots showing per cluster expression in 03B secondary neurons at 48 h.a.p.f. In 03B Ubx has a bimodal expression. Centre: annotation based on Hox genes thresholds and used to train devCellPy classifiers. Right: devCellPy model was used to predict per hemilineage soma segment identity across stages. **d**, Left: UMAP plot coloured by to developmental stage. Insets and arrowheads highlight 6 h.a.p.f.-specific locations outside the stem region. Top right: CNS silhouette at 6 and 24–48 h.a.p.f. The established model of the correspondence between segment and Hox gene expression is indicated. Dashed rectangles and scissors indicate areas of cutting. GNG = gnathal ganglia, LB = labial, MX = maxillary, MD = mandibular. Bottom right: bar plot with the percentage of GNG neurons per stage. **e**, UMAP plots showing expression of Hox genes specific for the GNG (*Dfd*, *Scr*) in all developmental stages (above) and split by stage (below) to highlight expression at 6 h.a.p.f., but not at 48 h.a.p.f.

Like the vertebrate spinal cord, the VNC has a repeated segmental organization: homologous lineages are generated across segments, acquiring segment-specific characteristics through anteroposterior patterning specified by gradients of Hox gene expression^36,37^ (Fig. 2a). To assess the impact of segmental organization on the transcriptome while refining our transcriptome–connectome matching, we combined Hox gene expression in the atlas with Hox protein expression in larvae to annotate segments across hemilineages (Fig. 2e and Extended Data Fig. 4a–c). In total, we provide segment identity predictions for 94% of secondary neurons and 43% of primary neurons (Fig. 2f; annotation validation is shown in Supplementary Fig. 4).

The intersection of hemilineage and segment predictions subsets the atlas into 122 groups of secondary neurons, each mapping onto a mean of 51 neurons or 26 types in the connectome. The quality of the matching is supported by a strong correlation between the fraction assigned to each group in either dataset (*R*^2^ = 0.71; Fig. 2g).

**Extended Data Fig. 5:**
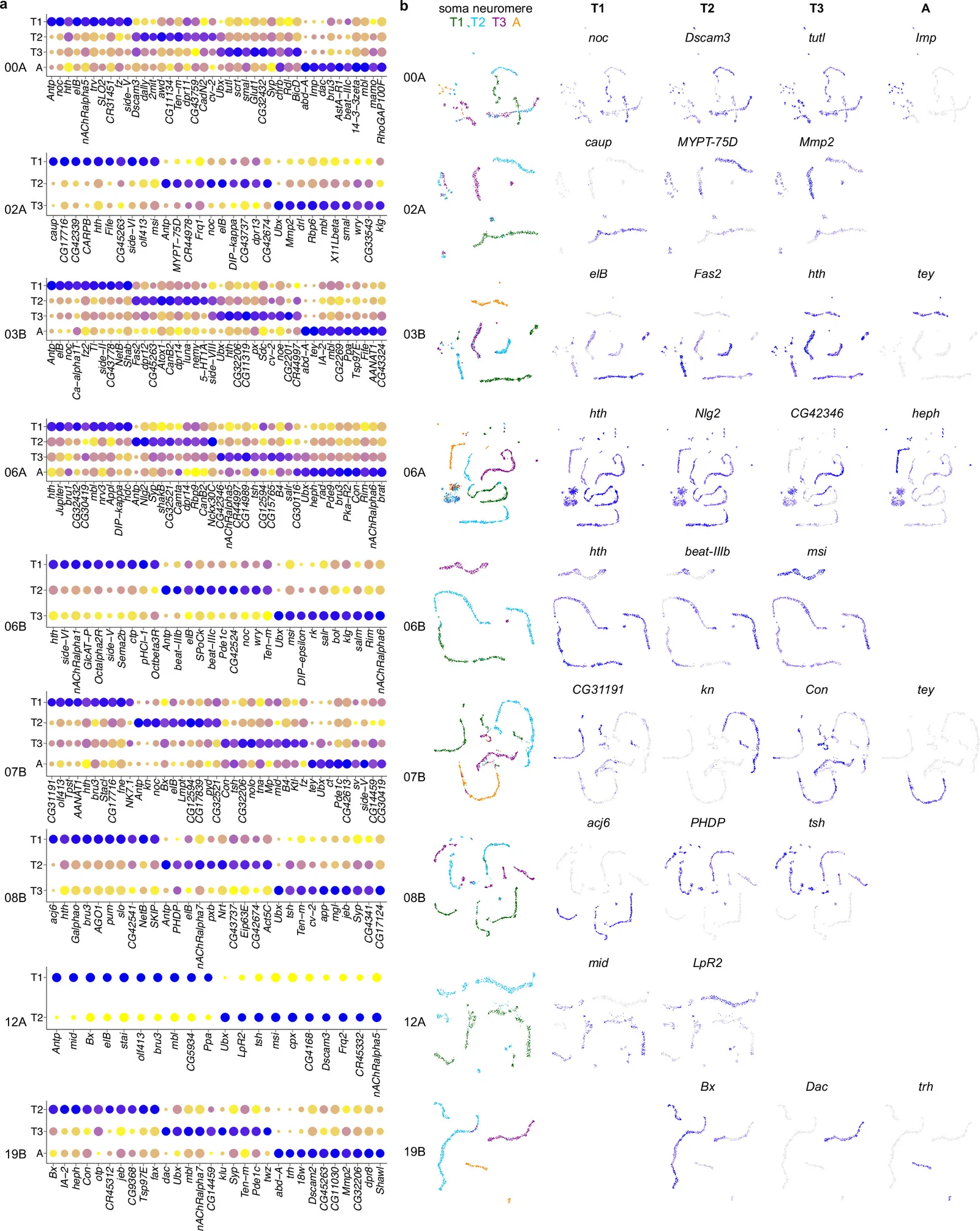
Segment specific markers in complex hemilineages. **a**, Dot plots showing the expression of the top 10 segment-specific markers for hemilineages with complex UMAP trajectories. See Supplementary Fig. 4 for marker validation. **b**, Hemilineage-specific UMAP plots showing soma segment annotation of 48 h.a.p.f. secondary neurons and the most significant segment-specific marker.

For some hemilineages, intersegmental differences are large, resulting in independent trajectories each displaying specific marker genes (such as 03B), while others overlap partially (01A) or completely (13A) (Fig. 2h, Extended Data Fig. 5 and Supplementary Fig. 4). This difference in trajectory complexity could underlie the different degree of neuronal intersegmental (serial) homology observed in the connectome (for example, IN13B005 type has serially homologous neurons in all three thoracic segments; Fig. 2h). To test this, we classified hemilineages into three categories of complexity: low (fully overlapping), medium (partially overlapping) and high (non-overlapping). We found that the transcriptional categories are significantly linked to the degree of intersegmental homology reported in the connectome^2,38^ (Fig. 2h). Notably, *Antp* is expressed across the three segments in low-complexity hemilineages, instead of the expected restriction to T2 (Fig. 2i and Extended Data Fig. 4b), suggesting that Antp may contribute to reinforcing neuronal homology across thoracic segments. This might be needed to obtain neuronal identities implementing segmentally repeated sensory–motor circuits (for example, leg movement).

## A global temporal code for birth order

**Fig. 3:**
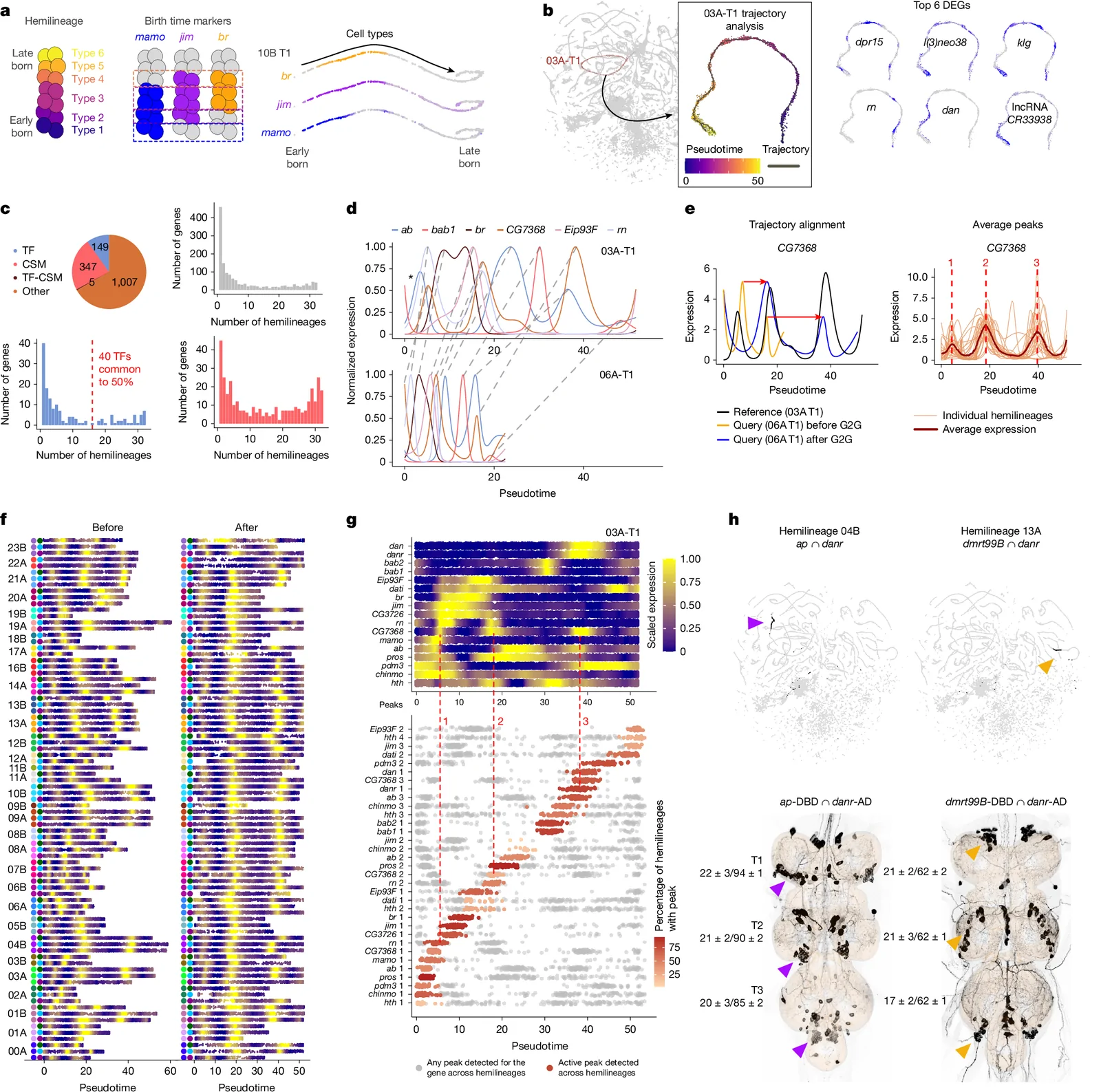
17 TFs define a global temporal code of neuronal birth order. **a**, Within hemilineages, cell types are born sequentially (left). Right, hemilineage 10B-T1 UMAPs showing three consecutive birth-order markers. **b**, Fitted trajectory and pseudotimes for 03A-T1 and 6 DEGs. **c**, DEGs with *q* < 0.01 and Moran’s value > 0.3 along trajectories. The distribution of DEGs across gene categories (pie chart) and hemilineages (histograms) are shown. Each bar represents genes shared by that number of hemilineages. Grey, DEGs; blue, TFs; red, cell surface molecules (CSM); brown, dual function. **d**, Normalized expression profile along pseudotime for 6 TFs that are differentially expressed in 95% of hemilineages. Dashed lines link expression peaks. The asterisk indicates the peak that is absent in 06A-T1. **e**, Expression profiles of *CG7368* along pseudotime for 06A-T1 before and after alignment (left) and across trajectories after alignment (right). The average expression profile and average peaks are shown in brown. **f**, *CG7368* normalized expression along pseudotime in trajectories before (left) and after (right) alignment. The *y* axis dot colours represent hemilineages from Fig. 2 (left) and segments (right; green, T1; blue, T2; and purple, T3). **g**, TFs expressed in a shared temporal order across lineages (shTFs). Top, for 03A-T1, the normalized expression along pseudotime of shTFs ordered by the first peak in the consensus sequence. Bottom, aligned gene expression peaks positions arranged by consensus order. Each dot is a peak in a single trajectory. The coloured peaks match the average active peak (the intensity represents the detection frequency across trajectories) and the grey peaks represent other peaks. **h**, Specific adult neuronal populations labelled by *danr*–hemilineage-specific marker intersection. The UMAPs represent cells co-expressing hemilineage markers (*ap* for 04B and *dmrt99B* for 13A) and *danr*. Maximum-intensity projection of confocal stacks registered to the VNCIS2 template: split-GAL4 intersection patterns in adult VNCs. The numbers indicate the average hemilineage neurons labelled per side ± s.d./average MANC hemilineage neurons per side. The arrowheads indicate neurons belonging to the expected hemilineage. In agreement with atlas predictions, clusters not belonging to 04B or 13A are visible, likely primary neurons.

**Extended Data Fig. 6:**
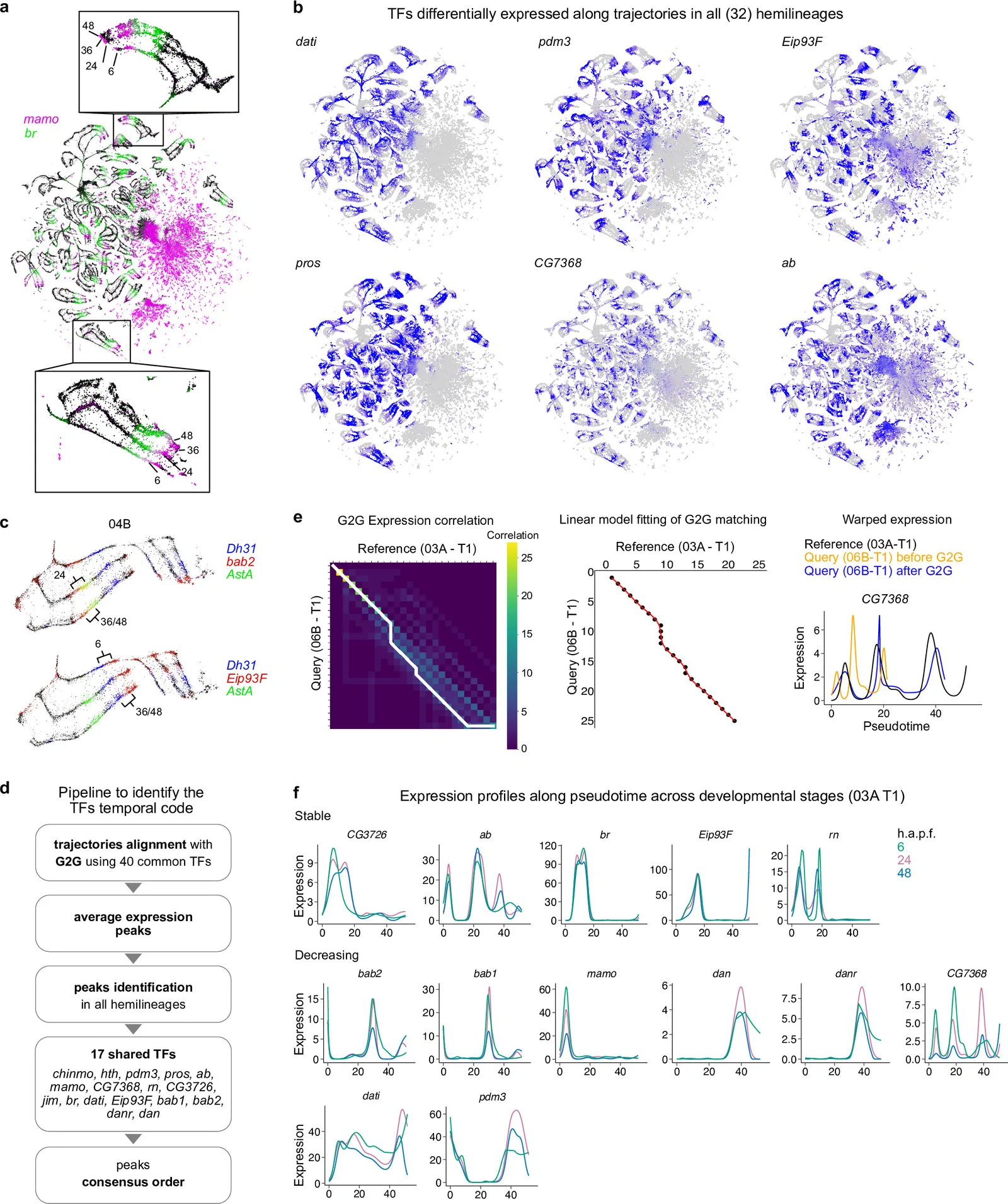
Shared TFs expression across development. **a**, UMAP plots showing expression of TFs with known birth time bias, *mamo* (magenta) and *br* (green), expressed subsequently along hemilineage trajectories across developmental stages (h.a.p.f.). Insets zoom on two specific hemilineages. **b**, UMAP plots showing striped expression along hemilineage trajectories of TFs differentially expressed in all hemilineages. **c**, UMAP plots of hemilineage 04B showing expression of a shTF (*bab2* above and *Eip93F* below, shown in red) and two neuropeptides (*Dh31* in blue and *AstA* in green) across developmental stages (h.a.p.f. are indicated), highlighting the conserved relative positioning of the shTFs to neuropeptide-specific cells across stages. **d**, Pipeline to identify TFs with shared peaks of expression across trajectories. **e**, Left: heatmap showing bin-wide expression correlation between query (06B-T1) and reference (03A-T1) trajectories, output of G2G analysis. Middle: linear model (red line) fitting of corresponding bins from G2G alignment defining the warping function to align 06B-T1 pseudotimes to 03A-T1 reference. Right: fitted *CG7368* expression profile along pseudotime for reference (03A-T1, black), query hemilineage (06B-T1) before (yellow) and after (blue) G2G based alignment. **f**, shTFs expression along pseudotime and across stages in 03A-T1 divided according to the stability of expression between 24 and 48 h.a.p.f. All UMAP plots are without stage variability regression.

Sequential generation of different neuronal types from shared progenitors is a core developmental feature of the insect central nervous system^39,40^ and other systems, such as the mammalian retina^41^. Notably, the expression patterns of *Imp* (confined to the tip of trajectories opposite to the stem; Fig. 1c) and of known genetic markers of birth-order identity^42,43^ (*mamo*, *jim* and *br*) (Fig. 3a and Extended Data Fig. 6a) provide strong evidence that neurons are arranged along trajectories according to their birth order. Neuronal diversity within lineages should therefore result from changes in gene expression along each trajectory (Fig. 3a). Using trajectory inference analysis, we identified a total of 1,508 genes differentially expressed along trajectory pseudotimes for all hemilineages, of which 149 were TFs (Fig. 3b,c). Notably, 40 of those TFs were common to more than 50% of the hemilineages, raising the possibility of a shared temporal program across hemilineages. This was supported by the observation that some TFs were expressed in a conserved order across lineages and invariant through developmental stages when assessed relative to bona fide cell-type-specific markers such as neuropeptides (Fig. 3d and Extended Data Fig. 6b,c).

**Extended Data Fig. 7:**
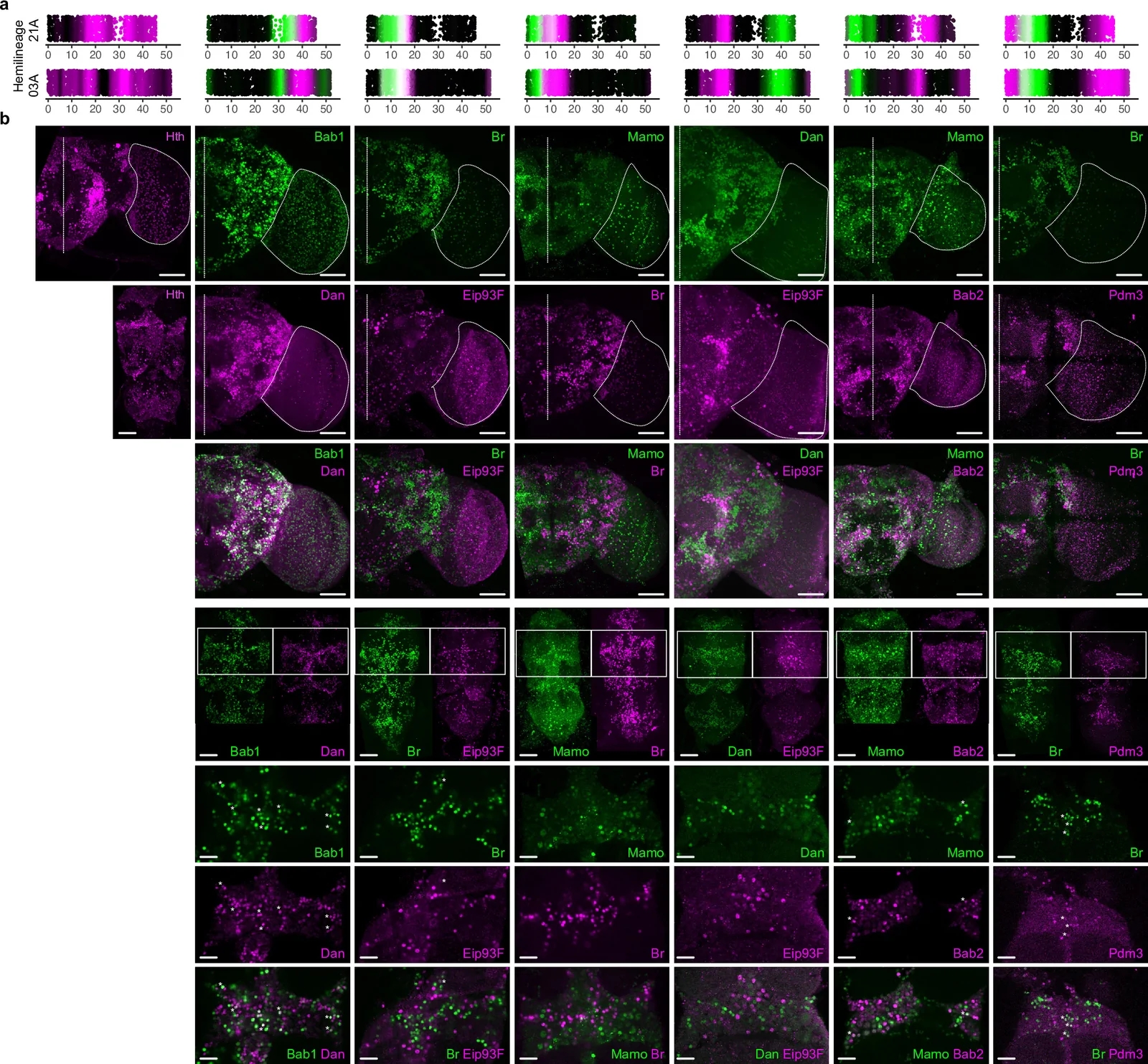
Validation of shared TFs expression. **a**, Scatter plots of normalized shTFs expression in hemilineages 21 A and 03 A along warped pseudotime. shTF and colour correspond to columns in b. **b**, Confocal stack maximum intensity projections of immunostainings for shTFs in the brain (top 3 rows) and VNC (bottom 4 rows). The brain midline is indicated with a vertical dotted line and the approximate contours of the optic lobes are highlighted with a dotted circumference in the top two rows. VNC top row: maximum intensity projections of confocal stacks. Some VNCs are missing the abdominal ganglion. White rectangles indicate the approximate areas shown in the bottom 3 rows. VNC bottom 3 rows: single slices showing individual shTFs expression (top 2 rows) and their overlap (bottom row). Examples of co-expressing cells are labelled with asterisks. Images are representative of at least 3 stained individuals across 2 or more independent experiments. Scale bars: brain and VNC 50 µm, VNC insets 20 µm.

To facilitate a direct comparison of gene expression dynamics across hemilineages and segments, we aligned all trajectories to a single reference trajectory—03A-T1, one of the longest continuous trajectories in the atlas (Fig. 3e and Extended Data Fig. 6d,e). This shared space enabled us to identify expression peaks that are aligned in pseudotime across hemilineages and segments (Fig. 3e,f). As a result, we found 17 TFs that exhibit a conserved pattern of expression along pseudotime and across hemilineages (*hth*, *chinmo*, *pdm3*, *pros*, *ab*, *mamo*, *CG7368*, *rn*, *CG3726*, *jim*, *br*, *dati*, *Eip93F*, *bab1*, *bab2*, *danr* and *dan*) (Fig. 3g and Supplementary Fig. 5), hereafter referred to as shared TFs (shTFs). Several shTFs are expressed multiple times along trajectories, expanding the number of conserved expression peaks to 33 (Fig. 3g). Most shTFs show persistent expression throughout developmental stages (Extended Data Fig. 6f) and we confirmed protein expression in adults for nine of them (Hth, Bab1, Bab2, Dan, Br, Eip93F, Mamo, Pdm3 and Pros), in both the VNC and central brain (Extended Data Fig. 7). Thus, the combinatorial action of shTFs offers a possible mechanism to generate neuronal diversity. Moreover, the intersection of shTFs expression with hemilineage-specific markers could be turned into a modular genetic approach to access cell types. We confirmed this possibility by labelling small populations of neurons born during the *danr* temporal window from hemilineages 04B and 13A in adults using the split-GAL4 system (Fig. 3h).

**Fig. 4:**
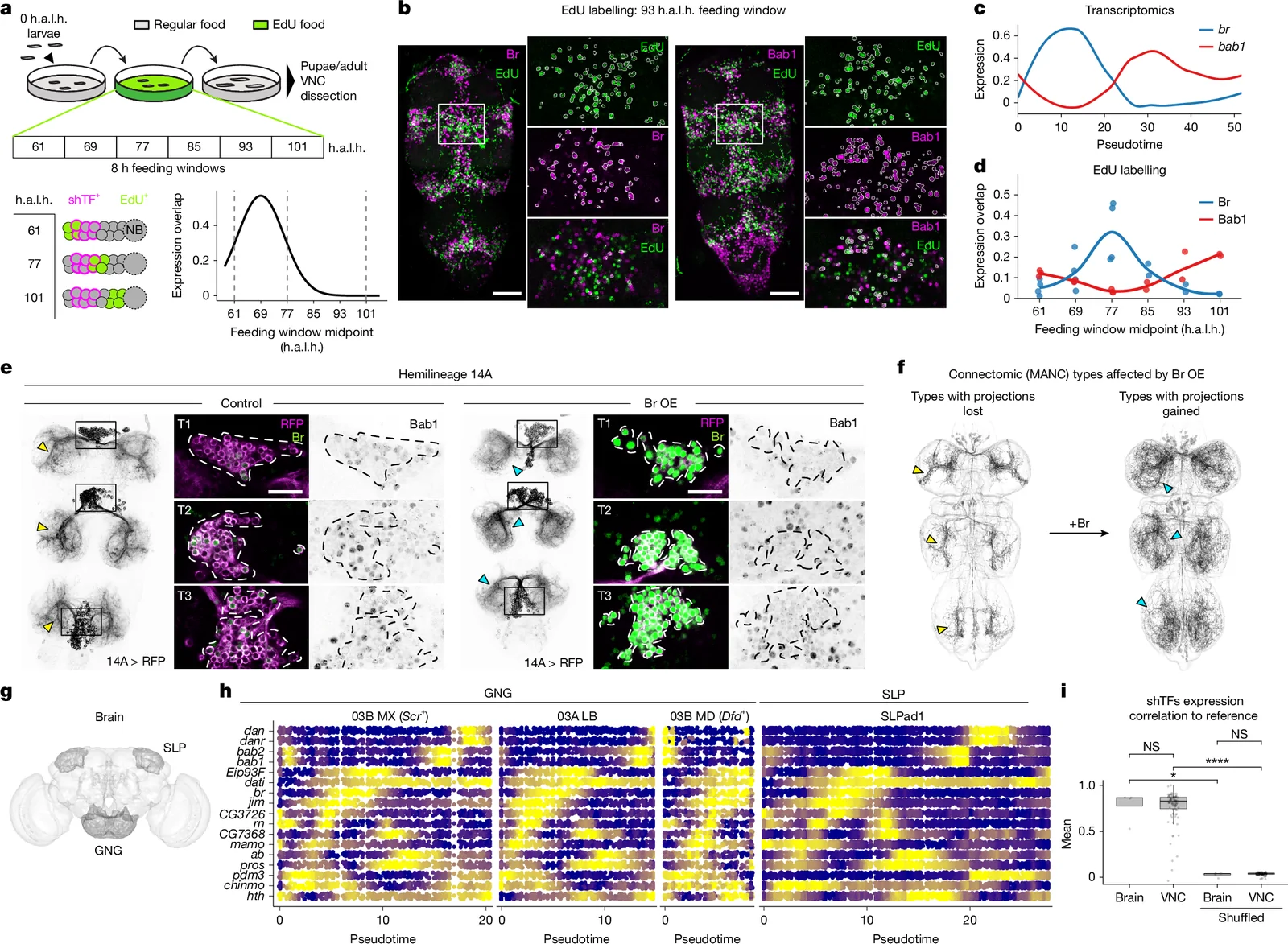
The VNC’s global temporal transcription code is conserved in the brain. **a**, EdU pulse–chase experiments. h.a.l.h., hours after larval hatching. **b**, EdU-labelled (green) VNCs from larvae fed during the 93 h.a.l.h. window, together with Br (magenta, left) or Bab1 (magenta, right). Whole VNCs are displayed as maximum-intensity projections. Insets: single *z* plane and the corresponding segmentation results for EdU (top), shTF (middle) and their intersection (bottom). Scale bars, 50 µm. **c**, Expression along pseudotime for *br* (blue) and *bab1* (red) in hemilineage 03A-T1. **d**, Fraction of shTF^+^ cells colocalizing with EdU across feeding windows (see the ‘EdU labelling’ section of the Methods). Each dot represents one VNC (*n* = 2–4 per condition). **e**, Overexpression (OE) of Br-Z4 isoform in hemilineage 14A causes the disappearance of Bab1^+^ cells within 14A and a change in projections morphology. Projections lost (yellow) or gained (cyan) in the Br-Z4-overexpression condition are marked by arrowheads. Whole VNCs are displayed as maximum-intensity projections. Insets: a single *z* plane. Scale bars, 20 µm. Images are representative of *n* = 4 per condition. **f**, Connectomic (MANC) types lost (left, yellow arrowheads) or gained (right, cyan arrowheads) by Br-Z4 overexpression. **g**, Rendering of the brain highlighting regions of origin for the hemilineages examined in **h**. **h**, Expression of shTFs for hemilineages in the brain as in Fig. 3g. GNG data from 6 h.a.p.f. and SLP data from 6–48 h.a.p.f. LB, labial; MX, maxillary; MD, mandibular. **i**, shTFs expression correlation between the reference trajectory (03A-T1) and aligned trajectories from brain (**h**) or VNC. Each dot represents the mean correlation across 17 shTFs for a hemilineage–segment combination (*n* = 4 CB, 89 VNC). The plot shows the median of the mean correlation ± interquartile range (IQR); the whiskers extend to the most extreme datapoints within 1.5 × IQR. Shuffled, correlations were calculated by shuffling shTF identity 200× per hemilineage. Statistical analysis was performed using two-sided Wilcoxon rank-sum tests; *P = 0.029, ****P < 2 × 10^−16^; NS, *P* > 0.5.

To validate that shTF expression is locked to neuronal birth time, we performed pulse–chase labelling, in which larvae were fed EdU during non-overlapping time windows (Fig. 4a). We found that neurons expressing shTFs from non-overlapping temporal domains in the atlas (*br* and *bab1*; Fig. 4b,c) are born in distinct temporal windows in the larva: Bab1^+^ cells are born in early and late windows, while Br^+^ cells are born in between (Fig. 4d), consistent with their expression in the atlas.

## Broad defines neuronal identity

The persistent expression of shTFs suggests they may act as terminal selector genes^44^. We tested this possibility by looking for changes in neuronal identity when ectopically expressing Br outside of its temporal window in hemilineage 14A. We used Bab1 expression as a readout of genetic interaction between shTFs expressed in non-overlapping temporal windows (Fig. 4c) and found that, while it is readily detected in control animals, ectopic Br expression eliminates Bab1 expression within 14A neurons (Fig. 4e). Furthermore, some neuronal processes are substantially reduced, while others are enhanced (Fig. 4e), with no significant changes in the number of neurons (mean ± s.e.m. in thoracic segment 1 (T1), overexpression, 152 ± 1.5, *n* = 4; control, 155.3 ± 2.0, *n* = 3; two-tailed Student’s *t*-test, *P* = 0.23). Notably, in the MANC connectome, we identified 14A neuronal types compatible with the lost or gained processes (Fig. 4f). We conclude that ectopic Br expression is sufficient to shift neuronal identity within the 14A hemilineage, supporting a role as a terminal selector in the VNC.

## The shTF code is conserved in the brain

We examined whether the shTF code extends to secondary neurons in the brain by analysing a small subset of hemilineages in two brain regions: the gnathal ganglia (GNG), evolutionarily linked to the VNC, and the superior lateral protocerebrum (SLP), a more derived region without clear transcriptional, morphological or functional homology to the VNC^45^ (Fig. 4g). For the GNG, we used trajectories present in 6 h.a.p.f. samples due to imprecise separation of brain and VNC tissues (Extended Data Fig. 4d,e), while the SLP hemilineage was isolated from an independent brain dataset (Methods).

In both regions and across all four hemilineages, shTFs exhibited expression patterns consistent with those observed in the VNC (Fig. 4h). We quantified this observation by calculating expression correlation between each hemilineage and the 03A-T1 reference. We did not observe a statistically significant difference between brain and VNC scores (Fig. 4i), supporting the notion that shTFs influence the development of all secondary neurons in the central nervous system, excluding the optic lobes.

## Sex differences across the nerve cord

Many aspects of sexually dimorphic behaviours are implemented by neural circuits in the VNC, such as courtship song production in male flies or post-mating behaviour in female flies^46^. VNC connectomes for both sexes enable comparative analysis and the identification of dimorphisms at scale^17^, of which the origin and nature could be elucidated by adding developmental molecular information.

**Fig. 5:**
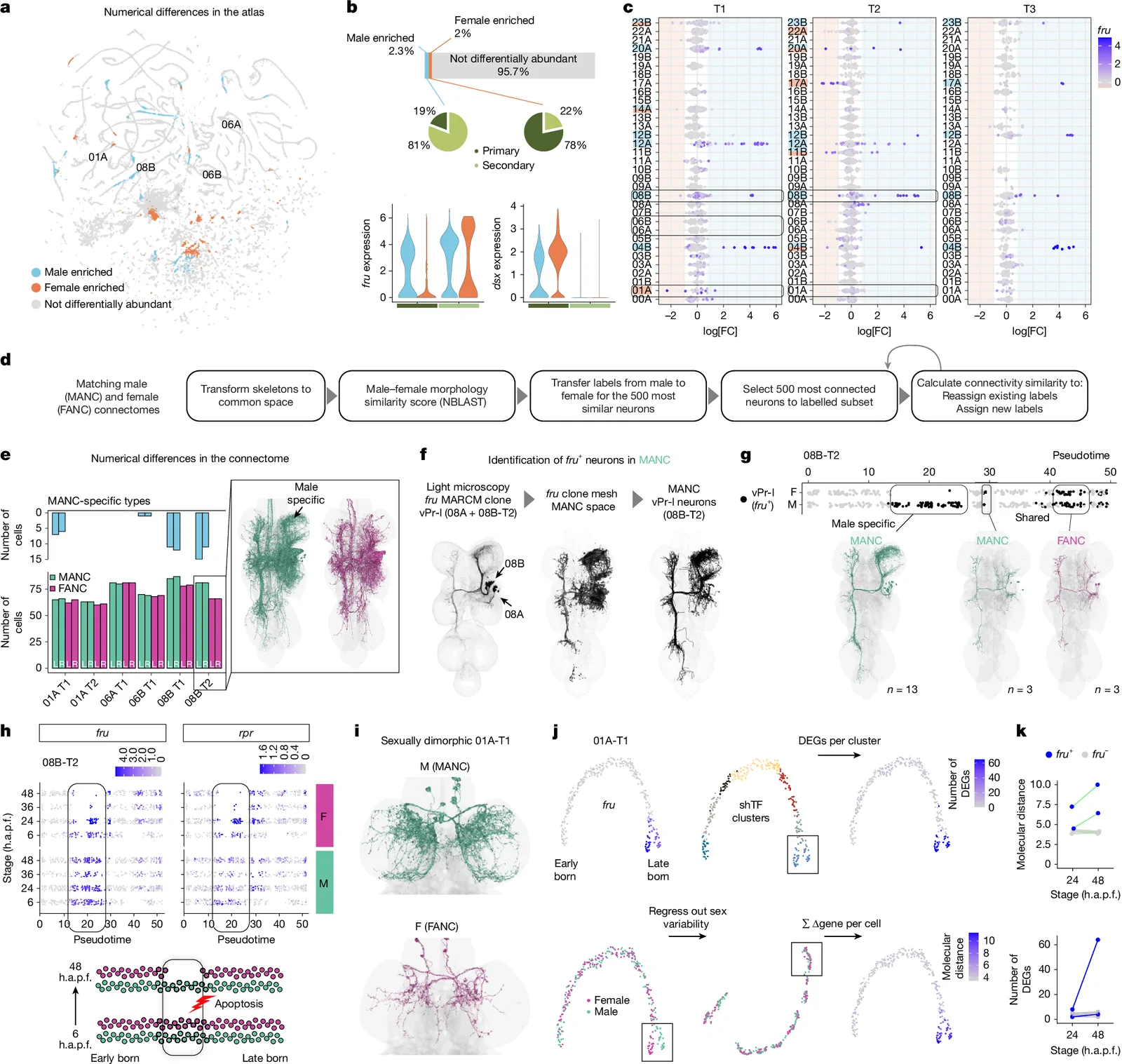
The transcriptional atlas predicts sex differences between male and female connectomes. **a**, UMAP of differentially abundant regions per sex at 48 h.a.p.f. (blue, male enriched; orange, female enriched). **b**, Percentage of neurons enriched per sex at 48 h.a.p.f.; percentage of primary and secondary neurons in sex-enriched regions; and *fru* and *dsx* expression in sex-specific neighbourhoods at 48 h. **c**, Beeswarm plots showing the secondary neuron differential abundance at 48 h.a.p.f., grouped by hemilineage and soma segment. Each dot corresponds to a neighbourhood in miloR analysis, coloured by mean *fru* expression. The boxes show hemilineages proofread in FANC. FC, fold change. **d**, Pipeline for matching the MANC and FANC connectomes. **e**, Proofread hemilineage annotation in FANC. Bottom, neurons per hemilineage, per side, per sex after proofreading (L, left; R, right). Top, the number of MANC neurons belonging to types for which FANC matches were not found by the automatic pipeline. Inset: 08B-T2 manually curated neurons in MANC (green, left) and FANC (magenta, right) in MANC space. **f**, Pipeline to identify candidate *fru*^+^ neurons in MANC starting from MARCM clones. **g**, The atlas predicts sex-specific and shared *fru*^+^ neurons in the connectome. Top, 48 h.a.p.f. secondary 08B-T2 neurons arranged by pseudotime and coloured by vPr-l clone. Bottom, vPr-l neurons in the connectome. Green, MANC; magenta, FANC. Left, male-specific neurons. Right, shared neurons. *n* values indicate the number of neurons. **h**, *fru* (left) and *rpr* (right) expression in 08B-T2 secondary neurons along pseudotime, split by sex and stage. **i**, Male and female dimorphic *fru*^+^ neurons. **j**, UMAP showing 01A-T1 secondary neurons at 48 h.a.p.f. Colours represent *fru* expression, cluster identity calculated using shTFs, the number of DEGs per cluster, sex and per-cell correction score after sex regression. **k**, Molecular differences at 24 and 48 h.a.p.f. Average correction score per cluster (top). Number of DEGs between male and female per cluster (bottom). The lines connect corresponding clusters. Green or blue lines indicate *P* < 0.01, as calculated using two-sided Student’s *t*-test with Bonferroni correction. *n* > 26.

**Extended Data Fig. 8:**
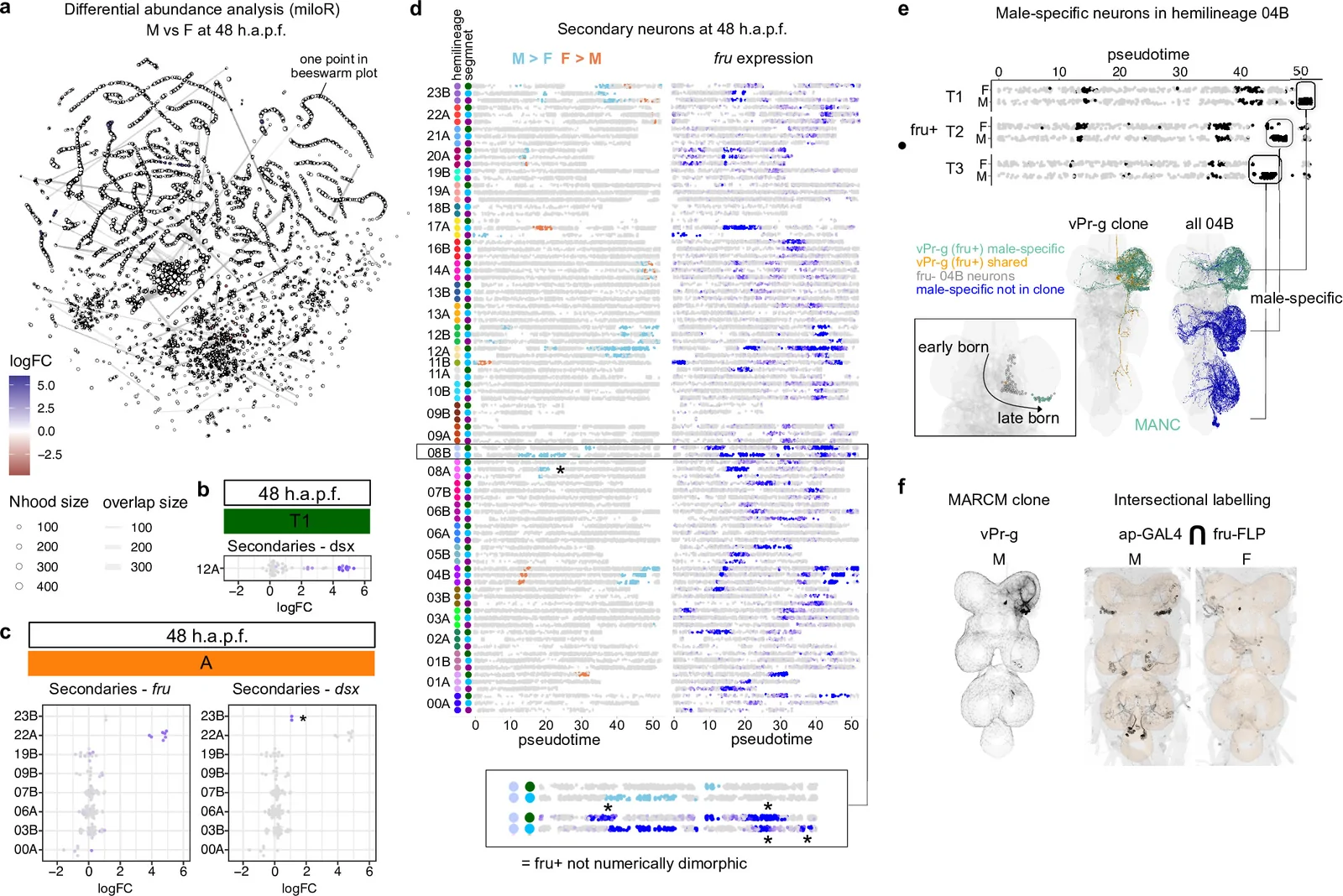
Atlas predicts sexual dimorphisms. **a**, Differentially abundant neighbourhoods at 48 h.a.p.f. resulting from miloR analysis. **b**, Beeswarm plot coloured by mean *dsx* expression, enriched in male-biased neighbourhoods in 12A-T1 at 48 h.a.p.f. **c**, Beeswarm plots of abdominal secondary hemilineages at 48 h.a.p.f. coloured by mean *fru* or *dsx* expression. **d**, Comparison between differentially abundant regions and *fru* expression. Atlas cells are distributed along pseudotime and separated according to hemilineage and segment. Inset shows 08B hemilineage in T1 and T2. Asterisks refer to fru-expressing regions that are not numerically dimorphic. Refer to Fig. 2f for segment colour correspondence. **e**, The atlas predicts fru+ male-specfic neurons in hemilineage 04B across segments. Top: dot plot showing atlas secondary 04B neurons at 48 h.a.p.f. in all segments arranged by pseudotime and coloured by *fru* expression. Bottom: vPr-g clone neurons in MANC (left). Colours indicate male specificity (green = specific, orange = shared). All male-specific 04B neurons in MANC (right). Male-specific neurons outside vPr-g clones are in blue. Inset shows soma position of 04B secondary neurons in T1 arranged according to birth order. **f**, Neurons labelled by genetic intersection of ap-GAL4 and fru-FLP in male (middle) and female (right), corresponding to 04B fru+ neurons. Max intensity projections of confocal stacks are shown. Male fru clone mapping to 04B-T1 is shown as reference (left).

The balanced number of cells per sex in the atlas enabled us to identify regions displaying differential abundance, containing 4.3% of the neurons at 48 h.a.p.f. (Fig. 5a,b and Extended Data Fig. 8a). Male-enriched cells are more likely to be secondary neurons (81%), while female-enriched cells are biassed towards primary neurons (78%) (Fig. 5b) and these differences correlated with the expression of the key sex-determination genes *fruitless* (*fru*) and/or *doublesex* (*dsx*)^46^ (Fig. 5b).

Among the well-annotated secondary neurons, sexual dimorphisms map to a small number of hemilineage–segment combinations (Fig. 5c and Extended Data Fig. 8b,c). We validated our predictions in the male (MANC) and female (FANC) connectomes, focussing on six hemilineage–segment combinations: 08B-T1 and 08B-T2 (male enriched), 01A-T1 (reported sexually dimorphic^47^) and 01A-T2, 06A-T1 and 06B-T1 (isomorphic) (Fig. 5c). We established an automated pipeline to obtain one-to-one matching between MANC and FANC neurons and transfer hemilineage labels from the well-annotated MANC to FANC (Fig. 5d and Methods). As predicted by the atlas, we found the greatest numerical differences in 08B-T1 and 08B-T2, which also had the largest number of MANC-specific neuronal types (Fig. 5e).

**Extended Data Fig. 9:**
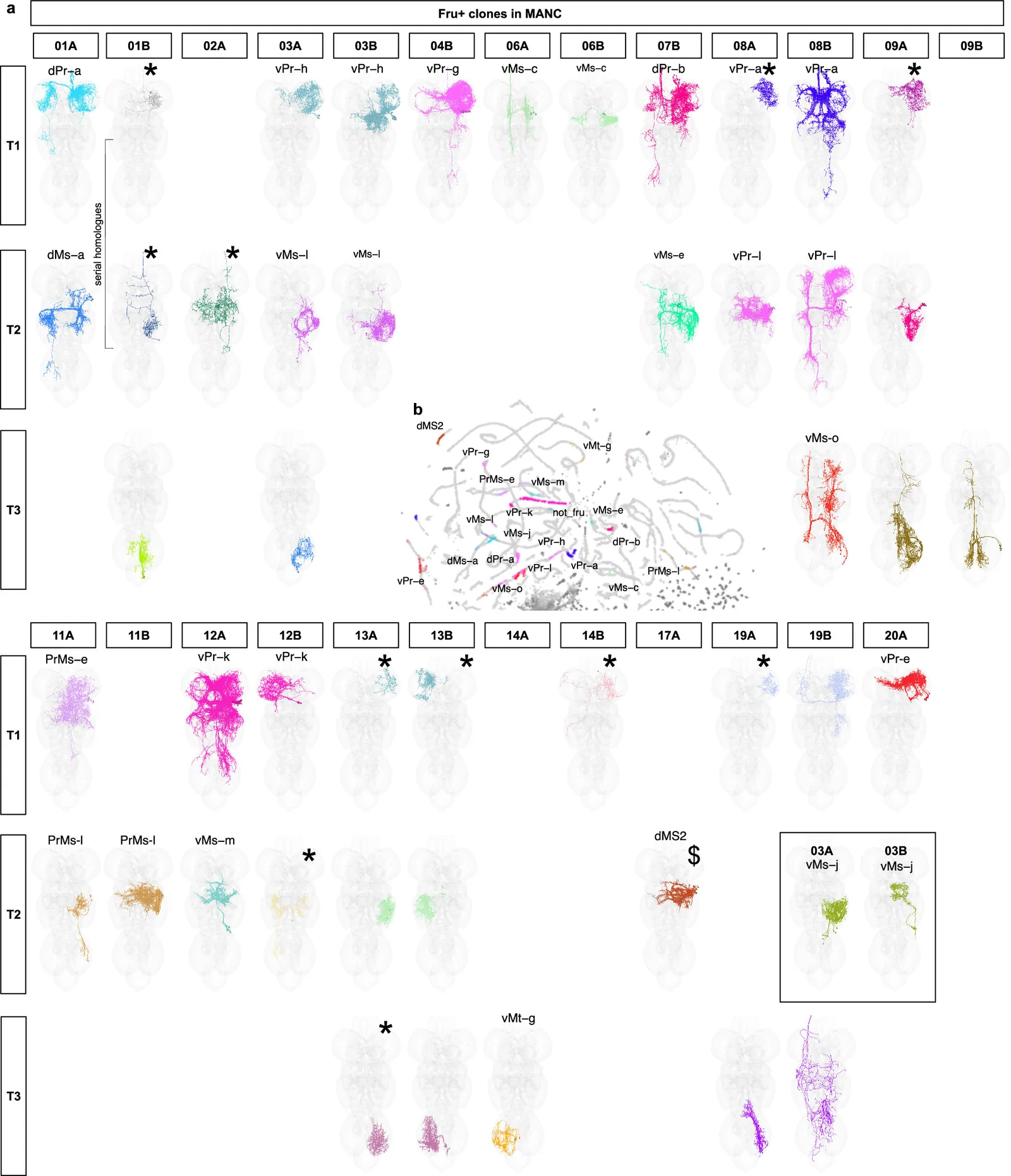
Fruitless secondary neurons in the atlas and the connectome. **a**, Neurons belonging to fru+ clones in MANC connectome arranged by lineage and soma segment of origin. When the same clone includes cells from both hemilineages, the cells in the clone have been separated accordingly. Asterisks mark clones made by a single neuron. Dollar sign refers to fru+ secondary neurons previously annotated in the connectome, not identified as fru-clones. Clone name is reported for those cross-identified between connectome and transcriptome. Two different clones mapped onto 03-T2 lineage, potentially hitting cells born in different time windows. **b**, UMAP plot showing 48 h.a.p.f. atlas cells coloured by fru clone identity.

While not all *fru*^+^ neurons belong to numerically dimorphic groups (Extended Data Fig. 8d), nearly all sex-biased secondary neurons expressed *fru* (Fig. 5c). We mapped these neurons in the MANC connectome using published light-level images of *fru*^+^ lineage-related neurons (MARCM clones)^47^ (Fig. 5f and Supplementary Table 2). By linking gene expression, hemilineage identity and neuronal morphology, we matched small groups of *fru*^+^ neurons across the atlas and the connectome (Extended Data Fig. 9). To validate our annotations we focused on *fru*^+^ neurons from hemilineage 08B-T2, which we mapped to the vPr-l clone. In the atlas, vPr-l neurons are born at three pseudotime windows: most are early born and male specific, while two later-born small groups have neurons from both sexes (Fig. 5g). Consistent with this, in the connectome, most vPr-l types are male specific, with only two types (three neurons per side) shared between sexes (Fig. 5g). Furthermore, MANC–FANC matching and intersectional labelling experiments confirmed atlas predictions of male-specific hemilineage 04B neurons in all thoracic segments, despite a corresponding clone (vPr-g) reported only in T1^47^ (Extended Data Fig. 8e,f). Notably, 04B *fru*^+^ neurons are late born in the atlas, consistent with the external position of male-specific vPr-g cell bodies in MANC (Extended Data Fig. 8e). These results showcase the atlas’s power for studying known and previously unknown sexual dimorphisms.

## Molecular mechanisms shaping dimorphism

**Extended Data Fig. 10:**
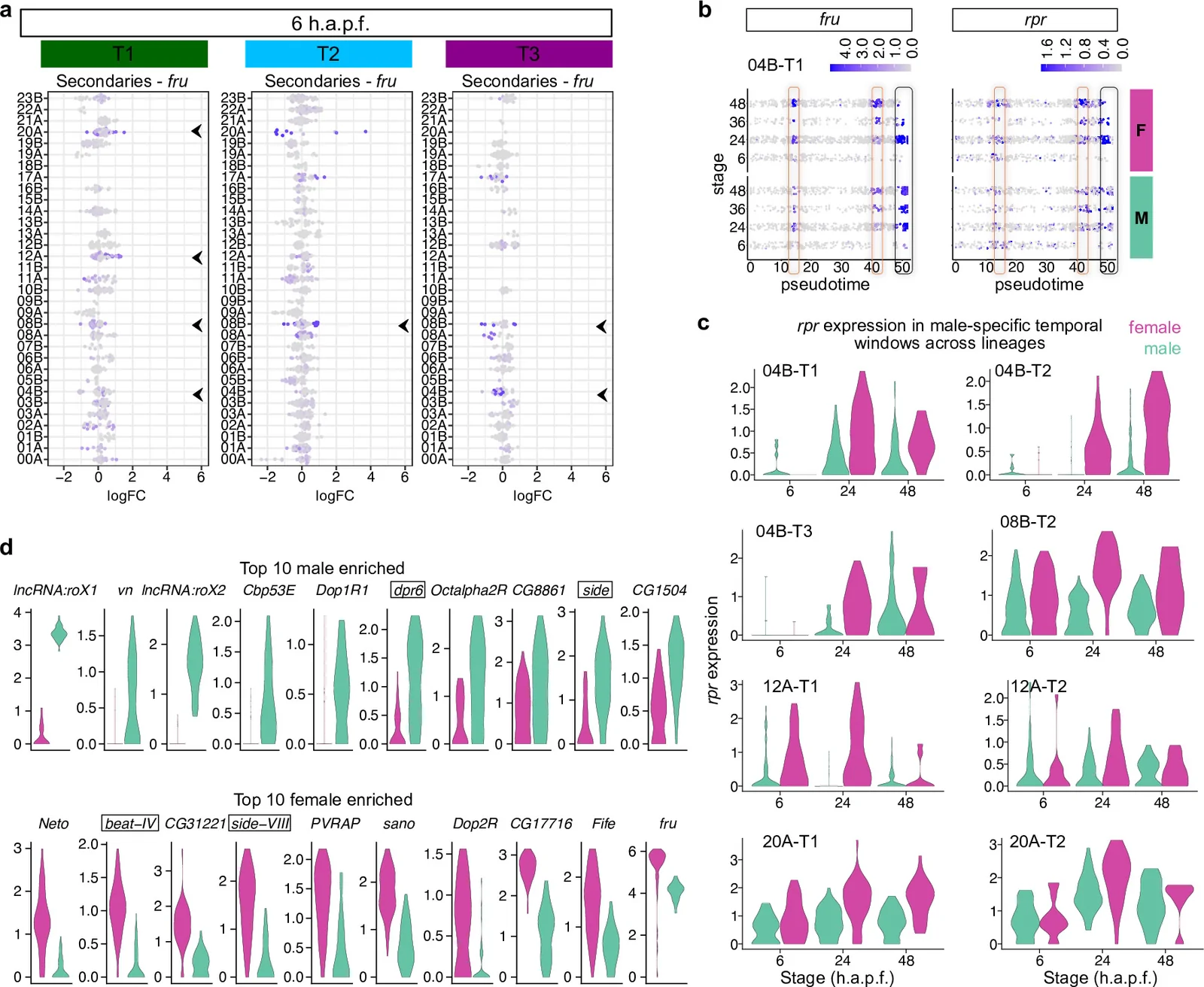
Developmental and molecular mechanisms shaping dimorphisms. **a**, Beeswarm plots of thoracic segments at 6 h.a.p.f. coloured by mean *fru* expression showing minimal differentially abundant neighbourhoods. Arrowhead points to major fru+ neighbourhoods statistically significant at 48 h.a.p.f. **b**, Dot-plot showing expression of *fru* (left) and *rpr* (right) in 04B-T1 secondary neurons arranged along pseudotime and split according to sex and animal stage. Male-enriched temporal window encircled in grey, female-enriched windows in orange. Cell bodies position in Extended Data Fig. 8e supports these predictions. **c**, Expression of *rpr* in male-enriched pseudotime windows across hemilineages. **d**, Expression of male and female enriched genes in the most dimorphic cluster in 01A-T1 at 48 h.a.p.f. (magenta: female, green: male). Boxes indicate genes with synaptic partner matching functions.

We next examined how sex differences emerge during development. The atlas shows that dimorphisms develop progressively throughout metamorphosis (compare Extended Data Fig. 10a and Fig. 5c). Accordingly, neurons within the vPr-l male-specific temporal window are gradually lost in females through pupal development. This correlates with increased expression of the pro-apoptotic gene *rpr* at 24 h.a.p.f. only in females (Fig. 5h and Extended Data Fig. 10b), suggesting that female-specific apoptosis causes the imbalanced cell number. Regions of female-specific apoptosis are present across the atlas (Extended Data Fig. 10c), pointing to its widespread role in shaping male-specific domains, extending earlier results in the brain^48,49^.

While some neurons are sex specific, others only show morphological differences^47^. To investigate the molecular signatures underlying morphological dimorphisms we focused on hemilineage 01A-T1, in which we mapped previously described sex differences in the dPr-a *fru*^+^ clone^47^ to both MANC and FANC connectomes (Fig. 5i). The atlas shows that *fru*^+^ neurons display a clear separation between males and females in 01A-T1 UMAP space (Fig. 5j). We calculated gene expression differences between sexes in clusters of the 01A-T1 lineage and identified the dimorphic cluster to have the highest number (Fig. 5j). Notably, among the most significant genes, we found several with synaptic partner matching functions, such as members of the *beat*, *side* and *dpr* families^50^ (Extended Data Fig. 10d), that might have an instructive role in dimorphic wiring. Finally, we quantified the emergence of dimorphism through metamorphosis, by defining a per-cell male–female molecular distance metric (Fig. 5j and Methods). This shows that molecular distance, and the number of differentially expressed genes (DEGs), increase with developmental time specifically in *fru*^+^ neurons (Fig. 5k).

Overall, these results show that sexual dimorphisms emerge through development from neurons with the same temporal origin by using two alternative strategies: sex specific apoptosis and transcriptional divergence. Two recent independent studies reached similar conclusions that sexual dimorphisms reflect sex-specific developmental programs acting on neurons born in the same lineage and birth order, rather than de novo generation of sex-specific cell types^51,52^.

## Discussion

Here we report a high-resolution developmental transcriptional atlas of the *Drosophila* nerve cord. This provides a platform to investigate the molecular logic of the thousands of cell types that wire up to form the sensory–motor circuits in the nerve cord, similarly to how more focussed atlases within the brain have been used to understand wiring specificity of *Drosophila* olfactory^53^ and visual circuits^54–56^. To facilitate exploration, our data are available at Scope (https://scope.aertslab.org/#/HundredDrills/*/welcome) for gene expression browsing, and on a dedicated website (https://flyem.mrc-lmb.cam.ac.uk/VNCatlas), which integrates transcriptomic and connectome metadata. We linked the atlas and the connectome by sequentially annotating primary and secondary neurons, hemilineages, segments and *fru* expression. Our description of molecular identity of consecutively born secondary neurons opens the possibility for alignment of transcriptomic and connectomic types. Comparative analysis of sexual dimorphisms in *fru*^+^ neurons refined cross-matching with the connectome to as few as three neurons (Fig. 5g). While exhaustive cross-annotation is a future goal, this correspondence for *fru*^+^ neurons shows that our atlas contains sufficient information for identification of precise connectomic cell types. Stochastic labelling of neuronal morphology^39,40^, or dense labelling of shTFs coupled with light-microscopy-based connectomics (LICONN)^57^ are promising avenues for full-scale alignment with the connectome.

Our data show that the molecular profile of consecutively born neuronal types diverge much more gradually in the larva than the embryo. Increasing the coverage of the atlas will establish whether secondary types indeed resolve in fully isolated clusters. Notably, the temporal patterning mechanisms specifying embryonic-born versus larval-born neurons mirror this distinct organization: a discrete temporal cascade of TFs in embryonic neuroblasts^4,58,59^ versus opposing gradients of the RNA binding proteins Imp (early) and Syp (late) in larval neuroblasts^60,61^. The relationship between temporal origin and molecular identity has been characterized in embryonic and optic lobe neurons^4,62^, but how *Imp*/*Syp* gradients translate to neuronal identities is only starting to be explored^42,60,63^. We propose that, by encoding a persistent molecular timestamp of neuronal birth order, the 17 shTFs are perfectly poised to be key intermediaries in this process. Although the precise functional contributions of most shTFs in specifying and maintaining neuronal identity remain unresolved, instructive roles in fate specification have been identified for some^21,52,64,65^. Our Br overexpression confirms this role in the VNC. From an evolutionary perspective, a gradual molecular identity system could offer several advantages. It is more evolvable, as small shifts in gene regulation can generate new neuron types without requiring new patterning modules, enabling gradual diversification and behavioural adaptation.

The shTFs that we identify are reminiscent of concentric genes demarcating successive cohorts of neurons in the *Drosophila* optic lobe^22,62,66^. However, the shTF code operates in the central brain and VNC, in which there are over 10,000 cell types in contrast to a few hundred intrinsic cell types in the optic lobe^13^. Our atlas provides the strongest evidence to date for a persistent molecular correlate of birth order operating across secondary neurons, extending temporal logic beyond previously studied, region-specific systems^22,27,42,62,66^. Consistent with our findings, nine shTFs had previously been identified as markers of specific projection neuron (PN) types^27^. In this context, *br* and *Eip93F* label mid-born projection neurons, whereas *danr* marks late-born projection neurons. Moreover, two recent studies have identified most shTFs in a small subset of central brain hemilineages in pupae and adults^52,67^ (Supplementary Table 3). However, beyond the 17 shTFs, other TFs identified in those studies are not broadly shared across lineages and therefore do not meet our inclusion criteria for shTFs.

Notably, the temporal specification of neurons in flies has parallels in mammals. Homologues of *Imp* and *Syp* exist and have been implicated in neurogenesis^68,69^. Furthermore, a shared transcriptional code has recently been described in maturing neurons of the mouse spinal cord, where early neurons express Onecut family TFs, intermediate neurons express Pou2f2, Zfhx3 and Zfhx4, and late-born neurons express Nfia and Nfib, often in combination with Neurod2 and Neurod6^70,71^. This evidence points to an evolutionarily conserved temporal logic governing the generation and organization of neuronal diversity.

This shared code is likely to result from the common evolutionary origin of progenitors and its conservation suggests functional importance. Shared temporal modularity could ensure stepwise circuit assembly and wiring coordination in local neuronal domains, as shown for local premotor circuits^72^, or between distant brain and VNC regions. This opens paths to study the idea that “neurons that are born together wire together”^73^. The recurrent expression of the same TF in neurons from different temporal windows could be responsible for reoccurring morphological features in temporally distant neurons within lineages^39,40^. Notably, six shTFs have roles specifying distal structures (*hth*^74^, *dan*, *danr*^75^, *bab1*, *bab2*, *rn*^76^). Proximal–distal patterning has a temporal component, with positional identity being the result of the timing of exposure to spatial patterning factors. This suggests that regulatory networks may be repurposed in different developmental contexts or a deep common evolutionary origin for neuronal and non-neuronal tissue patterning.

Many additional insights remain latent in the atlas, which will be a valuable resource not only for the *Drosophila* neuroscience community, but also for comparative studies exploring the evolutionary origins of neuronal diversity and function across phyla. It also provides a strong motivation to obtain a correspondingly high-coverage developmental brain atlas to complete the molecular description of the fly central nervous system.

## Methods

### scRNA-seq data generation

To discriminate technical from biological variability, we used flies from different genetic backgrounds^19,77^, enabling us to generate scRNA-seq libraries of VNCs of different sex and developmental stages and separate them after sequencing through single-nucleotide polymorphisms (SNP)-based sample demultiplexing (see below)^78^ (Extended Data Fig. 1). Randomized samples from different genotypes were used for the same sex and developmental stages across experiments. In total, data were collected from 92 individual animals, covering four developmental stages and both sexes. Each stage–sex condition is represented by 5 to 18 biological replicates, covering between 5 and 11 different genotypes. Some experimental preparations were used to create 2 or 3 independent libraries.

### Dissociation

For each stage–sex condition, several genotypes and individuals were used, as summarized in Supplementary Table 1. Flies of the genotypes indicated in Supplementary Table 2 were allowed to mate and their larvae developed at 25 °C until white pupae stage. Then pupae were collected with forceps, sexed under a dissecting scope and put in either male or female vials to develop until the correct stage, that is, 6, 24, 36 or 48 h.a.p.f. VNCs were dissected in ice-cold DPBS (Dulbecco’s PBS, Gibco). A maximum of 8 VNCs, each of a different genotype, was pooled in a low-bind Eppendorf tube containing 100 µl of DPBS on ice. VNCs from animals of two or three different developmental stages and sex were pulled in the same tube (6 + 24 h.a.p.f., 24 + 48 h.a.p.f. or 24 + 36 + 48 h.a.p.f.). Four brain samples were included in the last round of experiments, but later excluded from the analysis of VNC. After dissections, tissues were collected at the bottom of the tube by centrifugation (800*g*, 5 min, 4 °C), DPBS was replaced with 50 µl of dispase I (3 mg ml^−1^, Sigma-Aldrich, D4818, reconstituted in 50 mM HEPES/KOH pH 7.4, 150 mM NaCl) and 75 µl of collagenase I (100 mg ml^−1^, Invitrogen, 17100-017, reconstituted in HBSS). VNCs were dissociated in a thermo mixer (Eppendorf) at 25 °C at 500 rpm for 30 (6 + 24 h.a.p.f. samples) or 40 min (24 + 36 + 48 h.a.p.f. samples). Dissociation was aided by pipetting up and down 10 times every 10 min ensuring that the tip touched the bottom of the tube to increase the mechanical stress. Dissociated cells were centrifuged (800*g*, 5 min, 4 °C), washed once with DPBS and resuspended in 180 µl of DPBS with 0.04% BSA. The cell suspension was filtered through a 10 µm filter (pluriStrainer, Cambridge Bioscience, 43-50010-03). Viability, presence of debris and cell number were accessed using the Countess II automated cell counter (Invitrogen).

### Library preparation and sequencing

Samples were collected on 3 different days. Each day, a total of 4 different cell suspensions (independent samples) was generated, each containing different combinations of genotypes, ages and sex. Single-cell expression libraries were generated at the CRUK-CI sequencing facility using Chromium Next GEM Single Cell 3′ HT v3.1. We aimed to load each sample in duplicate, 20,000 cells per lane. Before sequencing, libraries were inspected on the TapeStation. Sequencing was performed on the NovaSeq6000 machine (Illumina) with the following sequencing parameters: 28 regular cycles of which 16 are 10x barcode and 12 are unique molecular index, 10 i7-index cycles, 10 i5-index cycles, 90 regular cycles.

### scRNA-seq data processing

No formal experimental blinding was applied for the generation and analysis of the scRNA-seq atlas; however, computational analyses were performed in an unsupervised manner, and clustering and dimensionality reduction were performed without using sample identity.

### CellRanger

Data from the NovaSeq6000 sequencer were processed using CellRanger v.8.0.0 using the cellranger count function with the default parameters. CellRanger reference index genome was built on genome assembly BDGP6.32 supplemented with the coding sequences for the expected transgenes (Supplementary Table 6). Before quality control, cell ranger total output was 805,970 cells. The median sample number of reads per cell was 39,142, the number of detected genes per cell 1,511 (min = 183, max = 8,543) and the median number of unique molecular identifiers (UMIs) per cell 4,893 (min = 666, max = 847,868).

### Demultiplexing

The output from CellRanger was demultiplexed using applications from the Demuxafy docker container v.3 (https://demultiplexing-doublet-detecting-docs.readthedocs.io/en/latest/index.html)^79^. SNP information for all sequencing reads (Pileups) were generated using cellsnp-lite (v.1.2.3) with the parameters ‘--chrom 2 L,2 R -p 22 --minMAF 0.1 --minCOUNT 100’. Out of the 4 fly chromosomes, only chromosome 2 was used for demultiplexing because, in our cross schemes, it always comes from the DGRP lines, while the remaining chromosomes originate on the other stocks. The demultiplexing package Vireo^78,79^ was run twice on the pileups, once using a variant call format (VCF) reference file and once without it (withVCF and noVCF in Extended Data Fig. 1). The VCF file for the first run was generated using the DGRP SNP data from the Aerts laboratory (https://resources.aertslab.org/DGRP2/NCSU/final/dm6/DGRP2.source_NCSU.dm6.final.SNPs_only.vcf.gz). The parameters used were ‘-N noSamples -p 4 --randSeed=1’ where noSamples is the number of different genotypes present in the given sample. The noVCF run can identify all genotypes in an unbiased way but does not provide the identity of the groups, that is, while it produces groups 1 to 7, it does not indicate which genotype each group corresponds to. Conversely, the run withVCF can identify most genotypes but is less efficient in identifying singlets due to the variable quality of VCF information across DGRP genotypes and the confounding effect introduced by DGRP heterozygosity of chromosome 2 (only one parent carried the DGRP chromosome). To use the demultiplexing results of the more efficient noVCF runs, we used several source of information to assign groups to genotypes: (1) the correspondence between expected sex and roX1 expression; (2) expression of GFP transgenes; and (3) comparison of the results from noVCF and withVCF Vireo results (Extended Data Fig. 1). In the rare cases in which the genotype assignment provided by the withVCF run disagreed with the sex or GFP transgene expression, these took precedence. Besides enabling assignment of metadata information such as sex and stage of the cell, demultiplexing also enabled removal of doublets, given that those cells have a high fraction of SNPs coming from different DGRP lines. Cells were considered doublets if they were classified as such in the Vireo noVCF run and excluded from further analysis.

### Gene annotations

The list of TFs comprises the union of genes annotated as TFs in flybase and TFs reported previously^80^. Annotation of cell surface molecules was taken from ref. ^80^. Genes involved in cellular energy production and stress response correspond to the union of flybase gene lists: CYTOCHROME_C; ELECTRON_TRANSFER_FLAVOPROTEINS; MITOCHONDRIAL_COMPLEX_III; MITOCHONDRIAL_COMPLEX_II; MITOCHONDRIAL_COMPLEX_IV_CYTOCHROME_C_OXIDASE_SUBUNITS; MITOCHONDRIAL_COMPLEX_I_SUBUNITS; MITOCHONDRIAL_COMPLEX_V_ATP_SYNTHASE_COMPLEX_SUBUNITS; heat_shock_prot.

### Quality control

The Cell ranger output was imported into an R object using sequentially the functions Read10X and CreateSeuratObject from the Seurat package (v.4)^81^ with the default parameters. Some of the last analyses were performed using Seurat (v.5)^82^. A first quality-control filter was applied to exclude cells with fewer than 700 features, more than 20% of mitochondrial genes and more than 50% of ribosomal genes. Genes expressed by fewer than three cells in the dataset were also excluded. Samples showed a bimodal distribution of UMIs and features across cells, except for SITTD8, for which the distribution was unimodal. For this reason, we excluded SITTD8 from further analysis.

### Data integration

Data integration was performed twice following the same pipeline (see below) to integrate the full dataset (including neurons and glia) as well as the subsets containing either neurons or glia.

Data integration was performed using the rpca algorithm in Seurat. In brief, 3,000 features were found using SelectIntegrationFeatures, FindIntegrationAnchors was then run on those features using the 24 h.a.p.f samples as reference and lastly, the anchors were used with the IntegrateData function with a k.weight = 100 (or number of cells −2 for small samples). This integration process removes variability due to stage and genotype. Cells with fewer than 3,000 UMIs or in clusters with an average number of UMIs smaller than 3,000 were excluded from further analysis as these were deemed to be low quality. The resulting VNC dataset has a median of 23,976.3 UMIs (min = 3,000, max = 565,460) and a median number of genes of 3,445 (min = 731, max = 8,428).

### Atlas annotation

#### Neurons and glia

To annotate neurons and glia we calculated, using the Seurat function AddModScores, enrichment scores for glial (*wrapper, repo, CIC-a, loco, CG10702, CG6126, Gs2, Egfr, Tret1-1, bdl, Zasp52, rols, ine, CG5404, CG14688, CG31663, ry, CG4752, betaTub97EF, CG32473, LManII, Eaat1, alrm, Eaat2, axo, Vmat, moody, Indy, zyd*), neuronal (*elav*,* nSyb*) or ‘other’ (*CG11835, alphaTub85E, Act57B, betaTub60D, CG5080*) marker genes. Markers for the category ‘other’ were chosen for being specifically expressed in a cell population lacking neuronal as well as glial markers. Gene Ontology analysis identified several genes enriched in cells of mesenchymal origin among markers for the other category. Categorical assignment of cells to glia, neuron or other was done based on a cluster-based winner-takes-all criterion. The category ‘other’ was excluded from any further analysis.

#### Glia types

Glial clusters were annotated according to the expression of previously reported markers^83^. Specifically *sim* expression was used for midline glia, *CG6126* for MAP, *ltl* for subperineurial, *alrm* and *Tre1* for astrocytes, *wrapper* for cortical and *axo* for ensheathing glia. Similarly to neurons and glia, glial identity was assigned based on a cluster-based winner-takes-all criterion.

#### Neurogenesis wave and maturation state

The adult brain contains primary neurons, born in the embryonic phase, and secondary neurons, born during larval stages. To identify these distinct populations in our atlas, we used the markers *Imp* (primary) and *dati* (secondary)^30,60,84^. First we assigned categorical identity to neurons expressing high levels of markers (primary, *Imp* > 2; secondary, *dati* > 2 in the stage-integrated data slot), producing nearly completely mutually exclusive labels. We reasoned that neurogenesis wave information might be more prominent in earlier developmental stages and used the obtained labels to train a classifier using the devCellPy machine learning framework^85^ with parameter rejCutoff = 0.5 and testSplit = 0.1. The trained model was used to predict primary and secondary labels across the atlas. Expression data for training and classification included all 3,000 genes from the stage-integrated data slot.

The stem region of our atlas contains cells with low expression of *dati* and markers of mature neurons, such as *brp* and *nSyb*, and it is devoid of 36 h.a.p.f. and 48 h.a.p.f. cells, suggesting it corresponds to neuronal precursors. We annotated these neurons using a similar strategy as above, but using *brp* (>1.5) for mature neurons and the chromatin remodelling factor *Phs* (>1.5) for immature ones. Similarly to the neurogenesis wave, we trained the classifier on 6 h.a.p.f. data and then used the model to predict labels across the atlas. In both cases, training and prediction were performed 50 times starting from the same training set and the consensus result was used for the final assignment.

#### Cell cycle phase

Cell cycle annotation was done using the Seurat function CellCycleScoring. Marker genes for the different phases of cell cycle were obtained from ref. ^86^.

#### Neurotransmitter identity

Each neuron in the atlas was assigned a fast neurotransmitter identity based on the highest expression level among the markers *VAChT* (cholinergic), *Gad1* (GABAergic) and *VGlut* (glutamatergic). Neurons were assigned a monoamine class (tyraminergic, octopaminergic, dopaminergic, serotonergic and histaminergic) if they co-expressed the vesicular monoamine transporter (*Vmat*) and monoamine-specific markers (tyramine: *Tdc2*^+^, *Tbh*^−^; octopamine: *Tdc2*, *Tbh*; dopamine: *Ddc*, *ple*; serotonin: *Trh*, *Ddc*; histamine: *Hdc*).

#### Hemilineage

To provide experimental evidence for hemilineage assignments, we included among the sequenced samples transgenic lines in which GFP expression is specifically driven in lineages/hemilineages (09A, 01+10B, 07B, 08B+09B). This was obtained either through split-GAL4 intersectional approach (09A, line 1, Dr-AD/Gad1-DBD; 08B + a subset of neurons from 09B, line 4, Lim3-DBD/c15-AD)^35,87,88^ or through an immortalization-based strategy (01+10B, line 2, R16A05-AD;R28H10-DBD; 07B, line 3, Dbx-DBD/ey-AD)^31,89^.

Annotation of hemilineages was performed using a stepwise approach, integrating information from multiple published and unpublished sources (summarized in Supplementary Table 4). We first assigned hemilineages with strong supporting evidence from previous studies, which helped to reduce the number of unassigned orphan hemilineages requiring further annotation. We started by mapping the adult VNC atlas^26^ onto the neuronal subset of the developmental dataset and transferring the existing hemilineage annotations to the pupal dataset using the Seurat functions FindTransferAnchors and TransferData with 3,000 variable features and 200 principal components. Then, selectively in secondary neurons, we expanded adult-derived annotation by taking an iterative cluster-based approach. Hemilineage labels were expanded to all cells within a cluster if more than 50% of its neurons were already annotated with the same hemilineage. This procedure was performed iteratively, from high clustering resolution, that is, small clusters, to low clustering resolution, that is, big clusters. Annotations were then discarded if, after the iterative procedure, they accounted for less than 20% of a cluster at an intermediate clustering resolution (resolution = 1.2). Finally, we applied a winner-takes-all strategy when the annotation represented at least 30% of the cluster at a high clustering resolution (resolution = 20). We then resolved ambiguous hemilineage identities in cases in which the adult annotation grouped two hemilineages together, but our dataset showed them as clearly separated clusters (for example, 05B/06B). We calculated DEGs between the two ambiguous clusters using FindMarkers, which confirmed cluster-specific genes and assigned cluster identity according to the relative expected hemilineage size in MANC^2^. For most hemilineages, identity was further corroborated by previously published split-GAL4 lines with experimentally validated expression patterns^35^ (Supplementary Table 4). We next annotated hemilineages not covered in the ref. ^26^ adult dataset, but expected to contain more than ten secondary neurons based on the MANC annotation. For this, we combined evidence from neurotransmitter gene expression, GFP expression from reporter lines, markers from the literature^31,80,84^, and comparison between cluster size and number of neurons expected based on electron microscopy (EM) data. Iteratively we reclustered only neurons without hemilineage annotation and assigned them to a specific hemilineage based on the expression of identified new markers. A detailed hemilineage-by-hemilineage summary of the annotation strategy used is provided in Supplementary Table 4. This led to an initial assignment of 48 h.a.p.f. secondary neurons. Annotations were discarded if they accounted for less than 30% of a cluster at an intermediate clustering resolution (resolution = 1.2). This led to the identification of high-confidence assignments (Fig. 2c) in 48 h.a.p.f. secondary neurons that were used to train a classifier using the devCellPy machine-learning framework^85^ with the parameters rejCutoff = 0.5 and testSplit = 0.1. The trained model was used to predict hemilineage labels across the atlas at all stages and expanding to primary neurons, using rejCutoff = 0.3 for prediction. Expression data for training and classification included all 3,000 genes from the stage-integrated data slot. Training and prediction was performed 50 times starting from the same training set and the consensus result was used for the final assignment.

Note that, as secondary neurons of hemilineage 15B are exclusively motor neurons, lineage propagation to primary neurons resulted in the broad assignment of 15B identity to all motor neurons, which share many molecular features. Primary 15B neurons are to be intended more broadly motor neurons.

Suboesophageal zone specific hemilineage 27X and 03A in the labium (glutamatergic in contrast to VNC homologues) were assigned based on expression of* eya*^31^ and *fd96Cb*, respectively. *fd96Cb* (CG11922) is associated with the GMR line R74D11 that drives specific expression in one hemilineage in the labium^90^.

#### Soma segment

We annotated soma segments based on Hox genes known to be expressed differentially along the VNC anterior–posterior axis (*Dfd*, *Scr*, *Antp*, *Ubx*, *abd-A* and *Abd-B*). Hemilineage-specific thresholds were needed because Antp and Ubx antibody staining in L3 larvae revealed a more complex expression landscape than expected by the canonical view (Supplementary Table 7). For example, Ubx protein levels were medium in T2 and high in T3 for 03B, but medium in T3 and high in A in 07B, reflecting expression level differences detected in the atlas^37^ (Fig. 3a,b, Methods and Extended Data Fig. 4b,c). *Dfd*^+^ and *Scr*^+^ cells belong to hemilineages originating in the gnathal ganglia (labial, maxillary and mandibular)^91^. Their presence at 6 h.a.p.f. in our dataset probably reflects imprecise excision of the VNC from the brain at this early timepoint when the neck constriction has yet not formed (Extended Data Fig. 4). We assigned all *Dfd*^+^ or *Scr*^+^ cells to GNG^91^. In those cases in which we had information about hemilineage-specific expression of *Dfd* and *Scr* in the different GNGs, we refined annotation (that is, 03B). A more detailed annotation of the GNG segments will be covered in a different manuscript (in preparation). All *abd-A*^+^ or* Abd-B*^+^ cells were annotated as A regardless of the developmental stage given that those markers are not expressed outside the abdominal segments. For the annotation of T1, T2, T3 and abdominal cells not expressing *abd-A* or *Abd-B*, instead, we first annotated cells at 48 h.a.p.f. and then transferred the annotation to earlier timepoints, due to substantial variation of *Antp* expression across pupal stages. As the expression pattern of *Antp* and *Ubx* is hemilineage specific, we treated each hemilineage independently. In cases in which immunostaining data from L3 larvae indicated that expression of *Antp* and *Ubx* genes deviated from the canonical model, we used these data as guidance. A summary of Hox gene expression derived by these data is reported in Supplementary Table 7; specific images are available on request. The workflow followed to annotate soma segments is shown in Fig. 3b. For each hemilineage, we generated violin plots showing expression by cluster at a high clustering resolution for *Antp*, *Ubx* and *abd-A*. These plots helped to choose expression thresholds to assign cells to T1, T2, T3 or A, a critical task when the difference between the different segments is dictated by the level of expression and not by the quality of the gene expressed. Thresholds used for each hemilineage are summaries in Supplementary Table 7. Thresholding based annotation was used as input for further annotation using devCellPy. Cells were excluded from the training set if their assigned label represented less than 10% of the total cells in their cluster. To prevent overtraining, only half of the labelled cells were used to train the classifier. Multilayered classification provides hemilineage-specific soma segment models which are essential to capture the hemilineage-specific Hox gene expression differences. Stage integrated expression values were used to account for expression differences due to developmental stage and allowed transfer of the model learnt using 48 h.a.p.f. data to earlier stages (as done to assign hemilineage identity). The training parameters used were: rejCutoff = 0.5, testSplit = 0.1. The obtained classifier was used to predict the full dataset with prediction parameters: rejCutoff = 0.3. Training and prediction were run 50 times starting from the same training set. Results were collated and used to obtain a consensus result used for final assignment.

We used the FindAllMarkers function to identify segment specific markers at 48 h.a.p.f. for hemilineages with complex UMAP trajectories. The 10 positive markers with the lowest* P* value, expressed in at least 80% of the cells in the segment, are shown in Extended Data Fig. 5a.

#### Fruitless clones in the atlas

To identify neurons belonging to fruitless clones in the atlas, we first selected *fru*-expressing cells using a gene expression threshold of 0.7. Next, we clustered all hemilineage–segment trajectories using the 17 shared TFs (shTFs; see the ‘Shared peak detection’ section) as input dimensions. The clustering resolution was adjusted to yield a number of clusters approximately equal to half the number of neuron types expected based on MANC annotation. Clusters were considered *fru*^+^ if at least 50% of cells were. Cells in *fru*^+^ clusters were assigned to clone identity based on matching hemilineage and segment (see the ‘Identification of neurons belonging to *fru* MARCM clones in the connectome’ for more explanations about *fru* clones).

### Primary–secondary analysis

For the analysis of primary and secondary neurons we split the dataset as follows: cells annotated as primary > primary; cells annotated as secondary or early secondary > secondary. 2,000 variable features and 200 PCs were recalculated for each subset independently in the integrated assay and used to generate the UMAP embedding shown in Fig. 1d (separated plots), using the default parameters for the appropriate Seurat functions. For cluster-size comparison to EM types and intercluster overlap analysis, we excluded 6 h.a.p.f. cells, as these were often separated from the rest of the stages, probably due to the presence of suboesophageal zone cells, cells committed to programmed cell death in the abdomen and their low degree of maturation; this incomplete integration would degrade the power of the analysis, for example, resulting in 6-h-only clusters. The atlas coverage after removal of 6 h.a.p.f. cells is 28×. We recalculated UMAP embeddings and nearest neighbours (*k* = 20) using the same 200 PCs, reasoning that the information carried by the 6 h.a.p.f. cells could help better separation of clusters. We next calculated clusters at different clustering resolutions, using Seurat FindCluster function with the default parameters, and chose resolution 16 for the analysis in Fig. 1h,i.

For the constellation plots, we first computed the number of edges between each pair of clusters based on the nearest-neighbour graph (integrated_nn). Self-edges (within-cluster connections) were excluded. In cases in which multiple cells in A had the same nearest neighbour in B, only one of those connections was retained and the rest were excluded. For each directed edge (from cluster A to B), the raw edge count was normalized to the number of cells in the target cluster (B), resulting in a directional normalized weight. To take into consideration the bidirectional comparison, we averaged the two reciprocal normalized weights between each cluster pair (A → B and B → A). This final edge weight therefore reflects the average fraction of intercluster nearest neighbours. For the graphs, we retained only edges with a weight greater than 5%. Clusters without intercluster edges after filtering were labelled in brown. For the density distribution analysis, no filtering was applied and, for each cluster, the strongest overlapping edge was retained (a measure for the distance from the closest cluster). Statistical significance between distributions was assessed using the Wilcoxon rank-sum test.

To compare the number of cells per cluster to the number of neurons per type in the connectome, we divided the cluster size by the expected coverage, therefore making the two numbers comparable. For primary neurons in MANC, we retained only neurons labelled as primary, while, for secondary neurons, we retained only those labelled as secondary. Early secondary neurons were excluded from the analysis owing to the uncertainty in assigning them with confidence to the primary or secondary transcriptional group (we believe that most primary neurons in the atlas correspond to primary + early secondary in MANC, where birthtime annotation of these neurons has low confidence). Neurons sharing the same serial annotation were considered a single type, while those lacking a serial annotation were grouped by their type label. The number of neurons in each serial/type group was then divided by two to account for the fact that coverage was calculated on the hemiconnectome.

### Trajectory analysis

#### Trajectory inference and differential expression analysis

For trajectory inference, UMAP embeddings were recalculated for each hemilineage using Seurat. Trajectory analysis was performed using Monocle3^92–95^. For each hemilineage–segment combination, we fitted a single curve and then calculated pseudotimes from first born neurons (pseudotime=0) to last born. Cells falling outside the main trajectory or in regions lacking 48 h.a.p.f. cells were removed using the Monocle3 function choose_cells. For discontinuous trajectories (that is, trajectories defined over more than one partition), gaps in pseudotimes between partitions were removed. Trajectories of secondary neurons were analysed independently for each hemilineage–segment combination. Hemilineage 15B was excluded due to the absence of a clearly defined trajectory, and 08B-T3 was omitted due to its complex and discontinuous structure. Genes differentially expressed along trajectories were calculated using the Monocle3 function graph_test and expression data from the stage-integrated slot, comprising all stages.

#### Trajectory alignment

To align trajectories, we used G2G^96^, which performs gene-level trajectories alignment. This alignment assumes that, despite differences in local dynamics, a global temporal axis exists, reflecting a consensus sequence of transcriptional states. All trajectories were compared with the reference (03A-T1, chosen as reference for being one of the longest in the dataset) using a fixed number of bins (25). We ran G2G twice with two different gene sets. For the initial alignment, we used a set of 40 TFs, corresponding to differentially expressed TFs along trajectories (q_value < 0.01, Moran’s index > 0.3) shared by at least 50% of the hemilineages, after taking the union across segments to limit the effect of short trajectories that would result in the exclusion of genes expressed at high pseudotimes values (*ab*, *Antp*, *bab1*, *bab2*, *br*, *CG14431*, *CG3726*, *CG7368*, *CG9932*, *chinmo*, *chn*, *crol*, *dan*, *danr*, *dati*, *Eip93F*, *fru*, *hang*, *HmgZ*, *hth*, *jim*, *klu*, *l(3)neo38*, *luna*, *mam*, *mamo*, *NK7.1*, *noc*, *nvy*, *Octbeta2R*, *pdm3*, *pros*, *rn*, *scrt*, *tio*, *tna*, *tsh*, *Ubx*, *zfh1*, *zld*). For the second alignment, we used only 17 shTFs (see below).

To warp pseudotimes to the reference, we fitted a linear model to the G2G output, matching bins between query and reference trajectories.

Combinations with fewer than 30% of the mean cell count across trajectories (that is, 09B–T1, 17A–T1, 09B–T3) were excluded from warping and peak conservation analysis.

#### Shared peak detection

For each trajectory, expression peaks were identified using the find_peaks function from the ggpmisc package, applied to expression profiles generated with a modified version of the Monocle3 get_fitted_values function. Peaks were calculated for each of the 40 TFs, provided that they were differentially expressed along the corresponding trajectory. Similarly, we calculated expression peaks for the average expression profile obtained by averaging expression in bins after warping trajectories to the reference space (average peaks). Average expression included only trajectories for which the given TF was differentially expressed.

For peaks matching, each reference peak was warped to the reference space and assigned the identity of the closest peak in pseudotime. A peak was considered shared if it lay within 5 pseudotime units and was at least half the height of the corresponding average peak. Genes were retained if at least one peak was shared in 50% or more of the analysed trajectories. This procedure identified 17 shTFs. For each trajectory, we ranked the positions of all trajectory-specific peaks for shTFs and computed a consensus order across trajectories using the consrank function from the ConsRank package (v.2.1.5). Trajectories with fewer than 20% of the total number of peaks were excluded from the ranking analysis (19A-T1, 22A-T1, 03B-T3).

### Central brain dataset analysis

Data for the SLPad1 trajectory shown in Fig. 5 are derived from a corresponding developmental transcriptional atlas of the brain (without optic lobes), which will be described in detail in a separate manuscript.

In brief, data collection was performed as described for the VNC, with few differences. (1) For the brain, we collected samples at 6, 24 or 48 h.a.p.f. (2) Up to 8 samples per genotype were pulled in the same tube before dissociation and a total number of 32 brains was pulled. (3) Ten independent experiments were submitted to the CRUK-CI facility for scRNA-seq using 10x Genomics Chromium Next GEM Single Cell 3′ kits (v3 or v3.1). Each sample was loaded onto one or more lanes with 10,000 cells per lane.

Downstream analysis was carried out as described for the VNC. Although a complete hemilineage annotation is not yet available for the brain, we identified a trajectory of secondary neurons marked by *Lim3* expression, with a *fru*-expressing cluster located opposite the *Imp*-positive tip, likely corresponding to the aSP-f clone. Cells along this trajectory (*Lim3*^+^*nompB*^+^ cells and *Lim3*^+^*Imp*^+^ cells) were manually selected in the stage-integrated UMAP embedding, which facilitated identification of the best-matching clusters. The manually selected cells were matched to a single cluster in the stage-integrated space (83 at resolution 1.2 using the Louvain algorithm). Cells labelled as precursors were excluded from the final SLPad1 object. Data for suboesophageal zone trajectories (03A and 03B) comprised 6 h.a.p.f. data from the VNC atlas. Cells along these trajectories were manually selected from stage-integrated UMAP embedding, to ensure inclusion of all cells. Trajectory analysis was performed as previously described for the VNC.

To calculate the expression correlation between the shTFs in the reference 03A-T1 and all hemilineages in the VNC and the 4 hemilineages from the brain, we followed:

1. Expression values were extracted from the data slot.
2. Expression was pooled in bins of 0.5 width of warped pseudotime units (that is, pseudotime values after registration to reference).
3. A Gaussian smoothing was applied using the smoother package function smth.gaussian with parameters: window = 0.2, alpha = 0.1 and tails = TRUE.
4. The correlation between each pair of shTFs from the reference and the hemilineage under analysis was calculated using the cor function from the stats package and the parameter: use = “pairwise.complete.obs”.
5. A single value for each hemilineage under analysis was calculated as the mean of the 17 correlation values.
6. To calculate the shuffled values, the process was repeated 200 times for each comparison while the identities of the shTFs in the reference were shuffled.

### Sex differences in the transcriptional atlas

#### Differential abundance analysis

Numerical differences between sexes were analysed using miloR^97^, a cluster-free method well suited to identify local differences in continuous hemilineage trajectories. We followed the pipeline suggested by developers. Stages were analysed separately and stage-integrated data were used to compute the shared nearest neighbour graph. We used the default function parameters with the exception of the following: k = 10, d = 200 for buildFromAdjacency and prop = 0.2 for makeNhoods.

#### Detection of sexual dimorphisms

We reasoned that sexually dimorphic neurons should be characterized by an increased molecular distance compared to isomorphic neurons. To quantitatively describe such distance at the single-cell level, we defined a correction score value as the sum of the differences of the per-gene expression level before and after sex-driven variability regression using the rpca integration algorithm in Seurat (similarly to what was done for stage integration). Integration was performed independently for each hemilineage–segment combination on cells from all stages. To calculate correction scores in females, male samples were kept as a reference, and vice versa. To calculate statistically significant differences between scores at 24 and 48 h.a.p.f., cells were clustered using the 17 shTFs as input dimensions as described for *fru*^+^ clone annotation. The same clusters were used to calculate DEGs between males and females at the different stages using Seurat’s FindMarkers function with the default parameters (considered significant if adjusted *P* < 0.05).

### Immunostainings and confocal imaging

Unless stated otherwise, immunohistochemistry was performed as described previously^98^. Primary antibodies were as follows: mouse anti-brp (DSHB, AB_2314866, nc82, 1:40), chicken anti-GFP (Abcam, ab13970, 1:1,000), guinea pig anti-Hth^20^ (1:500, gift from N. Konstantinides), rat anti-Dan (1:500, gift from C. Desplan), mouse anti-Br (1:50, DSHB, AB_528104, 25E9.D7), rabbit anti-Eip93F^99^ (1:2,500, gift from D. McKay), guinea pig anti-Mamo^100^ (1:1,000, gift from C. Desplan), rabbit anti-Bab1 1:1,000 and rat anti-Bab2^101^ (1:1,000, both gifts from M. Boube), rat anti-Pdm3^102^ (1:500, gift from C. Desplan).

Secondary antibodies (1:400) were as follows: Alexa-555 goat anti-guinea pig (Thermo Fisher Scientific, A21435), Alexa-555 goat anti-rat (Thermo Fisher Scientific, A21434), Alexa-647 goat anti-rat (Thermo Fisher Scientific, A21247, 714287), Alexa-488 goat anti-rabbit (Thermo Fisher Scientific, A11034, 54533A), Alexa-568 goat anti-rabbit (Thermo Fisher Scientific, A11036, 757102 or 1504529), Alexa-594 goat anti-rabbit (Thermo Fisher Scientific, A32740, 3214333), Alexa-633 goat anti-rabbit (Thermo Fisher Scientific, A21070, 751102), Alexa-647 goat anti-mouse (Thermo Fisher Scientific, A21240, 1563685), Alexa-594 goat anti-mouse-IgG1 (Thermo Fisher Scientific, A21125). Specimens were mounted in Vectashield (Vector Labs) on positively charged slides.

Confocal stacks were acquired using a Zeiss LSM 880 microscope with Airyscan and motorized stage operated by Zen 10 software at 768 × 768 pixel resolution every 1 μm (0.46 × 0.46 × 1 μm) using Zeiss EC Plan-Neofluar ×40/1.30 NA oil objective or LD LCI Plan-Apochromat ×25/0.8 NA multi-immersion objective. All images were acquired at 16-bit colour depth. Maximum projections of *z* stacks were made in Fiji.

#### Hox gene expression analysis in L3 larvae

Sparse lineage labelling was obtained through the generation of MARCM (mosaic analysis with a repressible cell marker)^103^ clones. In brief, flies of the appropriate genotypes were crossed and females were allowed to lay on grape juice agar plates for 12 h at 25 °C. After incubating at 25 °C for 12 h, the embryos were heat-shocked in a 37.5 °C water bath for 30 min, rested at room temperature for 30 min, then heat-shocked again for 45 min. Larvae were reared on 4–24 instant fly medium (Carolina Biological Supply) at 29 °C to increase expression of the GAL4^C155^ driver and visibility of MARCM clones. The w, GAL4C155, hsFLP, UAS-mCD8::GFP; FRT82B; tubP-GAL80 stock was shared by J. Parrish and the P{ry[+t7.2]=neoFRT}82B ry[506] stock was obtained from the Bloomington Drosophila Stock Center (Indiana University).

Nervous systems were dissected from wandering third instar larvae of both sexes in PBS (pH 7.2), fixed in 3.7% buffered formaldehyde at room temperature, then washed in 0.3% PBS-TX (PBS with 0.3% Triton X-100). Fixed samples were blocked in 2% normal donkey serum (Jackson Immunoresearch Laboratories) in PBS-TX for 30 min, then incubated for several days at 4 °C with primary antibodies: rat anti-mCD8 (Thermo Fisher Scientific, MCD0800, 5H10, 1:100), mouse monoclonal anti-neurotactin (BP106, 1:30) or mouse monoclonal anti-Antp (DSHB; AB_528083; 8C11, 1:500) and rabbit anti-Ubx (7701, 1:1,000)^37^. After primary antibodies were washed out in PBS-TX at room temperature, tissues were incubated overnight at 4 °C in a 1:300 dilution of Alexa 488-conjugated donkey anti-rat IgG (Thermo Fisher Scientific, A21208), Alexa-555-conjugated donkey anti-mouse IgG and/or Alexa 647-conjugated donkey anti-rabbit IgG (Thermo Fisher Scientific, A31570 or A31573). After additional washes in PBS-TX at room temperature, tissues were arranged on polylysine-coated coverslips, cleared in xylene and mounted in DPX (Sigma-Aldrich).

Slides were imaged using a ×40 oil objective on a Leica SP5 Spectral Systems confocal microscope. *z* stacks were collected sequentially with averaging at 0.5 to 1.0 μm intervals. Raw data stacks were imported into Fiji (https://fiji.sc/) and either merged or projected into 3D representations for lineage identification based on morphology, neuroblast location, projections into a landmark neurotactin scaffold and/or *Ubx* expression, using our published atlases^32,36,37^. Confocal stacks were processed and assembled into figures using Fiji and Affinity Publisher. Images are shown as maximally projected 2D views of subsetted confocal stacks, without removing potentially obscuring clones that can be resolved in 3D representations.

#### Validation of segment annotation

VNCs were dissected and fixed as previously described^35^. Primary antibody staining was performed sequentially: samples were incubated overnight at 4 °C with mouse anti-brp (1:25, DSHB, nc82) and either preabsorbed chicken anti-GFP (1:1,000, Thermo Fisher Scientific, A10262) or rabbit anti-GFP (1:1000, Thermo Fisher Scientific, A11122), followed by an overnight incubation with rabbit anti-tey^104^ (1:200, gift from A. Stathopoulos) or guinea pig anti-kn^105^ (1:200, gift from W. Moore), respectively. Corresponding secondary antibodies (1:500; goat anti-mouse IgG Alexa Fluor 568, Thermo Fisher Scientific, A11001; goat anti-chicken IgY Alexa Fluor 488, Thermo Fisher Scientific, A11039, 488734; goat anti-rabbit IgG Alexa Fluor 488, Thermo Fisher Scientific, A11034, 54533A; goat anti-rabbit IgG Alexa Fluor 633, Thermo Fisher Scientific, A21070, 751102; or goat anti-guinea pig IgG Alexa Fluor 647, Thermo Fisher Scientific, A21450) were applied overnight at 4 °C. The samples were washed in PBS plus 0.1% Triton X-100, positioned onto lysine-coated coverslips and dehydrated through an ethanol series, cleared in xylene and mounted in DPX^36^. Imaging was performed using the Nikon ECLIPSE Ti2 confocal microscope equipped with a Nikon Plan Apo ×40 oil-immersion objective. Confocal stacks were acquired at 1,024 × 1,024 resolution with sequential scanning and *z*-steps of 0.5–1.0 µm.

#### EdU labelling

EdU labelling experiments were performed as described previously^106^. In brief, newly hatched larvae were collected for 1 h, transferred to fresh food and raised at 25 °C until the appropriate stage for the EdU pulse. Larvae were then transferred to EdU-conditioned food and allowed to eat for 7 to 8 h. After the pulse, larvae were transferred to fresh unconditioned food until eclosure. Adult brains and VNCs were dissected, fixed and stained. EdU detection with Click-iT EdU Imaging Kit with Alexa Fluor 647 (Thermo Fisher Scientific) was performed according to the manufacturer’s instructions and the following immunostaining was performed as previously described^98^. Primary antibodies: mouse anti-Br-core monoclonal (1:50, DSHB, AB_528104, 25E9.D7) and rabbit anti-Bab1 polyclonal (1:1,000). Secondary antibodies: Alexa-594 goat anti-mouse-IgG1 (1:1,000, Thermo Fisher Scientific, A21125) and Alexa-594 goat anti-rabbit (1:1,000, Thermo Fisher Scientific, A32740, 3214333). Confocal images were acquired using the Leica TCS SP8 3× gated STED confocal microscope equipped with a white light laser using optimal excitation frequencies for each fluorophore and a ×40/1.1 NA water objective. Care was taken to acquire channels independently to avoid bleedthrough between 594 and 647 fluorophores. Confocal stacks tiling the VNC and brain were stitched either by the microscope software with the default parameters or using the pairwise stitching plugin in Fiji with the default parameters^107^. EdU experiments were analysed following an automated pipeline, independent from sample identity. Fluorescence signals for Br, Bab1 and EdU were segmented using channel-specific Ilastik models (Ilastik v.1.4.1)^108^ trained on multiple images. Simple Segmentation outputs were generated from the Pixels Classification pipeline and transformed into binary masks in Fiji. Expression overlap was quantified as the number of pixels in the confocal stack positive for both shTF and EdU, divided by the total number of shTF pixels.

### Connectomics data analysis

Summary statistics refers to MANC v.1.2.3 (root node: 1ec355123bf94e588557a4568d26d258). Data are available from https://neuprint.janelia.org. The total number of annotated intrinsic neurons (that is, neurons with soma in the VNC volume, corresponding to classes ascending neuron, efferent ascending, efferent neuron, intrinsic neuron, motor neuron) is 15,760. Throughout the analysis, numbers refer to one side, under the assumption that the VNC is bilaterally symmetric and that the same number of neurons is expected on each side. For example, to calculate aggregate coverage: 302,765/7,880 = 38.4.

For the analysis and visualization of connectomics data, we used packages from the natverse toolbox^109^. Dataset-specific packages for the male VNC (MANC, https://github.com/natverse/malevnc), the female VNC (FANC, https://github.com/flyconnectome/fancr) and the female brain (fafb/flywire, https://github.com/natverse/fafbseg) were used.

#### Similarity to nearest neighbour

We downloaded skeletons for all neurons in the MANC dataset, created dotprop representations and used them to run an NBLAST^110^ comparison. The scores for the best matches for each primary and secondary neuron were used to create the morphological comparison. Similarly, we downloaded the connectivity information for all neurons in the MANC dataset and used it to create cosine similarity comparisons within neurons from the left side and within neurons from the right side. The scores for the top matches for each primary and secondary neurons were then used to create the connectivity comparison. The statistical significance of observed differences was evaluated using a Wilcoxon rank-sum test.

#### Identification of 14A types matching light-level stainings

To identify the neurons shown in Fig. 5f, we queried Clio for all neurons annotated as hemilineage 14A, soma_side = RHS, soma_neuromere = T1 and birthtime = secondary or early secondary. We manually excluded neurons of which the projections did not match the morphology observed in light-level images, yielding 17 seed neurons (IDs: 17237, 21776, 23665, 23829, 24397, 25482, 25805, 29164, 31376, 32854, 34040, 42121, 43194, 46063, 103686, 155414, 166875). All neurons sharing the same systematic_type annotations as these seeds were selected to generate the set shown in Fig. 5f (left). We then identified additional neurons that follow the same axonal bundle as the seed set (reduced in the *Br*-overexpression condition). Finally, the remaining 14A, RHS, T1 secondary/early-secondary neurons were used as seeds to select contralateral neurons and neurons in other segments with matching systematic_type annotations, generating the dataset shown in Fig. 5f (right).

#### Male–female connectome matching

The lack of extensive neuronal typing of the female EM dataset (FANC) or matching to homologous male neurons in MANC, motivated us to develop an algorithm that uses a two-stage pipeline to match single neurons from one datasets to one in the other. This enables the study of homologous neurons, the identification of potentially dimorphic neurons, with low score or missing altogether, and the transfer of cell type labels from MANC to FANC.

In the first stage of the process, a matching algorithm assigns neurons maximizing morphology similarity (metric: NBLAST) across the dataset. In the second stage, a new assignment round, constrained by morphology similarity from the first round, aims to maximize connectivity similarity (metric: cosine similarity).

Assignment based on morphology > NBLAST matches:

1. Neuronal skeletons were downloaded for both MANC and FANC datasets.
2. FANC skeletons were transformed into MANC space^111^.
3. Skeletons were converted to dotprops.
4. Morphological similarity was calculated between neurons from each dataset as the mean normalized NBLAST score.
5. An implementation of the Hungarian algorithm calculated NBLAST matches between MANC and FANC neurons as those that maximized the global similarity between datasets, as measured by NBLAST scores from the assigned neurons.

Assignments by connectivity > connectivity matches:

This assignment requires previous knowledge of neuronal identity, that is, to evaluate how similar the connectivity of one neuron in MANC is to a neuron in FANC the identity of their partners, at least partially, must be known.

1. We established an initial panel of 50 homologous neurons between MANC and FANC to be used to calculate cosine scores between neurons from both datasets. These included highly similar neurons with a high NBLAST score) and a single good match; thus, bona fide homologues. We then selected the 2,000 neurons with the greatest difference between the top two NBLAST scores, a proxy for match uniqueness. From these, we selected the 1,000 neurons with the highest number of synapses, with the rationale that a large synapse count correlates with large neuronal size, and this, in turn, is more likely in primary neurons, which tend to be unique and have single matches across datasets. Neurons with many synapses are more useful to probe connectivity, as they connect to the largest possible fraction of unlabelled neurons. From these 1,000, we randomly selected a subset of 50.
2. In each dataset, we identified the 500 neurons most connected to the panel of 50.
3. We then computed cosine similarity between MANC and FANC, within these panels of 500 neurons, considering only connections to the panel of 50 neurons.
4. We matched MANC to FANC neurons based on the cosine scores, but only retained matches with a high NBLAST score (as a fraction of the top NBLAST match, for example, if a neuron’s top match had a score of 0.8, then only neurons with NBLAST scores >0.8 × threshold would be considered) to ensure both high connectivity and morphological similarity. We therefore transferred homology labels from MANC to FANC, increasing the panel of labelled neurons from 50 to 550.
5. We repeated steps 2, 3 and 4 until all neurons in the dataset were analysed.
6. The NBLAST threshold used in 4 was progressively lowered to include additional match candidates until a predefined minimum threshold was reached. For the table below thresholds used went from 0.95 to 0.65 in 0.1 decrements.
7. The best one-to-one matches were recorded as the final connectivity matches and the full history of match assignments was noted.

When compared with manual assignments between FANC and MANC recently published for ascending neurons^17^, the automatic assignment algorithm finds matches that in 85% of the cases are in agreement with the manual type assignment.

The results of the matching are provided in Supplementary Table 8.

#### Hemilineage annotation of FANC neurons

To identify all neurons belonging to selected lineages in FANC, we started from best matches from the MANC–FANC automatic matching results (same match for NBLAST and connectivity and connectivity match score > 0.3). This enabled us to identify seed planes in the FANC EM volume containing most or all of the selected IDs from each hemilineage (typically the soma tract, or a point of entry to, or exit from, the VNC). Any extra neuron in the seed plane that appeared to belong to the bundle formed by the neurons in the hemilineage was marked as a potential candidate to belong to the hemilineage. Any obvious outlier was marked as an outlier and any neuron obviously belonging to other hemilineages was marked as ‘other’. Any neuron of which the morphology was not clear due to a necessity for proofreading (for example, loose soma, massive glia) was added to the list of neurons to be proofread, to ensure correct inclusion/exclusion from the hemilineage of interest. As one seed plane might not contain all of the neurons of a given hemilineage, extra seed planes were considered. All newly found neurons were coarsely proofread. After proofreading, neurons were rematched to MANC and the final list of potential hemilineage markers was manually curated to obtain a final annotation, reported in Supplementary Table 9.

#### Identification of neurons belonging to *fru* MARCM clones in the connectome

Previously published confocal stacks of segmented *fru*^+^ MARCM clones in VNCIS1 reference space^47^ were transformed to MANC space using functions from packages CMTK (v.3.4.0)^112^ and navis (v.1.10.0)^113^. In brief, .nrrd files were read with navis.read_nrrd, thresholds were identified using numpy.quantile (numpy v.1.24.2) for the 99% and the images were then converted to meshes using the function navis.mesh and the calculated threshold. The new meshes in VNCIS1 space were processed using navis.xform_brain with the parameters source VNCIS1 and target MANC. The resulting meshes were converted to neuroglancer precomputed objects using navis.write_precomputed. These were used to implement a neuroglancer (https://github.com/google/neuroglancer/) layer that could be visualized in MANC space.

We identified MANC neurons potentially belonging to fruitless clones on the left hemisphere. For each clone, we first determined its lineage by loading neurons from candidate lineages into neuroglancer alongside the corresponding clone mesh. We looked for overlap of soma tracts. Once the lineage had been identified, we loaded all neurons from one hemilineage at a time and discarded those with processes outside the mesh. The remaining neurons were considered the best candidates (seed neurons). When possible, we tried to identify as many seed neurons as predicted by light-level clones. To refine the selection, we took a by-type approach: neurons were retained if at least 50% of the neurons in type were included in the seed (exception: Tr flexor motor neuron in dPr-e, as this type comprises neurons from different lineages). When this procedure retrieved neurons with processes clearly outside the mesh, the type was manually excluded. When the number of neurons retrieved largely exceeded the expected number, EM meshes for individual types were downloaded, converted to VNCS1 space and co-visualized with the confocal stack of the clone in VVD viewer (v.1.7.4)^114^. In many cases, this allowed the exclusion of types definitely absent from the clone. For each clone, we provide a confidence score (1, almost certain; 2, good guess; 3, putative). At the end of this process, we expanded annotation to the right hemisphere assigning clone identity to neurons from the same group in MANC metadata. A summary of fruitless neurons annotation is provided in Supplementary Table 5.

#### Identification of sex-specific or dimorphic neurons

MANC neuronal types were considered male specific or dimorphic if none of the neurons within the type had a FANC match with connectivity score greater than 0.3. We decided to perform a type-level analysis because it is more robust than per-neuron analysis. Owing to the incomplete proofreading status of FANC it is not uncommon to obtain matches on one side but not the other. Moreover, the matching algorithm can only assign a single match, which would result in unmatched neurons if a type has a different number of neurons between males and females. This level of dimorphism would need to be treated differently.

For female-specific neurons in 01A T1, we identified all FANC neurons manually annotated by us to belong to that hemilineage that were not matched to any MANC neuron. We then manually curated the remainders to retain only those neurons with a clear contralateral match.

### Fly husbandry

All flies were raised in vials of Iberian medium at 25 °C under a 12 h–12 h day–night light cycle. A list of all *Drosophila* stocks used in this study is reported in Supplementary Table 10.

### Reporting summary

Further information on research design is available in the Nature Portfolio Reporting Summary linked to this article.

## Online content

Any methods, additional references, Nature Portfolio reporting summaries, source data, extended data, supplementary information, acknowledgements, peer review information; details of author contributions and competing interests; and statements of data and code availability are available at https://doi.org/10.1038/s41586-026-10797-w.

## Supplementary information

## Data Availability

10x scRNA-seq data collected in this study (FASTQ files, CellRanger outputs and processed and annotated Seurat objects) are available at the Gene Expression Omnibus (GEO) under accession number GSE304221, and at BioProject under accession number PRJNA1297747. Annotated loom files are also accessible at Zenodo (full dataset^115^: https://zenodo.org/records/17183028; neurons^116^: https://zenodo.org/records/17184089; glia^117^: https://zenodo.org/records/17185432) and at SCope (https://scope.aertslab.org/#/HundredDrills/*/welcome), an interactive web platform for exploring single-cell datasets. The SCope interface allows rapid inspection of marker genes and cell-level information, making it straightforward to interact with the data. Data presented here, including links between the atlas and the connectome, can also be browsed on a dedicated webpage (https://flyem.mrc-lmb.cam.ac.uk/VNCatlas). Previously published 10x scRNA-seq data used in this study^26^ are available at GEO under accession number GSE141807. Additional imaging data are available on request.

## Code availability

Code with instructions to download and inspect data from GEO and SCope is available at GitHub (https://github.com/FlyNeuroAtlas/DevSeqVNC). Additional code is available on request.
