# Supplementary material for: A global molecular code for birth order and neuronal identity in Drosophila
Source: Nature. 2026 Jul 22;657(8130):202–12. doi: 10.1038/s41586-026-10797-w (PMC13538039; doi:10.1038/s41586-026-10797-w)
Supplement: Supplementary file 1 [file 41586_2026_10797_MOESM1_ESM.pdf]

nature portfolio

https://doi.org/10.1038/s41586-026-10797-w

Supplementary information

A global molecular code for birth order and neuronal identity in *Drosophila*

In the format provided by the authors and unedited

A global molecular code for birth order and neuronal identity in *Drosophila*

Sebastian Cachero[*†1], Myrto Mitletton[1], Isabella R. Beckett[1], Elizabeth C. Marin[2], Laia Serratosa Capdevila[3], Marina Gkantia[2], Jelly H.M. Soffers[4], Haluk Lacin[4], Gregory S.X.E. Jefferis[†1,2], Erika Donà[*†1,5]

[1]Neurobiology Division, MRC Laboratory of Molecular Biology, Cambridge, UK

[2]Department of Zoology, University of Cambridge, Cambridge, UK

[3]Aelysia Ltd, Bristol, UK

[4]School of Science and Engineering, Division Biological and Biomedical Systems, University of Missouri-Kansas City, MO, USA

[5]Institute of Neuroscience, Consiglio Nazionale delle Ricerche (CNR), Vedano al Lambro (MB), Italy

[*] contributed equally

[†]correspondence to: scachero@mrclmb.ac.uk, jefferis@mrc-lmb.cam.ac.uk, erika.dona@cnr.it

Supplementary Results and Discussion

Glial cells

Our atlas comprises both neurons and glia cells. In one of the first steps of our analysis, we annotated both cell types and subsetted the atlas, focussing most of our work on neurons and performing only limited analysis on glia (Supplementary Fig. 1a). We found that the ratio of glia to neurons moderately but significantly increased from 6 to 48h (Supplementary Fig. 1a), in agreement with reports of gliogenesis occurring in early pupal stages[118], and the presence of actively dividing glial subpopulations in our atlas (Supplementary Fig. 1f-h). Interestingly, unlike the neuronal subset, developmental stage variability regression in the glia subset did not eliminate inter-stage differences, indicating that glial cell identities are substantially more variable across development than neuronal identities. While a detailed characterisation of the glial types present in this dataset is beyond the scope of this work, we annotated six main glial types based on previously reported markers, with many more subtypes (or states) evident in the atlas (Supplementary Fig. 1, Methods). This glial subset, of equal quality as the neuronal subset, constitutes a useful resource for studying their development and their role during brain development.

Aminergic modulation

Besides the fast acting neurotransmitters acetylcholine, GABA and glutamate (Supplementary Fig. 2a-b) we annotated the atlas for modulatory neurons based on the expression of essential biosynthetic enzymes and the vesicular monoamine transporter *Vmat* (Supplementary Fig. 2c-d). Differently from fast neurotransmission, aminergic modulatory neurotransmission is restricted to a small number of primary neurons (Supplementary Fig. 2d). For fast neurotransmitters, our atlas confirms the connectome predictions that, across all neurons, excitatory acetylcholine is the most common, followed by GABA and glutamate[2].

Segment identity validation

To validate NM annotations we tested the expression pattern of the segment-specific markers *tey* (expressed in hemilineage 03B and 07B) and *kn* (expressed in hemilineage 07B) (Extended Data Fig. 5 and Supplementary Fig. 4). As predicted, *tey* expression in hemilineages 03B and 07B was limited to the abdominal segment, in contrast to hemilineage 12B, where its expression spanned T1, T2 and T3 segments (Supplementary Fig. 4a-d). Expression of kn, instead, was limited to a small subset of T1 neurons and a large subset of T2 neurons in 07B, in agreement with our prediction (Supplementary Fig. 4e). Segment annotation is not perfect: while we predicted a very small number of tey+ 07B-T3 neurons, we did not detect any. As these cells arrange along an otherwise abdominal-specific trajectory, it is possible that they belong to the abdominal segment despite having lower Ubx levels.

Hemilineage specification via Notch signalling

Observations from the optic lobe, where, with rare exceptions[119], NotchON neurons have been reported to share the expression of the TF Apterous[22,62,120], prompted us to investigate whether any shared

transcriptional signature existed in the VNC across NotchON (A) or NotchOFF (B) hemilineages. However, markers associated with Notch identity (A/B), did not show a universal post-mitotic signature across lineages (Supplementary Fig. 3). Additionally, in contrast to the optic lobe, Apterous marks a NotchOFF hemilineage in the VNC and, more broadly, neurotransmitter identity does not segregate consistently with Notch status, with both NotchON and NotchOFF neurons adopting excitatory or inhibitory fates in a lineage-dependent manner. Together, these observations indicate that Notch-dependent diversification is not accompanied by a conserved global transcriptional program in post-mitotic neurons in the VNC.

Two modes of neuronal specification

In this work we describe a pronounced distinction between primary and secondary neurons in the way they distribute in UMAP space. While the distinction between primary and secondary neurons could reflect differences in maturation, with primary neurons being born earlier, three lines of evidence argue against it. Firstly, the time difference between the birth of primaries and that of early-secondary neurons is smaller than between the latter and late-secondary neurons; secondly, similar differences between Imp+ and Imp- cells are also present in a recently published atlas of the adult CB (ref. to Fig. 2d and Fig. 2k in Allen et al. 2025); and, thirdly, we also detect corresponding morphological and connectivity differences in the adult connectome. These observations imply that UMAP structure reflects fundamentally different developmental programs rather than degrees of maturation. Based on these observations, we propose that studies in *Drosophila* may be relevant beyond flies, helping elucidate the developmental logic of contiguous neuronal types, previously associated with mammalian nervous systems[7,121,122].

Supplementary Figures

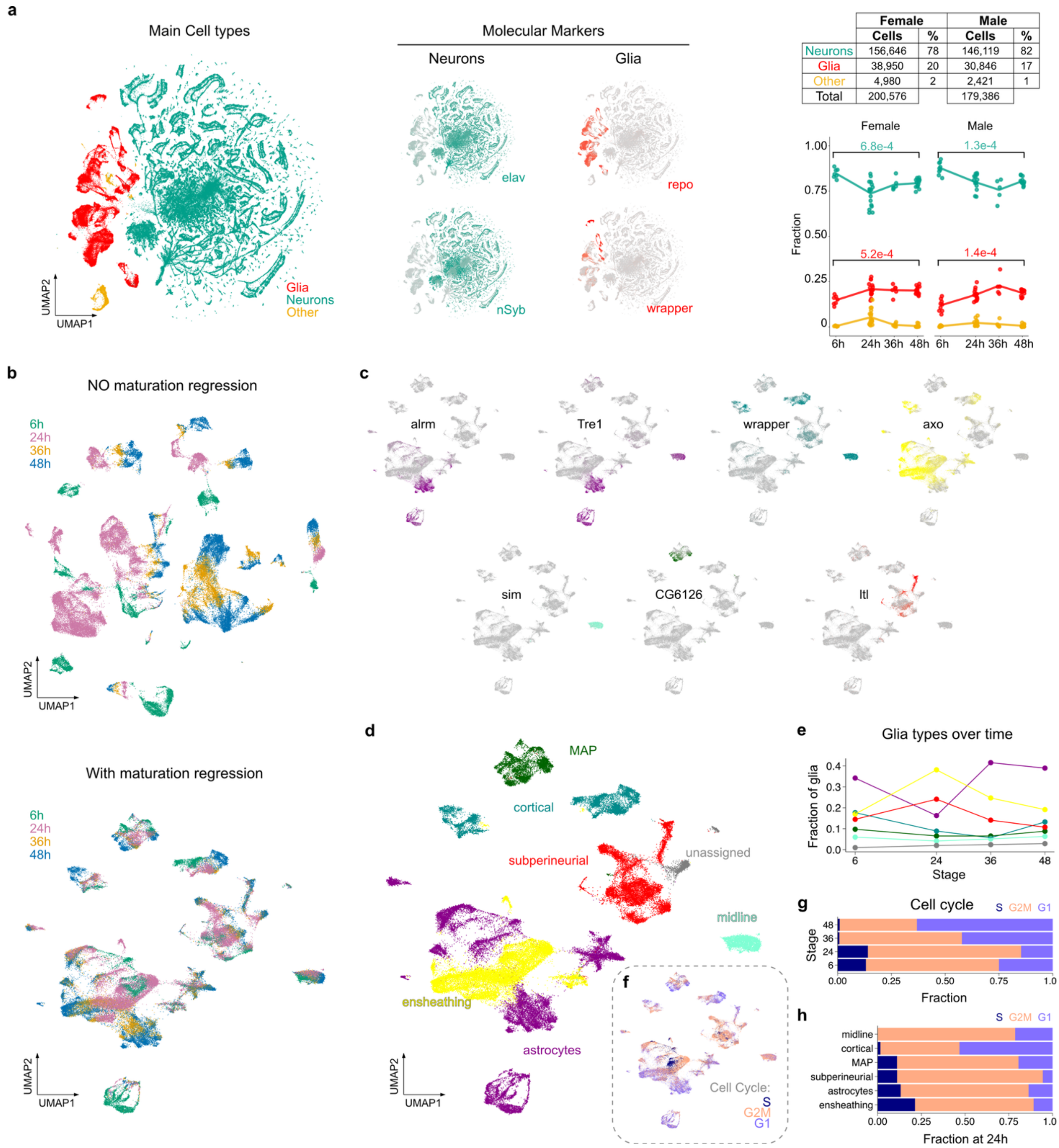

Supplementary Fig. 1 | Developmental atlas of VNC glia

a, Left: Atlas annotation by glia, neurons and other (cells of mesenchymal origin). Center: UMAP plots showing the expression of glial and neuronal marker genes. Right: Table showing the number of glia, neurons and other cell types after demultiplexing and quality control. The graph shows the fraction of each population across sampled stages, stratified by sex. Each point corresponds to a single animal; brackets indicate statistical significance assessed using a two-sided Student's t-test. For the number of observations, see independent samples column in Supplementary Table 1 **b,** UMAP plot of VNC glia from 6, 24, 36 and 48 h.a.p.f. without (top) and with (bottom, used in the rest of the plots) regression of maturation-driven variability. Cells are coloured by the developmental stage of the animal they originate from. **c,** UMAP plots showing expression pattern of known markers of glia cell types. **d,** UMAP plot showing glia cell types annotation based on markers in (**c**). **e,** Relative abundance of glia types over time. Colours as in (**d**). **f,** UMAP plot showing the result of cell cycle phase analysis. **g,h**, Stacked bar chart showing the fraction of glial cells in each cell cycle phase over time (**g**) and per glia type at 24 h.a.p.f. (**h**).

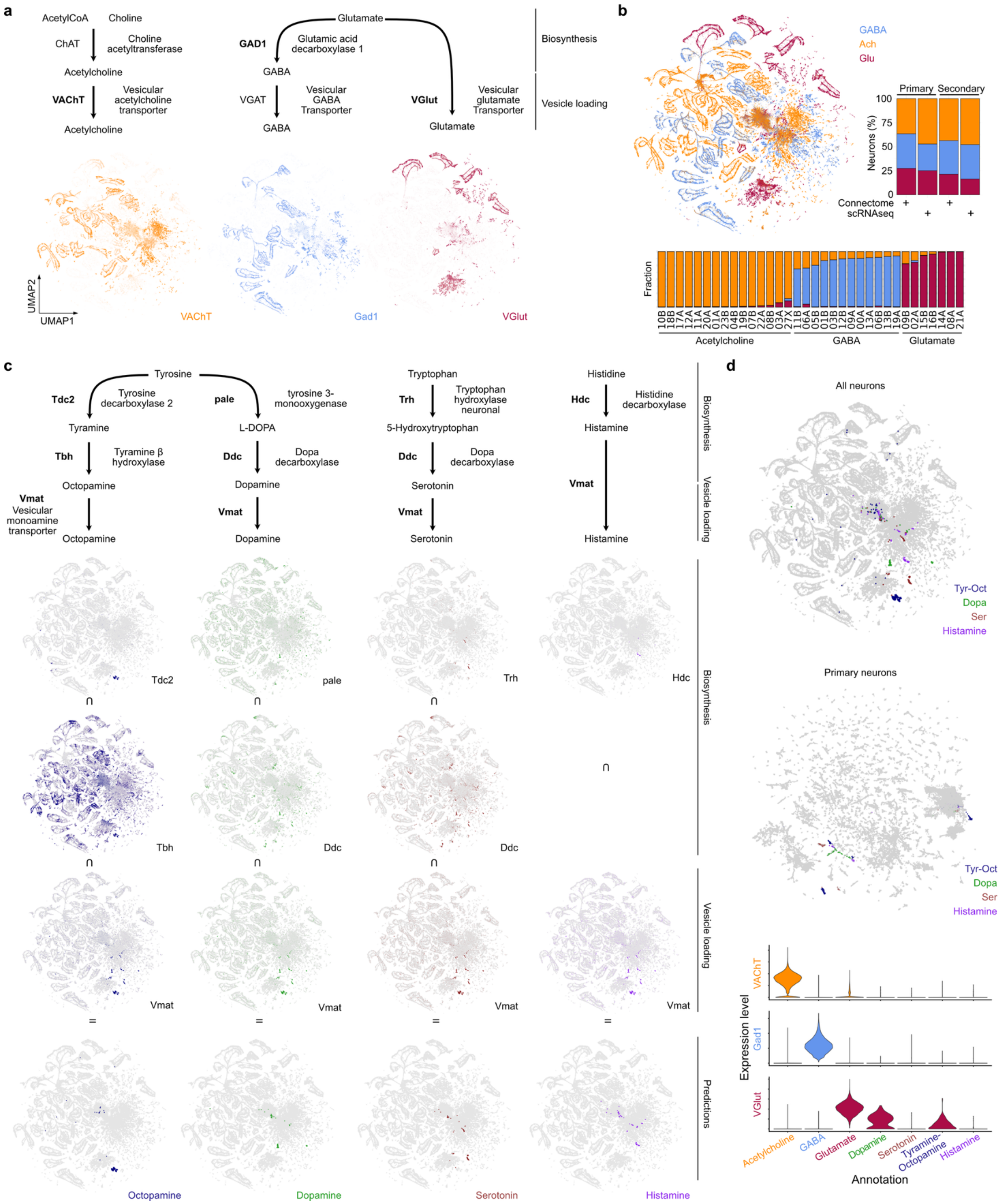

Supplementary Fig. 2 | Predictions for neurotransmitter usage

a, Top: Fast neurotransmitters biosynthetic pathways. Bottom: UMAP showing expression levels of enzymes used to predict neurotransmitter usage. **b**, Top: UMAP showing fast neurotransmitter assignments defined by the most highly expressed enzyme in (**a**) after scaling. Fraction of fast neurotransmitter identities predicted in primaries and secondary+early secondary neurons in the atlas (48 h.a.p.f.) and the connectome. Bottom: fraction of cells using each neurotransmitter system per hemilineage. **c**, Top: Aminergic neurotransmitter biosynthetic pathways. Bottom: UMAP showing expression level of enzymes and the shared vesicular monoamine transporter (Vmat) used for

synaptic loading. **d,** Top: UMAP plots showing aminergic predictions resulting from intersection of biosynthetic enzymes and transporter expressions for all neurons and primary neurons only. Bottom: Violin plots show the expression of fast neurotransmitter enzymes and transporters in neurons using different fast and aminergic neurotransmitters.

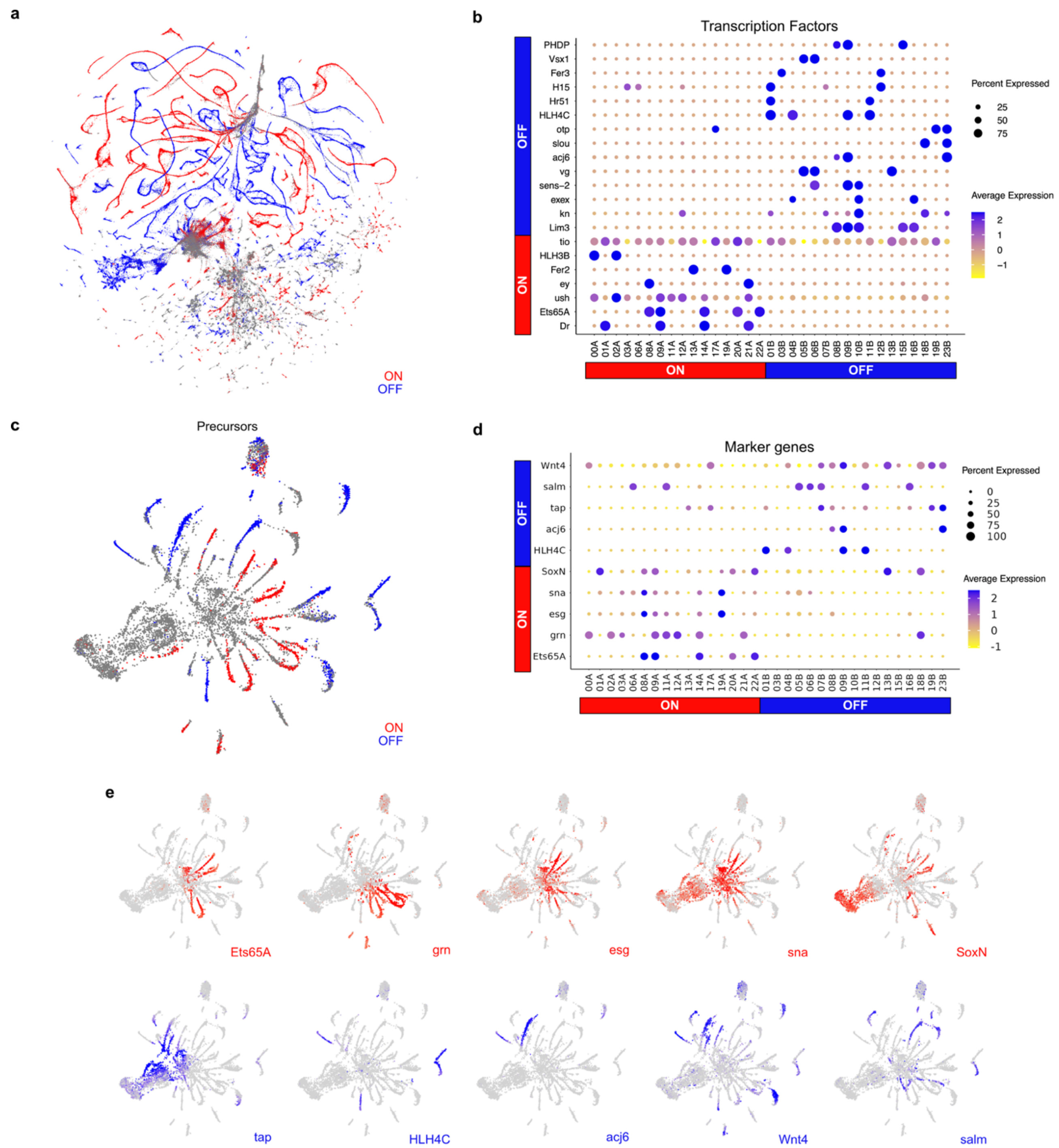

Supplementary Fig. 3 | Markers correlated to Notch signalling

a, UMAP plot showing the identity of Notch ON (A) and Notch OFF (B) hemilineages. **b,** Dot-plot showing expression in hemilineages of TFs identified as expressed preferentially in cells from Notch ON vs Notch OFF hemilineages. **c,** Same as (**a**) but in precursor cells (6 and 24 h.a.p.f.). **d,** Same as (**b**) but in precursors. Analysis expanded to all markers, not only TFs. **e,** UMAP plots showing expression of the top 5 markers for Notch ON and Notch OFF neurons in precursor cells.

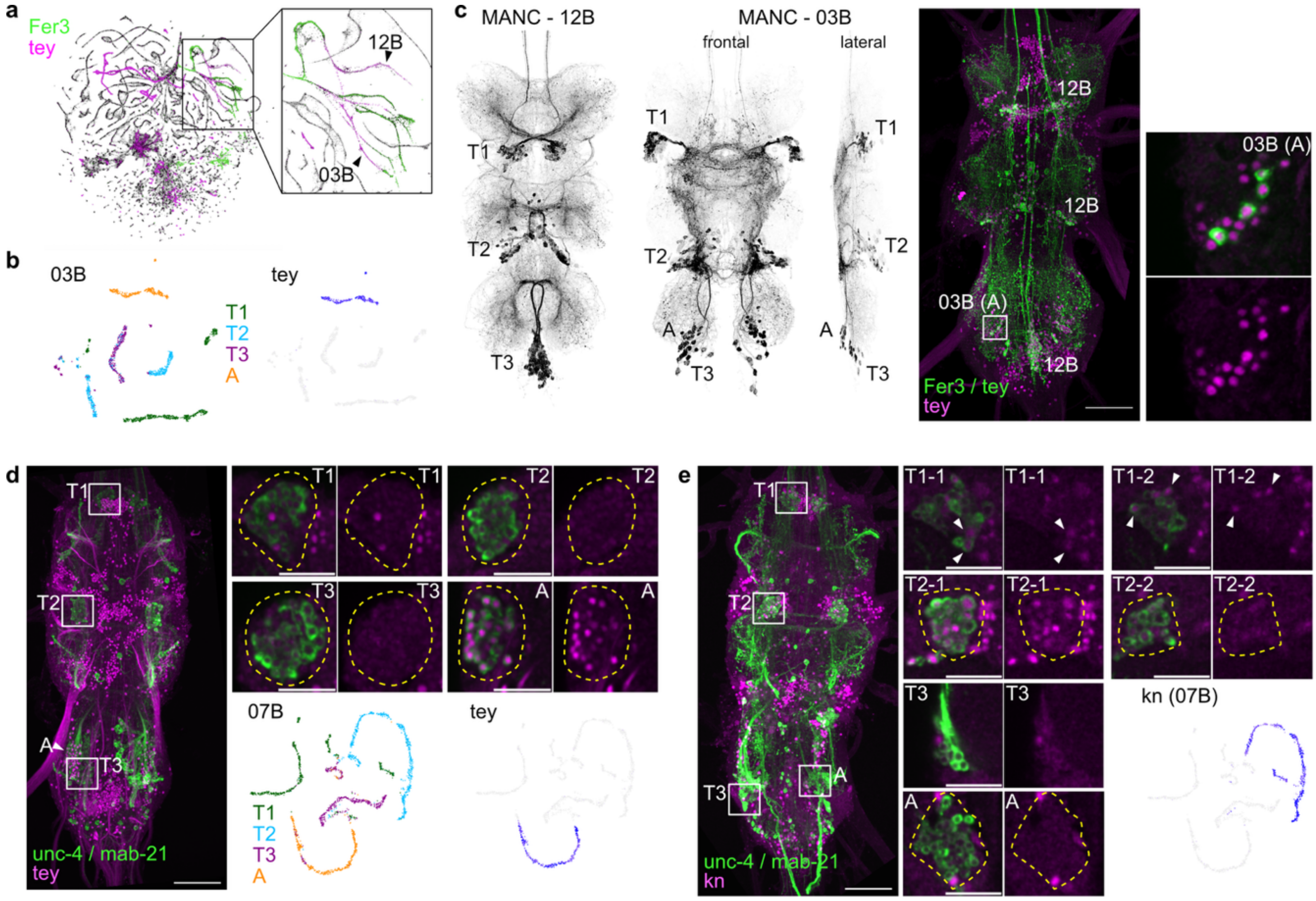

Supplementary Fig. 4 | Validation of segments annotation

a, UMAP showing co-expression of *Fer3* and *tey* in secondary hemilineages 12B and 03B. **b,** UMAPs showing 03B secondary neurons (48 h.a.p.f. only) upon re-clustering. Colour coding: segment annotation (left) and *tey* expression levels (right). Only abdominal neurons are predicted to be *tey*+. **c,** Greyscale: MANC neuronal meshes for 12B (left, frontal view) and 03B (middle, frontal and lateral views) secondary neurons. Right: maximum intensity projections of confocal stacks showing split-GAL4 intersection pattern of *Fer3* and *tey* (green) and tey protein (magenta). For 03B hemilineage, labelling is restricted to abdominal neurons which express tey. **d,** Split-GAL4 intersection of *unc-4* and *mab-21* (green), specifically labelling 07B hemilineage across segments, and tey protein (magenta). Arrowhead points to abdominal neurons that are posterior and therefore partially overlapping with T3 ones in the maximum intensity projections. Insets show 07B neuronal somas across segments, with *tey* expression restricted to the abdominal segment as predicted by the atlas. UMAPs show 07B secondary neurons (48h only) upon re-clustering. Colour coding: segment annotation (left) and *tey* expression levels (right). **e,** Split-GAL4 intersection of *unc-4* and *mab-21* (green) and kn protein (magenta). Insets show 07B neuronal somas across segments, with *kn* expression restricted to a subset of T1 neurons (arrowheads) and most T2 neurons, as predicted by the atlas (see UMAP for 07B). Insets show a single z-plane; in e two planes are shown for T1 and T2 to show *kn* expression heterogeneity. Scale bars: maximum intensity projections = 50 μm; insets: 20 μm. Images are representative of at least 3 stained individuals across 2 or more independent experiments.

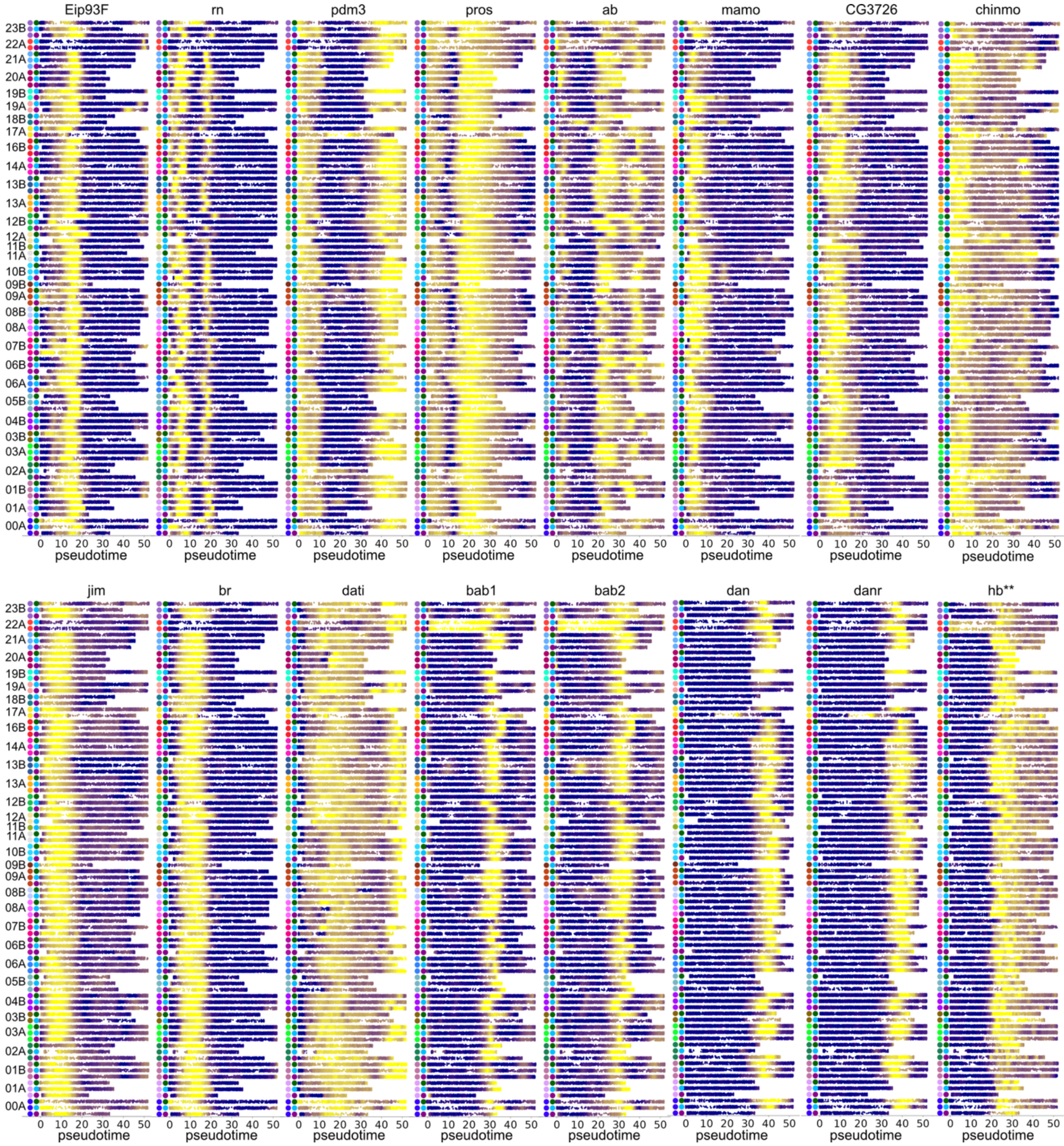

Supplementary Fig. 5 | Global patterning of shTFs expression across hemilineages

Normalised expression along aligned pseudotime of shTFs. Each row represents a hemilineage-segment trajectory. Colours on the left refer to hemilineage (left, refer to **Fig. 2b** for colour correspondence) and segment (right, refer to **Fig. 2f** for colour correspondence). Hb, the first temporal TF in the embryonic neuroblast temporal cascade, is also shown. Despite not emerging from our analysis of DEGs along trajectories, *hb* shows a consistent patterned expression across trajectories.

Supplementary References

118. Hartenstein, V. Morphological diversity and development of glia in Drosophila. *Glia* **59**, 1237–1252 (2011).

119. Coyne, R. *et al.* Regulatory logic of neuronal identity specification in Drosophila. *bioRxivorg* (2025) doi:10.1101/2025.09.01.673531.

120. Li, X. *et al.* Temporal patterning of Drosophila medulla neuroblasts controls neural fates. *Nature* **498**, 456–462 (2013).

121. Zeng, H. What is a cell type and how to define it? *Cell* **185**, 2739–2755 (2022).

122. Stanley, G., Gokce, O., Malenka, R. C., Südhof, T. C. & Quake, S. R. Continuous and discrete neuron types of the adult Murine striatum. *Neuron* **105**, 688–699.e8 (2020).
