# Supplementary material for: A global molecular code for birth order and neuronal identity in Drosophila
Source: Nature. 2026 Jul 22;657(8130):202–12. doi: 10.1038/s41586-026-10797-w (PMC13538039; doi:10.1038/s41586-026-10797-w)
Supplement: Supplementary file 2 [file 41586_2026_10797_MOESM2_ESM.pdf]

Corresponding author(s): Sebastian Cachero, Gregory S.X.E. Jefferis, Erika Donà

Last updated by author(s): May 18, 2026

Reporting Summary

Nature Portfolio wishes to improve the reproducibility of the work that we publish. This form provides structure for consistency and transparency in reporting. For further information on Nature Portfolio policies, see our Editorial Policies and the Editorial Policy Checklist.

Statistics

For all statistical analyses, confirm that the following items are present in the figure legend, table legend, main text, or Methods section.

n/a	Confirmed	
☐	☒	The exact sample size (*n*) for each experimental group/condition, given as a discrete number and unit of measurement
☐	☒	A statement on whether measurements were taken from distinct samples or whether the same sample was measured repeatedly
☐	☒	The statistical test(s) used AND whether they are one- or two-sided<br>*Only common tests should be described solely by name; describe more complex techniques in the Methods section.*
☒	☐	A description of all covariates tested
☐	☒	A description of any assumptions or corrections, such as tests of normality and adjustment for multiple comparisons
☐	☒	A full description of the statistical parameters including central tendency (e.g. means) or other basic estimates (e.g. regression coefficient) AND variation (e.g. standard deviation) or associated estimates of uncertainty (e.g. confidence intervals)
☐	☒	For null hypothesis testing, the test statistic (e.g. *F*, *t*, *r*) with confidence intervals, effect sizes, degrees of freedom and *P* value noted<br>*Give P values as exact values whenever suitable.*
☒	☐	For Bayesian analysis, information on the choice of priors and Markov chain Monte Carlo settings
☒	☐	For hierarchical and complex designs, identification of the appropriate level for tests and full reporting of outcomes
☒	☐	Estimates of effect sizes (e.g. Cohen's *d*, Pearson's *r*), indicating how they were calculated

Our web collection on statistics for biologists contains articles on many of the points above.

Software and code

Policy information about availability of computer code

Data collection

Single cell expression libraries were generated using ChromiumTM Next GEM Single Cell 3' HT v3.1. Sequencing was performed on a NovaSeq6000 machine (Illumina).
Confocal stacks were acquired using either Zeiss LSM 880 microscope with Airyscan operated by Zen10 software, Leica TCS SP8 3X gated STED, Leica SP5 Spectral Systems or Nikon ECLIPSE Ti2 confocal microscopes, depending on the experiment as specified in Methods.

Data analysis

Imaging and neuroanatomical data were processed using: Fiji (Open source, https://fiji.sc/, 1.54p); Ilastik (Open source, https://www.ilastik.org/, 1.4.1); VVD viewer (Open source, https://zenodo.org/records/14872850, 1.7.4); CMTK (Open source, https://www.nitrc.org/projects/cmtk/, 3.4.0); and Neuroglancer (Open source, https://github.com/google/neuroglancer/, commit 7681ed7).

scRNAseq data were pre-processed using: Cellranger (10x Genomics, https://www.10xgenomics.com/software, 8.0.0); Vireo (Huang et al. 2019, https://github.com/single-cell-genetics/vireo, 0.5.8); Demuxafy (Neavin et al. 2024, https://demultiplexing-doublet-detecting-docs.readthedocs.io/en/latest/index.html, 3.0.0); cellsnp-lite (1.2.3).

Python-based analyses were performed using: Python (Open source, https://www.python.org/, 3.12.8); G2G (Sumanaweera et al. 2024, https://github.com/Teichlab/Genes2Genes, commit 99c27d6); navis (https://zenodo.org/records/18801093, 1.10.0); numpy (Open source, https://numpy.org/, 1.24.2); and devCellPy (Galdos et al. 2022, https://github.com/devCellPy-Team/devCellPy, 1.5.0); G2G (Sumanaweera et al. 2024, https://github.com/Teichlab/Genes2Genes, commit 99c27d6); and devCellPy (Galdos et al. 2022, https://github.com/devCellPy-Team/devCellPy, 1.5.0).

R-based analyses were performed using R (Open source, https://www.r-project.org/, 4.4.1) and the following packages: Seurat (Hao et al. 2024, https://github.com/satijalab/seurat, 5.1.0); Monocle3 (Trapnell et al. 2014; Qiu et al. 2017; Cao et al. 2019, https://github.com/cole-

trapnell-lab/monocle3, 1.3.7); miloR (Dann et al. 2022, https://github.com/MarioniLab/miloR, 2.0.0); natverse (Bates et al. 2020, https://github.com/natverse, 0.2.4); tidyr (1.3.1); Matrix (1.7-2); tidyselect (1.2.1); lattice (0.22-6); magrittr (2.0.4); glue (1.8.0); stringr (1.5.1); tibble (3.2.1); dplyr (1.1.4); vctrs (0.6.5); purrr (1.0.2); pillar (1.10.2); rlang (1.1.6); stringi (1.8.7); ggplot2 (3.5.2); RColorBrewer (1.1-3); tidyverse (2.0.0); viridis (0.6.5); googlesheets4 (1.1.1); malevnc (0.3.1); neuprintr (1.3.2); fafbseg (0.15.3); fancr (0.4.0); pheatmap (1.0.12); patchwork (1.3.0); readr (2.1.5); Cairo (1.6-2); aricode (1.0.3); lsa (0.73.3); wesanderson (0.3.7); cluster (2.1.8); plotly (4.10.4); hdf5r (1.3.11); SCopeLoomR (0.13.0); FNN (1.1.4.1); igraph (2.1.4); scales (1.4.0); SeuratDisk (0.0.0.9021); SeuratWrappers (0.3.5); ggbeeswarm (0.7.2); cowplot (1.1.3); Rvcg (0.24); magick (2.8.7); ggrepel (0.9.6); ggpattern (1.1.4); nat (1.11.0); nat.nblast (1.6.7); dendextend (1.17.1); smoother (1.3); forcats (1.0.0); ggpmisc (0.6.1); broom (1.0.7); ggbreak (0.1.2); nat.templatebrains (1.2.1); ggpubr (0.6.0); dendroextras (0.2.3); coconat (0.1.2); googledrive (2.1.1); BiocParallel (1.38.0); scater (1.32.1); unix (1.5.9); reshape2 (1.4.4); BBmisc (1.13); future (1.34.0); SingleCellExperiment (1.26.0); scrattch.io (0.1.0); rtracklayer (1.64.0); splus2R (1.3-5); gridExtra (2.3); rgl (1.3.1); scran (1.32.0); scCustomize (3.0.1); and ConsRank (2.1.5).

Code availability:
Code with instructions to download and inspect data from GEO and SCope is reported in GitHub https://github.com/FlyNeuroAtlas/DevSeqVNC (commit 0e4827b or later)

For manuscripts utilizing custom algorithms or software that are central to the research but not yet described in published literature, software must be made available to editors and reviewers. We strongly encourage code deposition in a community repository (e.g. GitHub). See the Nature Portfolio guidelines for submitting code & software for further information.

Data

Policy information about availability of data

All manuscripts must include a data availability statement. This statement should provide the following information, where applicable:

- Accession codes, unique identifiers, or web links for publicly available datasets
- A description of any restrictions on data availability
- For clinical datasets or third party data, please ensure that the statement adheres to our policy

10x scRNA-seq data collected in this study (FASTQ files, cellranger outputs and processed and annotated Seurat objects) are available at Gene Expression Omnibus (GEO) under accession number GSE304221, and at BioProject under accession number PRJNA1297747. Annotated loom files are also accessible in Zenodo (full dataset: https://zenodo.org/records/17183028; neurons: https://zenodo.org/records/17184089, glia: https://zenodo.org/records/17185431) and in SCope (https://scope.aertslab.org/#/HundredDrills/*/welcome), an interactive web platform for exploring single-cell datasets. SCope interface allows rapid inspection of marker genes and cell-level information, making it straightforward to interact with the data. Code with instructions to download and inspect data from GEO and SCope is reported in GitHub https://github.com/FlyNeuroAtlas/DevSeqVNC. Data presented here, including links between the atlas and the connectome, can also be browsed on a dedicated webpage (https://flyem.mrc-lmb.cam.ac.uk/VNCatlas). Previously published 10x scRNA-seq data used in this study (Allen et al. 2020) are available at GEO under accession number GSE141807. Additional imaging data is available upon request.

Research involving human participants, their data, or biological material

Policy information about studies with human participants or human data. See also policy information about sex, gender (identity/presentation), and sexual orientation and race, ethnicity and racism.

Reporting on sex and gender	Does not apply.
Reporting on race, ethnicity, or other socially relevant groupings	Does not apply.
Population characteristics	Does not apply.
Recruitment	Does not apply.
Ethics oversight	Does not apply.

Note that full information on the approval of the study protocol must also be provided in the manuscript.

Field-specific reporting

Please select the one below that is the best fit for your research. If you are not sure, read the appropriate sections before making your selection.

☒ Life sciences ☐ Behavioural & social sciences ☐ Ecological, evolutionary & environmental sciences

For a reference copy of the document with all sections, see nature.com/documents/nr-reporting-summary-flat.pdf

Life sciences study design

All studies must disclose on these points even when the disclosure is negative.

Sample size	For a connectomic neuron type to be identified as a distinct cluster in scRNA-seq we needed a minimum coverage of 20-30 (see Manuscript Introduction). Since the number of neurons reported in MANC is 7880, distributed across ~3.5k types, we aimed to collect at least 200k

neurons. With a cell recovery of ~24k cell singlets/10x lane, we aimed to collect 24 lanes (~600k cells) to ensure the desired number of cells upon quality control and exclusion of glial cells to achieve >30x coverage across developmental stages.

Data exclusions

Sample SITTD8 was excluded from further analysis because, unlike all other samples which showed a bimodal distribution of UMIs and features across cells, SITTD8 displayed a unimodal distribution, indicating it was an outlier.
To ensure high-quality data, several filtering steps were applied at both the cell and gene level. First, cells were excluded if they contained fewer than 700 detected genes, more than 20% of transcripts mapping to mitochondrial genes, or more than 50% mapping to ribosomal genes, all standard thresholds for scRNAseq data analysis. Following data integration, additional thresholds were applied to remove cells with fewer than 3,000 unique molecular identifiers (UMIs), as well as entire clusters with an average UMI count below this value, as these were considered low-quality.

At the gene level, any gene expressed in fewer than three cells across the entire dataset was excluded from further analysis, ensuring that only genes with meaningful expression patterns were retained.

Finally, to eliminate doublets—instances where a single barcode captures transcripts from more than one cell—demultiplexing results from the Vireo package were used. Cells showing a high proportion of SNPs from multiple DGRP lines were classified as doublets and removed from the dataset.

Replication

scRNA-seq libraries were generated across three independent collection days. All replicates were successful and only cells from SITTD8 (see Data Exclusion) were excluded from further analysis. On each day, four separate experiments were performed, each including eight samples. In total, data were collected from 92 individual animals, covering four developmental stages and both sexes. Each stage–sex condition is represented by 5 to 18 biological replicates, covering between 5 to 11 different genotypes. Some experimental preparations were used to create 2 or 3 independent libraries.

Randomization

Randomised samples from different genotypes were used for the same sex and developmental stages across experiments.

Blinding

No formal experimental blinding was applied for the generation and analysis of the scRNAseq atlas; however, computational analyses were performed in an unsupervised manner, and clustering and dimensionality reduction were carried out without using sample identity. Quality-control thresholds were applied uniformly across all samples.
EdU experiments were analysed following an automated pipeline, independent from sample identity.

Reporting for specific materials, systems and methods

We require information from authors about some types of materials, experimental systems and methods used in many studies. Here, indicate whether each material, system or method listed is relevant to your study. If you are not sure if a list item applies to your research, read the appropriate section before selecting a response.

Materials & experimental systems

n/a	Involved in the study	
☐	☒	Antibodies
☒	☐	Eukaryotic cell lines
☒	☐	Palaeontology and archaeology
☐	☒	Animals and other organisms
☒	☐	Clinical data
☒	☐	Dual use research of concern
☒	☐	Plants

Methods

n/a	Involved in the study	
☒	☐	ChIP-seq
☒	☐	Flow cytometry
☒	☐	MRI-based neuroimaging

Antibodies

Antibodies used

Commercially available primary antibodies were:
Mouse anti-brp monoclonal antibody (Developmental Studies Hybridoma Bank, DSHB; AB_2314866; clone nc82);
Chicken anti-GFP polyclonal antibody (Abcam; ab13970);
Chicken anti-GFP polyclonal antibody (Thermo Fisher Scientific; A10262);
Rabbit anti-GFP polyclonal antibody (Thermo Fisher Scientific; A11122);
Mouse anti-Neurotactin monoclonal antibody (DSHB; AB_528404, clone BP106);
Mouse anti-Antennapedia (Antp) monoclonal antibody (DSHB; AB_528083; clone 8C11);
Rat anti-mCD8 monoclonal antibody (Thermo Fisher Scientific, MCD0800, clone 5H10);
Mouse anti-Br-core monoclonal antibody (DSHB; AB_528104, clone 25E9.D7).

Additional primary antibodies generated in academic laboratories and not commercially available were:
Rabbit anti-Ubx antibody (polyclonal; originally described in Marin et al., 2012);
Rabbit anti-tey polyclonal antibody (gift from Angelike Stathopoulos; described in Macabenta & Stathopoulos 2019);
Guinea pig anti-kn polyclonal antibody (gift from Walton Moore; first described in Jinushi-Nakao, S. et al. 2007, DOI: 10.1016/j.neuron.2007.10.031);
Guinea pig anti-hth polyclonal antibody (gift from Nikos Konstantinides; described in Özel et al. 2021);
Rabbit anti-Bab1 polyclonal antibody (gift from Muriel Boube; described in Bourbon et al. 2022);
Rat anti-dan polyclonal antibody (gift from Claude Desplan; generated by GenScript against FBgn0039286 amino acids 8–87);
Rabbit anti-Eip93F polyclonal antibody (gift from Daniel McKay; described in Uyehara et al. 2017);
Guinea pig anti-mamo polyclonal antibody (gift from Claude Desplan; described in Rossi & Desplan 2020);

Rat anti-Bab2 polyclonal antibody (gift from Muriel Boube; described in Bourbon et al. 2022);
Rat anti-Pdm3 polyclonal antibody (gift from Claude Desplan; described in Zhang et al. 2023).

Secondary antibodies used were:
Goat anti-mouse IgG Alexa Fluor 568 (Thermo Fisher Scientific; A11001);
Goat anti-mouse IgG1 Alexa Fluor 594 (Thermo Fisher Scientific; A21125);
Goat anti-mouse IgG1 Alexa Fluor 647 (Thermo Fisher Scientific; A21240; lot n. 1563685);
Goat anti-rat IgG Alexa Fluor 555 (Thermo Fisher Scientific; A21434);
Goat anti-rat IgG Alexa Fluor 647 (Thermo Fisher Scientific; A21247; lot n. 714287);
Goat anti-chicken IgY Alexa Fluor 488 (Thermo Fisher Scientific; A11039; lot n. 488734);
Goat anti-rabbit IgG Alexa Fluor 488 (Thermo Fisher Scientific; A11034; lot n. 54533A);
Goat anti-rabbit IgG Alexa Fluor 568 (Thermo Fisher Scientific; A11036; lot n. 757102 or 1504529);
Goat anti-rabbit IgG Alexa Fluor 594 (Thermo Fisher Scientific; A32740; lot n. 3214333);
Goat anti-rabbit IgG Alexa Fluor 633 (Thermo Fisher Scientific; A21070; lot n. 751102);
Goat anti-guinea pig IgG Alexa Fluor 555 (Thermo Fisher Scientific; A21435);
Goat anti-guinea pig IgG Alexa Fluor 647 (Thermo Fisher Scientific; A21450);
Donkey anti-rat IgG Alexa Fluor 488 (Thermo Fisher Scientific; A21208);
Donkey anti-mouse IgG Alexa Fluor 555 (Thermo Fisher Scientific; A31570);
Donkey anti-rabbit IgG Alexa Fluor 647 (Thermo Fisher Scientific; A31573)

Validation

All primary antibodies used in this study were validated based on a combination of supplier information, previously published characterization, and consistency with known expression patterns.
Commercial antibodies (including mouse anti-brp, anti-Neurotactin, anti-Antp, and anti-Br-core from DSHB; anti-GFP antibodies from Abcam and Thermo Fisher; and rat anti-mCD8) have been validated by the manufacturers and are widely used in the field. These antibodies produced reproducible staining patterns consistent with established expression domains. In particular, anti-Brp (nc82) labeled synaptic neuropil structures as expected, anti-Neurotactin and anti-Antp showed established spatial expression patterns, and anti-GFP antibodies produced signal exclusively in GFP-expressing samples, with no detectable signal in negative controls lacking GFP.
The rabbit anti-Ubx antibody and all custom antibodies generated in academic laboratories (anti-tey, anti-kn, anti-hth, anti-Bab1, anti-dan, anti-Eip93F, anti-mamo, anti-Bab2, and anti-Pdm3) were previously validated in the original publications cited, where specificity was demonstrated through approaches such as agreement with known gene expression patterns and, in several cases, loss-of-function or genetic perturbation analyses. In the present study, staining obtained with these antibodies was consistent with reported expression domains and expected biological patterns.
Together, these independent lines of evidence support the specificity and reliability of all antibodies used.

Animals and other research organisms

Policy information about studies involving animals; ARRIVE guidelines recommended for reporting animal research, and Sex and Gender in Research

Laboratory animals

All experiments were performed on Drosophila melanogaster male and female animals as stated in the manuscript. Several developmental stages were used, depending on the experiment: L3 larvae for Hox protein stainings; 6, 24, 36, 48hAPF for the generation of the transcriptional atlas; up to 10 days after eclosure adults for additional immunostainings. Experiment-specific developmental stages are indicated in the manuscript. Flies were raised on standard cornmeal medium at 25°C under a 12h light/dark cycle.

Wild animals

No wild animals were used in this study.

Reporting on sex

Samples from both sexes were used to generate the atlas in similar number. We carefully annotated the sex of the samples employed in the study and report it in the atlas meta data and in figures when applicable.

Field-collected samples

No field collected samples were used in this study.

Ethics oversight

According to institutional and national guidelines, invertebrate research organisms like Drosophila do not require formal ethical approval.

Note that full information on the approval of the study protocol must also be provided in the manuscript.

Plants

Seed stocks

Does not apply.

Novel plant genotypes

Does not apply.

Authentication

Does not apply.
