# Supplementary material for: A global molecular code for birth order and neuronal identity in Drosophila
Source: Nature. 2026 Jul 22;657(8130):202–12. doi: 10.1038/s41586-026-10797-w (PMC13538039; doi:10.1038/s41586-026-10797-w)
Supplement: Supplementary file 3 [file 41586_2026_10797_MOESM4_ESM.pdf]

nature portfolio

Peer Review File

A global molecular code for birth order and neuronal identity in Drosophila

Corresponding Authors: Dr Sebastian Cachero, Dr Erika Donà, Dr Gregory Jefferis

Any redactions in this file are there to maintain patient confidentiality, the confidentiality of unpublished data, or to remove third-party material.

This file contains all reviewer reports in order by version, followed by all author rebuttals in order by version.

Attachments originally included by the reviewers as part of their assessment can be found at the end of this file.

Version 0:

Reviewer comments:

Referee #1

(Remarks to the Author)
Summary of key results and significance:
The generation of neural diversity remains a central problem in developmental neurobiology. The manuscript presents a high-resolution transcriptional atlas for the Drosophila ventral nerve cord (VNC) from 4 developmental stages during pupal development. By pairing this developmental scRNA-seq atlas with the complete connectome, the authors link molecular identity, developmental lineage, and circuit architecture. The atlas reached ~38× coverage—sufficient in principle to capture even single-cell EM types. The authors mapped the embryonic-born primary and larval-born secondary neurons, and found that interestingly, primary neurons form discrete clusters, while secondary neurons form continuous trajectories for each hemilineage. Next, they annotated hemilineage and segment identities. Further analysis identified a "global temporal code" of 17 transcription factors (TFs) expressed in a conserved order along pseudotime and across hemilineages. They also analyzed sexual dimorphism shaped by apoptosis and transcriptional divergence.

Overall assessment:
The dataset presented is of impressive quality and coverage, and represents a highly valuable resource for study of neural development in Drosophila ventral nerve cord. The developmental principles illustrated are highly interesting, especially a global temporal code of transcription factors in postmitotic neurons is very attractive. However, several core claims—particularly those concerning segment annotation, the consistency of the expression patterns of 17 TFs across hemilineages, and the causal interpretation of the 17-TF sequence as a birth-order code defining neural diversity—require substantial additional analysis and/or validation.

Major Comments:
1. Verification of the segment annotation. For a large majority of neurons, segment identity was predicted using threshold-based approach. Experimental confirmation of the differential expression levels of Hox genes within hemilineages across segments would validate the threshold-based annotation. Alternatively, differential expression analysis within a hemilineage across segments could identify differentially expressed markers in different segments, and experimental verification of a representative subset would validate the threshold-based annotation.
2. Alignment robustness. The nonlinear alignment presumes a global temporal axis across hemilineages. This is reasonable but should exclude over-registration artifacts by using alternative reference trajectories.
3. Inconsistency in the expression patterns of the 17 TFs shown in different figures. The expression patterns of shTFs, such as Eip93F, Pdm3, Pros, Mamo, ab, CG3726, br, bab1/2, dan/danr shown in Fig 4g heatmap is strikingly different from those shown in Extended Data Fig. 9. For example, Mamo is shown as expressed around psedotime 40 (late-born neurons) in Fig 4g, but expressed in psedotime 0-10 (early-born neurons) in all hemilineages in Extended Data Fig. 9. In addition, in Fig 4g, the y-axis of the bottom panel appears misaligned; and heatmap on the top appears not consistent with the peak map on the bottom, for example: CG3726 is expressed at a late pesudotime in the 03AT1 heatmap, but is shown as an early "active peak" in the bottom; Dan is expressed at very early pesudotime in the upper heatmap but the active peak is placed at a late pseudotime in the lower panel. These discrepancies undermine confidence in the shared-pseudotime alignment, and raise

doubts on the global temporal code hypothesis.
4. Verification and functional significance of the “global temporal code.” Experimental verification of the protein expression patterns of at least some of these proposed shTFs in neurons born at different times will greatly strengthen the conclusion. Also, there’s no direct perturbation showing that specific shTFs instruct fate or maintain identity. To functionally validate the temporal code, perturb at least one early and one late shTF (or a small combo) in a chosen hemilineage; and show predictable shifts in neuronal identity (molecular markers, morphology, and/or EM type match).

Minor Comments
1. Figure 1C color palette.
The current palette uses similar hues across stages, especially after regression, making it hard to distinguish timepoints on the UMAP. Use a color-blind-safe palette with maximally separated hues and consistent legends across panels. This will help readers to visualize where the cells of different pupal stages are located in the trajectory, and accurately assess if there is any contribution of maturation to the expression patterns of shTFs.
2. Extended Data Fig. 6b: stage clarity. Explicitly label the developmental stage(s).
3. Provide a Data & Code table (accessions, commit hashes, environment) to maximize reuse of the resource.
4. Line 986, Method part, “Hemilineage identity was then further confirmed by published split lines”. Please include a detailed description for which hemilineages, identities have been confirmed using this method and the split lines used.
5. Line 1020- 1024, “In cases where immunostaining data from L3 larvae indicated that expression of Antp and Ubx genes deviated from the canonical model, we used these data as guidance”. Please list all hemilineages for which these data are available, in addition to those shown in Extended Data Fig. 6.

Referee #2

(Remarks to the Author)
I co-reviewed this manuscript with one of the reviewers who provided the listed reports.

Referee #3

(Remarks to the Author)
How neuronal diversity is generated in the cell lineages of neural stem cells is an essential question in neural development. This paper addresses this question using the ventral nerve cord of Drosophila, generating a large single-cell atlas of neurons at several stages of development. There are several conclusions:

- Neurons that are generated sequentially express different transcription factors almost independently from where the stem cell originated within a segment or which segment they come from. This argument of time-dependent TFs has been made several times in other papers as early as 2011 (e.g. ‘concentric genes’ in DOI: 10.1242/dev.058370) and in more recent publications on the visual system and in the central brain (a very similar paper posted for months in bioRxiv on central brain lineages, doi: 10.1101/2025.06.03.657692).

- A major point made in the abstract is that the number of neurons identified by the connectome grossly outnumbers the number of cell types defined by transcriptomics. However, this argument is based on totally outdated papers (2018! ref 24-26) and the argument is not valid as in-depth developmental atlases show close (but not perfect) matches to the connectome. This is discussed below (and should be discussed in the paper!).

- Another important argument is that larval born neurons show a continuum of cell fates while embryonic born neuron types are more discreet. This argument has been made in mammals and is most likely due to insufficient depth of sequencing (in spite of the 38X coverage, see below).

- The paper also explores sex-dimorphic neurons and shows that male and female neurons can have different fates, including death. This has been documented in the past and in the recent bioRxiv paper above. This is an add-on to the paper that is not necessary.

In general, one major issue with the paper is that the authors appear to not be aware of, or to ignore results from several groups that have done similar developmental transcriptomic analyses on the visual or olfactory systems (papers cited but not discussed appropriately) that present a much more favorable situation because neurons of each type are present multiple times. The bioRxiv paper above makes very similar points and should also be referenced. These papers made similar points, or contradict some of the most important (related) statements made here, e.g. continuum of cell types, and discrepancy between transcriptome and connectome.

Evaluation:

- One strength of the paper is the 38x depth of scRNAseq, with several hundred thousand neurons. This is necessary since each neuron is represented only once or a few times bilaterally in each organism, which makes it difficult to cluster efficiently. The data are of quality. However, the 38x coverage includes all stages merged together. This is problematic as the same resolution cannot be attained early on (P6), no matter the number of cells because newborn neurons are much harder to tell apart from each other, especially if they are developmentally related (diversity will increase at the time of synaptogenesis). This is supported by the fact that the authors do not observe the continuum for mature primary neurons.

It is suggested to re-do the clustering analysis using only the late, or P36+48 datasets (when all neurons had enough time to differentiate from their terminal division) and the continuum might disappear, although now the coverage at these stages might not be sufficient to resolve individual cell types. In general, one should be much more careful about overinterpreting UMAPs and their depth, which is commonly done in mammalian data that are almost always underpowered.

- A significant result is the distinction between early embryonic mature neurons and the late larval neurons born immature neurons formed after the neural stem cells (neuroblasts) have been reactivated.

- The authors then conclude from this separation that embryonic born neurons are discreetly specified while larval born neurons form continuous trajectories. However, the demonstration of this continuum, which would reflect the fact that there is no transcriptional distinction among neurons, is not clear. The same argument of a continuum has been made for scRNAseq of human brain neurons where clusters are most often poorly separated due to insufficient depth (e.g. 3,000 neuron types in the mouse brain, which is almost as many neurons as have been defined in current transcriptomics in flies!). The fact that there are more cell types in mice than in humans illustrates this as well. Furthermore, neuronal specification in Drosophila is fairly rigid, with little reported examples of plasticity. Thus, the continuum likely reflects the fact that even 38X (with the caveat mentioned above about focusing only on late time points) is not enough to separate closely related neurons and the authors should be very careful making the argument of a continuum as this will likely be dismissed when deeper sequencing is possible.

- The authors argue that adult scRNAseq grossly underestimates cell types because genes encoding connections between neurons are no longer represented in the adult. This is correct and this argument has been made in several developmental atlases on other regions of the brain, olfactory system, visual system and central brain. If these papers show that there is indeed more diversity early, this cannot account for the huge discrepancy between connectome and transcriptome observed in the current paper. The discrepancy in sensory systems is minimal because each neuron type is present multiple times in the brain and can be clustered and subclustered much more precisely to match neurons from the connectome. Therefore, if a few identical neurons can acquire different morphologies either through stochastic processes (e.g. DCN neurons) or by connecting to different neurons, this is not enough to explain the difference with the connectome observed here. A recent paper on the fish retina also made the same argument that functional cell types cannot be distinguished by their transcriptomes (doi: 10.1038/s41586-024-08518-2) but it is likely due to insufficient coverage and the late time when scRNAseq was performed. Thus, as argued above, even the many neurons in the current paper are likely not be enough to cluster related neurons.

- The manuscript also confirms that the last NotchON/NotchOFF division of GMCs greatly affects the outcome of the lineages and that one should only consider hemilineages. The notion of hemilineages is old, going back to earlier MARCM studies and it has been made in earlier studies that showed that Notch can distinguish between excitatory (on) and inhibitory (off) neurotransmitters (see also the bioRxiv paper referred to above).

- The paper defines a series of transcription factors that reflect birth order. The concept of terminal selectors that correlate with birth order regardless of lineage is not a novel concept. (see concentric TFs in the visual system). As mentioned above, the authors avoid any discussion of optic lobe findings that would be important for their arguments. There are always exceptions, because the expression of the same TF can easily be expressed independently in different lineages (see Fig 4g). However, if one should be careful suggesting that a given TF is a perfect marker of a temporal window across an entire brain region, the argument is of interest. The authors should compare their temporal terminal selector TFs with those in the bioRxiv paper mentioned above and with neurons in sensory systems.

Minor points:

- It is surprising that only 6 glial types are identified. In other brain regions many more numerous glial types have been identified.

- When it comes to serial lineages, the presence of a dominant Hox gene is in strong support of the position along the rostro-caudal part of the axis. The authors appear concerned by the overlap between Hox genes which is not an issue due to posterior dominance.

- One potential caveat is that clusters might not represent pure hemilineages as neurons from hemilineage might jump clusters and become more similar to neurons from another one. The authors are careful about this and include Gal4 lines labeling specific lineages in the sequencing. However, in Fig. 2c, sections of the labeled trajectories are missing the label, which do not look random (e.g. due to GFP dropout). Depending how clean and complete those Gal4 lines are, this points to evidence that hemilineages do mix in their “trajectories”. Most lineage-related neurons remain similar to each other because all seem to have a “main” cluster where most of the label is, but it is clearly not that neat.

In conclusion, this is an interesting atlas of the ventral nerve cord but several of the conclusions should be strongly dimmed or eliminated since these arguments have already proven to not be correct in other, much easier systems. The authors should be much more scholarly and look beyond the nerve cord or even the central brain and discuss data from the visual or olfactory systems that are not consistent with their own arguments, simply because even current brute force is not enough to go deep enough in the transcriptome of very sparse neurons.

Referee #4

(Remarks to the Author)
"A Developmental Atlas of the Drosophila Nerve Cord Uncovers a Global Temporal Code for Neuronal Identity" by Cachero and colleagues presents a transcriptional atlas of the developing adult Drosophila nerve cord, leveraging scRNA-seq from four pupal time points. The authors provide a comprehensive annotation of neuronal populations developmental stages. They link transcriptional identities to morphological and functional characteristics, investigate sexual dimorphism, and provide contextual insights into the general principles of neural development. This study is a significant advance in the field, providing high-resolution characterization of multiple developmental features (birth time, hemilineage, birth order, sex). Uniting transcriptomic, developmental, and connectomic variation at scale offers novel insights into neurodevelopmental mechanisms. The manuscript identifies generalizable principles in neural development and circuit assembly. The authors unite single-cell transcriptomics and connectomics to address the longstanding challenge of linking molecular identity to circuit architecture across the nervous system. This resource will be valuable for both developmental and systems neuroscience.
Sequencing across developmental stages allows the authors to interrogate the role of temporal patterning, hemilineage, and segmental regionalization in neurodevelopment. Dimensional reduction (UMAP) convincingly segregates primary (embryonic) and secondary (larval) neurons in transcriptomic space. Manual annotation using established molecular markers is appropriate, and the clear segregation of neuronal clusters corroborates previous findings. Integration of lineage information from connectomics and molecular markers is an asset that facilitated automated neuronal classification. The authors identify transcription factor combinations for hemilineage classification, with nearly half identifiable by a single marker. These markers allow for classification across developmental time, overcoming annotation challenges for immature neurons. The use of Hox transcription factors to assign segment identities is convincing and supported by developmental and anatomical context. The finding that serial homology is evident in transcriptomic and connectomic data is novel and provides mechanistic insight to neuronal morphology. Pseudotime calculation for each hemilineage reveals temporal transcription factor dynamics, which are clearly presented. Identification of sustained temporal transcription factor suggests terminal selector function, an important distinction from the role of temporal transcription factors in embryonic neurogenesis. Because the temporal transcription factor code is shared with the developing brain, the findings herein likely highlight general principles of neurodevelopment.
Additionally, the authors investigated sexual dimorphism in the nerve cord, identifying differentially abundant populations which are validated by sex-determination gene expression. They find that this dimorphism is biased by neuronal birth time such that male enriched neurons are mostly secondary neurons while female enriched are mostly primaries. Transcriptional dimorphisms align with knowns from connectomic data, and their atlas can be used to confidently predict dimorphisms. The authors find that female-specific apoptosis underscores dimorphism to some extent. Furthermore, differential guidance cue receptor expression between sexes is suggested to drive morphological dimorphism, a significant contribution to linking molecular and morphological features between transcriptomes and connectomes, respectively.
Emphasis on developmental timing and predictive modeling is well supported. Methods are well-suited and appropriately chosen. Data quality and presentation are high. Statistical treatment and assessment of uncertainties are sufficient.
The conclusions are strongly supported by the methodology. The multi-level annotation, careful consideration of segmental identity genes and sex, and integration with connectomic data all substantiate the authors' claims. The manuscript is overall clearly written and provides ample methodological and scientific context. The abstract, introduction, and conclusions frame the findings well for both expert and non-expert readers.

Suggested Improvements
• Clarify terminology such as “VNC hemisphere” (e.g., line 11) and check for spelling consistency (organization/organisation).
• The discussion around lines 66–69 is confusing and could be more explict.
• Explicitly differentiate "birth time" (embryo/larva) from "birth order" within lineages in discussion (lines 87–98)—this may be confusing for readers unfamiliar with insect neurodevelopment.
• Add citation for lines 89–90 as appropriate.
• The claim of "38x coverage of the half-VNC" may be slightly overstated, as sequencing was performed over 4 developmental stages rather than on mature tissue.

This work is a valuable contribution to neurodevelopmental biology and Drosophila connectomics. With minor revisions for clarity and citation, it will be suitable for publication.

Referee #5

(Remarks to the Author)
I co-reviewed this manuscript with one of the reviewers who provided the listed reports.

Version 1:

Reviewer comments:

Referee #1

(Remarks to the Author)
The revised manuscript has addressed all of our comments satisfactorily. Therefore, we support its publication on Nature. A

minor typo was noticed in line 335: "As predicted, while in hemilineage 12A...", but in Extended Data Fig. 8, it says "12B".

Referee #2

(Remarks to the Author)
I co-reviewed this manuscript with one of the reviewers who provided the listed reports.

Referee #3

(Remarks to the Author)
First of all, this review process induced very interesting discussions that will be interesting to pursue in the future when the paper is published!
This reviewer had a long lists of comments and some serious disagreements on two major points.
The authors have responded adequately to many of the points and even if not perfect, their arguments hold well
The two major points that needed to be absolutely addressed concerned:
- The fact that secondary neurons form a continuum. The authors have corrected this statement that seemed to indicate that these neurons were not different transcriptionally. They now use the term "gradual" that is not really different from continuum; however, the authors now explain that what they meant is the later born neurons were more closely related to each other than the early neurons. However, contrasting "discreet' with "gradual' might be mis-intepreted by the readers since, if the reviewer read the paper corrected, the authors do believe that later born neurons are also discreet while at the current resolution, too close to each other to be separated. They should emphasize this even more in the abstract and text.
- The second point had to do with the huge discrepancy between the number of connectome neurons and those from the transcriptome, which again gave the impression that the many neurons that differ by connectivity or morphology share the same transcriptome. However, the authors now clearly state that this is not the case and that much deeper transcriptome will distinguish among connectome-based neuron types.
- This reviewer is a little surprised by the statement about the lack of commonality among NotchON or OFF neurons while, in other systems, Notch is by far the strongest determinant of neural diversity. But the authors argue strongly against this and they might be right for the VNC!

In conclusion, this is an important paper that will bring light to a major issue of neural diversity that is completely not understood in vertebrates.
This reviewer hopes that the temporal series of TFs in post-mitotic neurons will soon be linked to a series of temporal factors in neuroblasts, a concept that was first elegantly articulated 27-years ago (1999!).

Referee #4

(Remarks to the Author)
The authors have addressed my major concerns. Thank you.

Referee #5

(Remarks to the Author)
I co-reviewed this manuscript with one of the reviewers who provided the listed reports.

Open Access This Peer Review File is licensed under a Creative Commons Attribution 4.0 International License, which permits use, sharing, adaptation, distribution and reproduction in any medium or format, as long as you give appropriate credit to the original author(s) and the source, provide a link to the Creative Commons license, and indicate if changes were made.
In cases where reviewers are anonymous, credit should be given to 'Anonymous Referee' and the source.
The images or other third party material in this Peer Review File are included in the article's Creative Commons license, unless indicated otherwise in a credit line to the material. If material is not included in the article's Creative Commons license and your intended use is not permitted by statutory regulation or exceeds the permitted use, you will need to obtain permission directly from the copyright holder.
To view a copy of this license, visit https://creativecommons.org/licenses/by/4.0/

Response to reviewers' comments for the manuscript: "A Developmental Atlas of the Drosophila Nerve Cord Uncovers a Global Temporal Code for Neuronal Identity"

We thank all reviewers for their careful evaluation of our work. Their comments were extremely valuable and helped us to gather additional evidence that, we trust the reviewers will now agree, strengthens our manuscript and conclusions. We have aimed to address all points raised by adding new experiments, analyses, and clarifications where needed. We hope that the revised manuscript and our responses fully address the concerns raised.

Please note that line numbers in our responses refer to those found in the manuscript with changes highlighted (manuscript_revised_track_changesON.pdf). In case the reviewers prefer a cleaner version, we also provide a manuscript_revised_track_changesOFF.pdf manuscript, but in that case the line numbers do not match our responses.

Referee #1:

Summary of key results and significance:

The generation of neural diversity remains a central problem in developmental neurobiology. The manuscript presents a high-resolution transcriptional atlas for the Drosophila ventral nerve cord (VNC) from 4 developmental stages during pupal development. By pairing this developmental scRNA-seq atlas with the complete connectome, the authors link molecular identity, developmental lineage, and circuit architecture. The atlas reached ~38× coverage—sufficient in principle to capture even single-cell EM types. The authors mapped the embryonic-born primary and larval-born secondary neurons, and found that interestingly, primary neurons form discrete clusters, while secondary neurons form continuous trajectories for each hemilineage. Next, they annotated hemilineage and segment identities. Further analysis identified a "global temporal code" of 17 transcription factors (TFs) expressed in a conserved order along pseudotime and across hemilineages. They also analyzed sexual dimorphism shaped by apoptosis and transcriptional divergence.

Overall assessment:

The dataset presented is of impressive quality and coverage, and represents a highly valuable resource for study of neural development in Drosophila ventral nerve cord. The developmental principles illustrated are highly interesting, especially a global temporal code of transcription factors in postmitotic neurons is very attractive. However, several core claims—particularly those concerning segment annotation, the consistency of the expression patterns of 17 TFs across hemilineages, and the causal interpretation of the 17-TF sequence as a birth-order code defining neural diversity—require substantial additional analysis and/or validation.
As detailed in point 1.3, we would like to apologise for the mistake in the labels of the panel in Fig4.g, which we think gave the impression of unreliable shTF expression.

Major Comments:

1.1 Verification of the segment annotation. For a large majority of neurons, segment identity was predicted using threshold-based approach. Experimental confirmation of the differential expression levels of Hox genes within hemilineages across segments would validate the threshold-based annotation.
Alternatively, differential expression analysis within a hemilineage across segments could identify differentially expressed markers in different segments, and experimental verification of a representative subset would validate the threshold-based annotation.
The choice of thresholds for segment annotation was based on a large collection of Ab stainings for the Hox genes Antp, Ubx and Abd-A performed in late L3 larvae upon neuroblast clones induction

using MARCM, of which we report some examples in **Extended Data Fig 6b**. We added a column to **Supplemental File 4** that summarises the observed expression levels across many images. This annotation, despite not being quantitative, is based on the comparative staining across hundreds of images, with qualitative scores assigned based on the range of intensity within each specimen/confocal stack. If the reviewer finds it necessary we could deposit such stacks on Zenodo, but it would take a highly specialised person to map the MARCM clones in the images to the correct lineage and we therefore think that sharing these data would not be immediately useful to the community. The data will nevertheless remain available upon request.
For several lineages, Ab stainings in L3 revealed Antp expression across all segments, but no Ubx, which could instead be detected at the RNA level in our pupal dataset, although often at low levels. We hypothesise that difference between the antibody and RNA datasets is likely due to the different developmental stages of the datasets, and therefore decided to assign Ubx-expressing cells in these hemilineages to T3, conforming the canonical view on VNC Hox gene expression. One such example is hemilineage 04B. Here we provide evidence that our segment annotation led to a verifiable hypothesis: while fru-MARCM experiments had only recovered a clone of fru+ cells in T1 (Cachero et al 2010), our segments annotation predicted fru+ cells in T1, T2 and T3. This was validated experimentally as shown in **Fig.6i**.
We have now performed additional prediction-validation experiments - detailed in **Validation of segments annotation** (methods) and described in **Extended Data Fig. 8** **[lines 334-341]**:
1- Our segment annotation predicts that the intersection of Fer3 and tey should label a subset of 12B cells in T1, T2 and T3, but only abdominal 03B cells. This is consistent with the expression pattern observed in Fer3/tey split-GAL4 intersection.
2- Antibody staining against tey confirms the prediction that this TFs is restricted to abdominal cells in 07B hemilineage. Our segment annotation would predict a very small subset of T3 cells to express tey, which we can't detect. As these cells fall on a branch of 07B that is otherwise containing only abdominal cells, it is possible that this constitutes a case of segment mis-annotation **[lines 338-341]**.
3- Antibody staining against kn confirms the prediction that this TF in 07B is restricted to a small fraction of T1 cells and a large fraction of T2 cells, while it is absent from other segments.
These new experiments support the overall quality of our segments annotation. However, since neurons belonging to hemilineages where Antp is expressed in all segments are arranged along a single trajectory, suggesting high homology across segments, it is very difficult to find segment-specific markers and thus to be fully confident about the annotation. We remark this limit in the text, stating that annotation confidence for low and medium complexity hemilineages is lower compared to complex ones.

1.2. Alignment robustness. The nonlinear alignment presumes a global temporal axis across hemilineages. This is reasonable but should exclude over-registration artifacts by using alternative reference trajectories.
The choice to pursue a nonlinear alignment strategy based on gene expression similarities was driven by qualitative observations that the 8 TFs we identified to be differentially expressed along trajectories in all hemilineages share the same ordering but occupy different trajectory lengths in both UMAP embeddings (integrated and non integrated), and that other fairly common TFs did the same (**Figure 4d** and **Extended Data Fig. 9a-b**). The observed difference in trajectory lengths means that non-linear alignment is essential, however we would underline that the requirement that the mapping is monotonically increasing (i.e. retains the same temporal ordering) is a very strong constraint on the algorithm.

To address the reviewer's concern that flexible warping functions might impose artificial concordance across trajectories, we tested whether the choice of reference affects the internal organisation of transcriptional dynamics. We reasoned that longer trajectories are more likely to span the full proliferative period of larval neuroblasts and therefore provide more reliable references; accordingly, we additionally used 04B_T2 and 14A_T3, both corresponding to long trajectories, as alternative

references. Qualitatively, the expression patterns of shared transcription factors after warping using different references show no major differences (see **Reviewer Fig. 1** for representative examples).

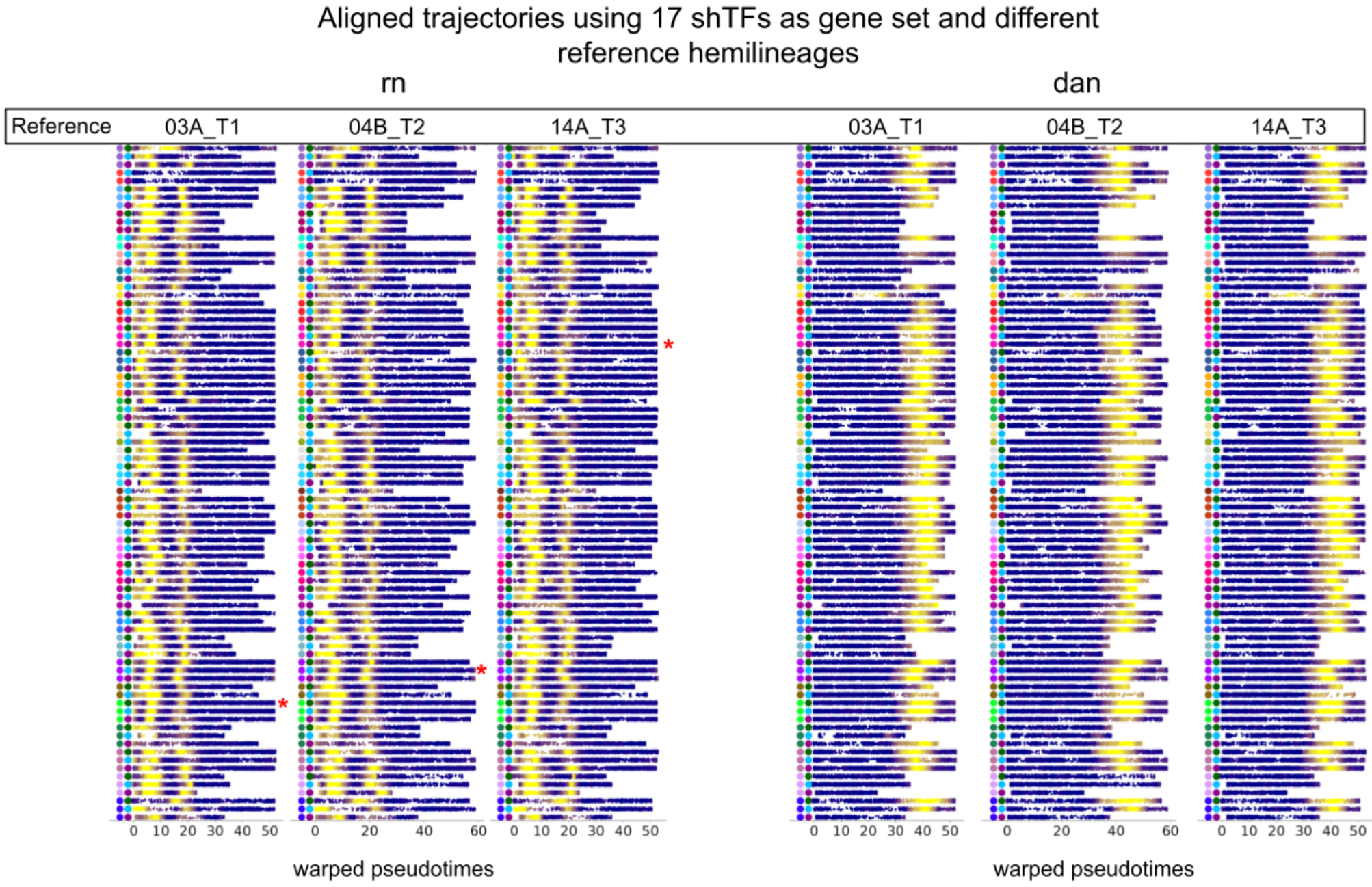

Reviewer Fig. 1: Alignment robustness to reference choice.
Heatmaps showing the expression (normalised between q05 and q95) of two shTFs (rn and dan) after G2G alignment using the 17 shTFs identified in the paper, but 3 different reference trajectories. Red asterisks indicate the specific trajectory serving as reference in each case.

We then performed a quantitative assessment of alignment robustness by measuring, for each gene, the preservation of relative expression relationships along pseudotime across independently aligned trajectories using two approaches: self-similarity structure and rank-based pseudotime invariance.

For the first approach, we computed the self-similarity structure of each gene within a trajectory, capturing the internal pattern of expression relationships along pseudotime. For the second approach, we normalised pseudotime within each aligned trajectory to a common scale (0–1), binned expression profiles into 30 pseudotime bins, and compared the rank order of expression values across bins.

For both metrics, we assessed concordance across reference choices using Spearman's rank correlation, taking 03A_T1 aligned with the 17 shTFs as the primary reference (**Reviewer Fig. 2**). In addition, we applied both metrics to compare alignment results for 03A_T1 obtained using either the 17 shTFs or the larger set of 40 transcription factors shared by ≥50% of hemilineages used in the initial analysis (shown in blue in **Reviewer Fig. 2**). Across the majority of trajectories, both self-similarity and rank-based measures showed high concordance across reference choices and gene-set selection, indicating that the alignment preserves intrinsic temporal organisation of gene expression rather than introducing over-registration artefacts.

The alignment algorithm gene-to-gene (G2G) has limitations, in particular we identified a small number of hemilineages for which alignment is suboptimal (notably 20A and 22A). These cases most likely reflect incomplete temporal coverage, where major temporal domains are absent and too few shared genes are expressed along the trajectory to support robust alignment, rather than a lack of underlying transcriptional structure. Future experiments will confirm/confute this hypothesis. We nevertheless retained these hemilineages in the analysis to avoid selective exclusion and to provide a conservative assessment of alignment robustness.

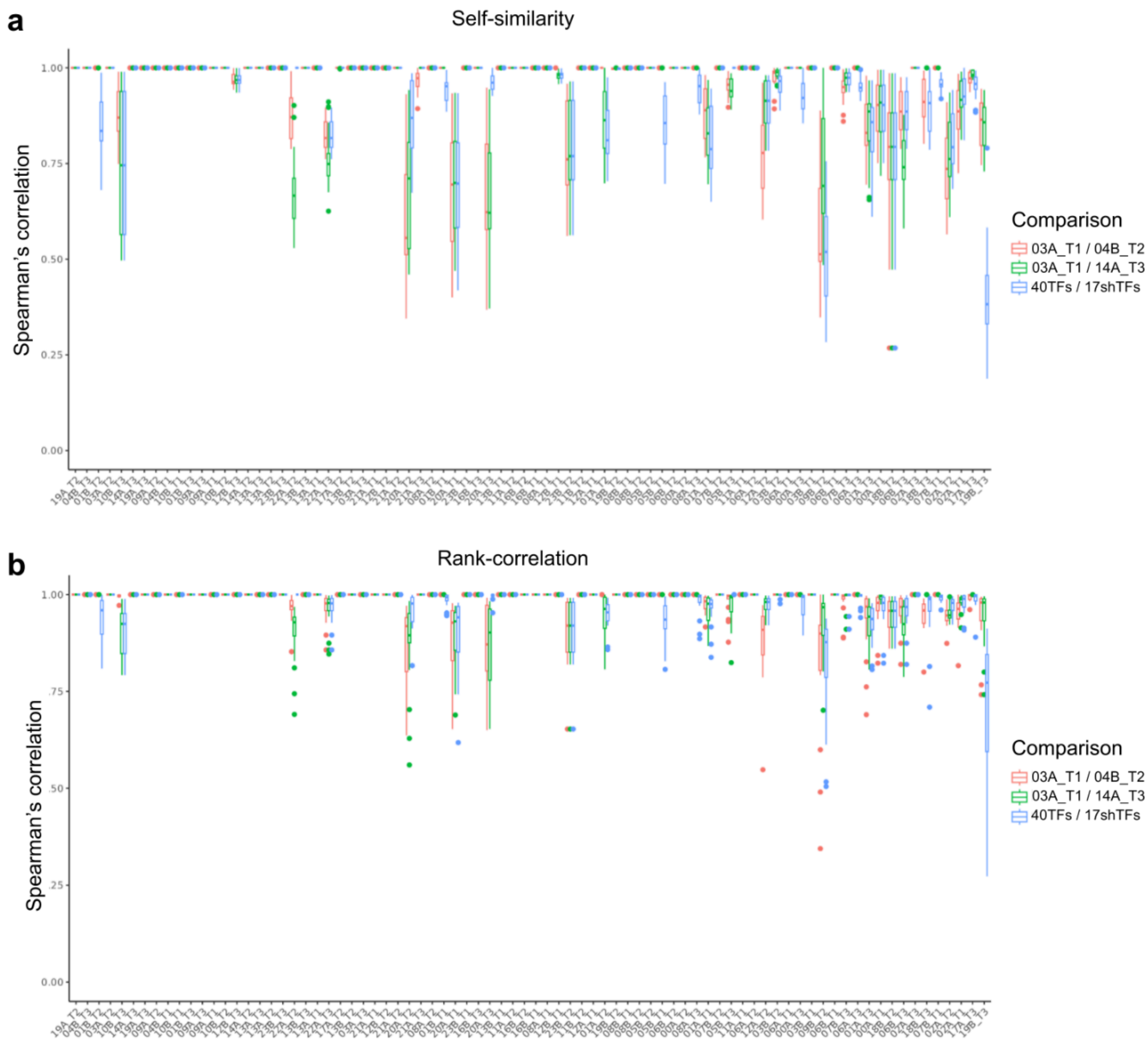

Reviewer Fig. 2: Concordance across reference choices.
Box plots show, for each trajectory, the distribution of self-similarity (a) and rank-based (b) concordance values (Spearman's rank correlation) computed for the 17 shTFs when comparing alignments obtained using different reference trajectories. Median correlation and interquartile ranges across the 17 genes are shown. Reference trajectories themselves are excluded from the analysis. Trajectories are ordered from left to right by decreasing pre-alignment pseudotime span.

1.3. Inconsistency in the expression patterns of the 17 TFs shown in different figures. The expression patterns of shTFs, such as Eip93F, Pdm3, Pros, Mamo, ab, CG3726, br, bab1/2, dan/danr shown in Fig 4g heatmap is strikingly different from those shown in Extended Data Fig. 9. For example, Mamo is shown as expressed around psedotime 40 (late-born neurons) in Fig 4g, but expressed in psedotime 0-10 (early-born neurons) in all hemilineages in Extended Data Fig. 9. In addition, in Fig 4g, the y-axis of the bottom panel appears misaligned; and heatmap on the top appears not consistent with the peak map on the bottom, for example: CG3726 is expressed at a late pesudotime in the 03AT1 heatmap, but is shown as an early "active peak" in the bottom; Dan is expressed at very early pesudotime in the upper heatmap but the active peak is placed at a late pseudotime in the lower panel. These discrepancies undermine confidence in the shared-pseudotime alignment, and raise doubts on the global temporal code hypothesis.

We are –sorry that the reviewer received a version of the manuscript with the wrong figure. To explain the source of the error: v1 of the manuscript deposited on bioRxiv contained the correct version of the

figure, in v2 (the version initially submitted to the journal) the labels, but not the expression, were erroneously shuffled. When we noticed this, about two weeks after the initial submission, we corrected the panel, submitted a v3 version on bioRxiv and asked Nature editorial assistants to notify reviewers about the change. The inconsistencies noted by the reviewer are all explained by the labelling error.

1.4. Verification and functional significance of the "global temporal code." Experimental verification of the protein expression patterns of at least some of these proposed shTFs in neurons born at different times will greatly strengthen the conclusion. Also, there's no direct perturbation showing that specific shTFs instruct fate or maintain identity. To functionally validate the temporal code, perturb at least one early and one late shTF (or a small combo) in a chosen hemilineage; and show predictable shifts in neuronal identity (molecular markers, morphology, and/or EM type match).

We thank the reviewer for the suggestions. We have now done experiments to address the 2 main points raised.

1. Experimental verification of protein expression patterns in adults for neurons born at different times.

We probed the expression of shTFs using co-stainings for 9 out of the 17 shTFs for which we could source antibodies (shown in **Extended data Fig. 11**, **[lines 428-431]**), confirming their expression at the protein level in adults, in both VNC and central brain. We also did a limited number of pupal stainings confirming expression during development, which are not included due to space constraints. In most cases, the result of the co-stainings align with the predictions from the atlas, showing limited colocalisation for shTFs expressed in different, non-overlapping pseudotime windows (e.g. Dan-Eip93F and Mamo-Bab2). Unexpectedly, while in the transcriptional atlas Eip93F and Br display considerable overlap, we could not detect co-expressing cells in our stainings. This result suggests the existence of genetic interactions between shTFs, potentially refining post-transcriptionally the temporal domains.

We tested the link between shTFs expression and birth-time by EdU feed-chase experiments. We focused on Br and Bab1, expressed in non-overlapping pseudotime windows. We tiled the period from 57hALH to 105hALH, where the bulk of larval neurogenesis takes place. Beyond 105 hALH there is still neurogenesis but it cannot be studied by EdU feeding, as larvae stop eating in preparation for pupation (Truman and Bate 1988). We found Br+ neurons colocalising with EdU in animals fed between 69 and 85 hALH time windows, while Bab1+ cells display a complementary pattern, enriched in earlier, 61 hALH, or later timepoints, 104 hALH, in agreement with predictions from the atlas (**Fig. 5a-d, lines 432-436**). Importantly, while not quantified, qualitative assessment of the central brain aligns with the VNC results. These results support the proposed "global temporal code", which is well positioned to specify cell types across the VNC and central brain.

2. Functional validation of the shTFs in neuronal specification.

Several studies have already uncovered cell specification functions for several of the shTFs in specific contexts. Some of them are:

- Ozel et al 2022 showed that pdm3 is necessary and sufficient to convert Tm4 to Tm2.
- Smolin et al 2024 showed that br-z4 isoform is capable of transforming LPC2 into LPC1, two cell types connecting visual information to brain circuits and developmentally originating from CB neuroblasts, thus providing evidence for a functional role for br in the context of the shTFs code in the CB.
- Elkahlah et al 2025 recently showed that pdm3 is able to affect the number of br+ cells within the CREa1B lineage when overexpressed outside its domain. In the v2 of this bioRxiv paper, they have also shown that overexpression of dan can alter morphology of neurons in the CREa1b hemilineage.
- Wani et al 2026 showed that Eip93F is required for the specification and adult maintenance of neuronal properties for dorsal fan shaped body neurons which derive from type II central brain neuroblasts.

We have added a paragraph in the discussion summarising these results and pointing to the need for further investigation of the functional role of the other shTFs in specifying cell type identity **[lines 658-662]**.

We performed additional experiments to test the role of *br* in cell type specification in the VNC **Fig. 5e-f, [lines 441-453]**. We ectopically expressed br-z4 across all neurons of hemilineage 14A, identifying a change in the morphology but not in the number of neurons, compatible with cell type conversion. We also revealed the loss of expression of Bab1, which is normally non-overlapping with Br. These results support a role for br in specifying neuronal identity in the VNC.

Minor Comments

1.5 Figure 1C color palette.

The current palette uses similar hues across stages, especially after regression, making it hard to distinguish timepoints on the UMAP. Use a color-blind-safe palette with maximally separated hues and consistent legends across panels. This will help readers to visualize where the cells of different pupal stages are located in the trajectory, and accurately assess if there is any contribution of maturation to the expression patterns of shTFs.

We agree with the reviewer that the use of similar hues is not ideal to distinguish timepoints in the UMAP. However, we thought it could have been a good way to convey the concept of a continuously varying variable in the context of an already very colorful figure. We have now changed the stage colour palette consistently across the manuscript to a color-blind-safe one, with maximally separated hues.

While we hope that the new colouring scheme aids assessment of the integration quality, for a more accurate evaluation of the contribution of development to the expression pattern of shTFs we invite the reviewer to refer to **Extended Data Fig 9a-c**, where expression levels of a subset of shTFs can be observed in non stage integrated UMAPs (i.e. in a context where stage variability contributes to transcriptional heterogeneity of the dataset). If the expression of shTFs was affected by the maturation stage of the cells, one would expect it to slide along concentrically arranged, stage-specific trajectories and to occupy different locations in relation to bona fide cell-type-specific markers such as neuropeptides. However this is not the case. In the newly added **Extended Data Fig. 9c** we provide an example of this for hemilineage 04B, which is characterised by the presence of several neuropeptide-expressing cells. Here, AstA is expressed before the shTF bab2 (partially overlapping with it) across stages, although at 6h AstA expression is really low (upper panel). Similarly, Dh31 is expressed after Eip93F (and consistently before AstA, bottom panel). We are adding here, for reviewers only, an example of how the expression patterns, and relative positions, of three neuropeptides (AstA, Dh31 and sNPF) is invariant before and after stage integration of 04B secondary neurons (**Reviewer Fig. 3**). For additional examples we invite the reviewer to browse the neurons dataset in SCope (https://scope.aertslab.org/#/HundredDrills/*/welcome), where the expression of different gene combinations can be easily inspected in the integrated (umap) and non-integrated (UMAPallages) embeddings.

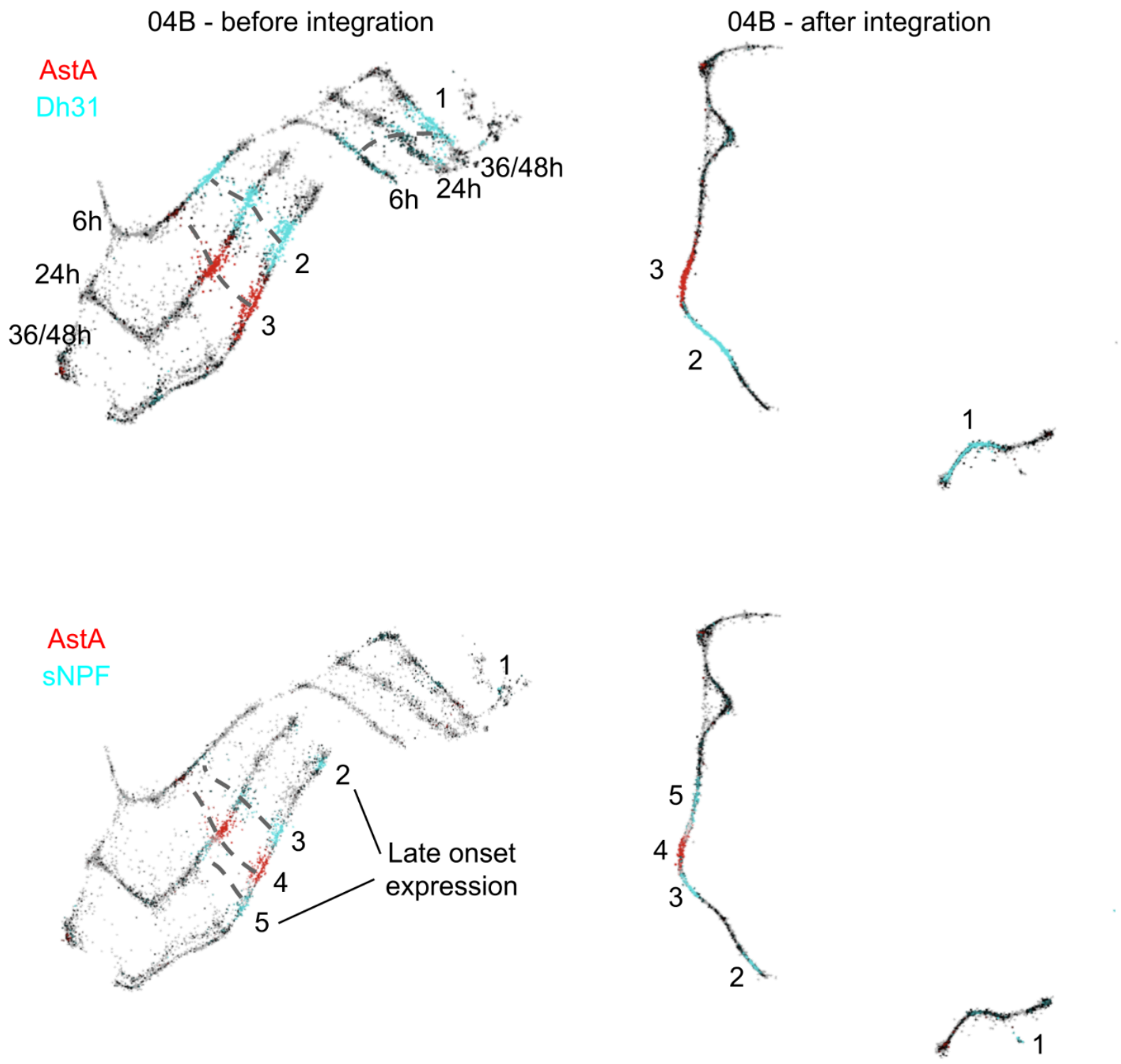

Reviewer Fig. 3: Relative positioning of cell types across developmental stages.
UMAP plots of 04B hemilineage showing expression of 3 neuropeptides (Dh31 and sNPF in cyan and AstA in red) before (left) and after (right) stage integration, demonstrating the conserved relative positioning of the shared TFs to neuropeptide-specific cells across stages and after integration.

1.6 Extended Data Fig. 6b: stage clarity. Explicitly label the developmental stage(s).

We have added the label L3 in the panels titles and to the legend. We have also changed the colour of the expression levels summary to better match what reported in **Supplemental File 4**.

1.7 Provide a Data & Code table (accessions, commit hashes, environment) to maximize reuse of the resource.

We have added a Data Availability section [**lines 1795-1804**]. Raw sequencing data, CellRanger outputs as well as processed Seurat objects, are available on the GEO repository (GSE304221) and will be made public upon publication. The GEO repository is currently accessible at https://www.ncbi.nlm.nih.gov/geo/query/acc.cgi?acc=GSE304221 using the private token gloxmmiyttgxrcj.

We also provide a link to the dimensionality reduction viewer SCope (https://scope.aertslab.org/#/HundredDrills/*/welcome), where users can interactively explore different subsets of the data within the embeddings presented in the manuscript. Loom files have also been uploaded on Zenodo. We expanded the list of packages used to analyse the data (**Supplemental File 7**), including versions. Additionally, we created a GitHub landing page for the paper with instructions on how to download and inspect the data.

1.8 Line 986, Method part, "Hemilineage identity was then further confirmed by published split lines". Please include a detailed description for which hemilineages, identities have been confirmed using this method and the split lines used.

With the exception of the newly added fer3-tey splitGal4 combination presented in **Extended Fig. 8**, which confirms expression in the predicted hemilineages, we did not perform new validation stainings. Instead we compiled and referenced previously published split-GAL4 lines whose expression patterns and hemilineage identities were experimentally validated in earlier work (Soffers et al. 2025). To make this explicit, we have revised the text [**lines 1385-1387**] and added a dedicated column in

Supplemental File 1 reporting the corresponding published split-GAL4 combination [Drivers combinations in Soffers et al. 2025]. We hope this clarification resolves the misunderstanding.
1.9 Line 1020- 1024, "In cases where immunostaining data from L3 larvae indicated that expression of Antp and Ubx genes deviated from the canonical model, we used these data as guidance". Please list all hemilineages for which these data are available, in addition to those shown in Extended Data Fig. 6.
We now point the reader to **Supplemental File 4 [line 284]**, to which we added a column with the observed expression data across many stacks.

Referee #3:

How neuronal diversity is generated in the cell lineages of neural stem cells is an essential question in neural development. This paper addresses this question using the ventral nerve cord of Drosophila, generating a large single-cell atlas of neurons at several stages of development. There are several conclusions:

3.1- Neurons that are generated sequentially express different transcription factors almost independently from where the stem cell originated within a segment or which segment they come from.
This argument of time-dependent TFs has been made several times in other papers as early as 2011 (e.g. 'concentric genes' in DOI: 10.1242/dev.058370) and in more recent publications on the visual system and in the central brain (a very similar paper posted for months in bioRxiv on central brain lineages, doi: 10.1101/2025.06.03.657692).

There are major differences in organisation of optic lobes versus central brain/nerve cord neurogenesis. This is likely driven by the fact that the optic lobes are dominated by a relatively small number of cell types repeated many times across the visual field (~440 intrinsic cell types, 47,000 cells per hemisphere for the male CNS) while the central brain and VNC contain 10,910 cell types for 26,000 cells per hemisphere.

For these reasons, our coverage of the past literature focussed strongly (we agree perhaps too strongly) on the central brain and VNC, since these have the same mode of neurogenesis, which is markedly different from the optic lobe. Prior work for the CB/VNC had not identified a post-mitotic temporal transcriptional code shared broadly across lineages. It is correct that this is a claim of the Clowney preprint which appeared 6 weeks before our own. We became aware of this preprint shortly before submission and were faced with a situation with which the referee may perhaps sympathise – how to cite a brand new but relevant manuscript in the final weeks of a 6 year project. While our standard approach would have been to highlight these new results in the Discussion, we were faced with something of a dilemma by severe limitations in the Clowney study. The Clowney preprint claims to define a sequentially expressed series of transcription factors, but the evidence for this sequential expression is extremely weak. Indeed the relevant panel (Fig 4F) from v1 of their preprint has now been removed from their v2 preprint (posted 7th January 2026, presumably after peer review). Furthermore, the preprint did not claim evidence of a *global* transcriptional code. These limitations arose from lower coverage, limited developmental stages, and the inability to identify the vast majority of brain stem cell lineages (they only collected data for 13 reproducibly labelled + 13 less frequently labelled of the ~100 lineages generating the CB). Having had the time to evaluate the Clowney preprint as well as a related study from the Goodwin lab, we now cite both in the discussion **[lines 680-683]**. However, while these studies are consistent with our own findings, we must underline here that the evidence that they provide for a global code of sequentially expressed transcription factors within the brain remains very weak compared with our own study in the VNC, further strengthened by additional validation of their time-resolved protein-level expression (see response to point 1.4).

After early discussions with a collaborator working on optic lobe, we had hesitated to draw an analogy with the “concentric genes” because these genes are largely restricted to the main outer proliferation centre (OPC), but apparently not shared by tOPC or IPC domains. Furthermore at least some of these genes (e.g. Lim3 and tj) appear to be specific for Notch-OFF neurons born in their temporal domain. In contrast, shTFs are expressed across all neuroblast lineages in VNC, and likely also CB, and do not depend on Notch status i.e. they have the characteristics of a global code for thousands of cell types rather than perhaps 50 cell types each for the concentric genes. However, in light of the reviewer’s comments, we have further discussed this point with optic lobe experts and agree that the analogy between shTFs and “concentric genes” remains conceptually useful, as both sets of genes are expressed by neurons born in specific time windows, although important differences exist. We thank the referee for this recommendation and have added a reference in the Discussion **[lines 671-674]**.

3.2- A major point made in the abstract is that the number of neurons identified by the connectome grossly outnumbers the number of cell types defined by transcriptomics. However, this argument is based on totally outdated papers (2018! ref 24-26) and the argument is not valid as in-depth developmental atlases show close (but not perfect) matches to the connectome. This is discussed below (and should be discussed in the paper!).

There are no references to connectomics outnumbering transcriptomic cell types in the abstract. But the point does feature in the introduction to highlight the limitations of existing large scale central brain and VNC datasets for which the 2018 papers remain the largest publicly available datasets. Our point that these datasets remain underpowered compared with the huge complexity of cell types in the CNS remains valid.

We excluded discussion of the coverage of optic lobe data when we finalised in the introduction because we do not believe these data speak strongly to the ability of transcriptomics to recover the >10,000 CNS cell types identified in connectome data. The very latest data from the Ozel/Desplan groups preprinted in Sep 2025 (Coyne et al) identify 113 connectomic cell types out of 233 clusters that can be resolved in OL scRNAseq data. In the male CNS connectome, the mean number of cells for the 440 cell types in each optic lobe is >100. After inspection of the matching table from Coyne et al we identified Tm23 as the smallest type cross-matched (16/17 neurons per hemisphere in maleCNS). Just 29 (0.27%) of the 10,910 cell types present in the rest of the male central nervous system connectome are this numerous. Therefore, despite the notable success of the optic lobe field in molecularly defining almost 25% of the OL cell types, the challenge in the rest of the CNS remains huge.

Of note, we do cite both visual and olfactory system studies at other points in the manuscript, including for their demonstration that developmental atlases can better capture enough diversity for complete matching of cell types between connectome and transcriptome. We have revised the Introduction by adding clear references to extensive atlasing efforts in the OL, where numerous and well characterised cell types are present in repeated copies, while highlighting the challenges emerging when tackling the rest of the nervous system, where most types are present only in low copy number **[lines 70-88]**.

3.3- Another important argument is that larval born neurons show a continuum of cell fates while embryonic born neuron types are more discreet. This argument has been made in mammals and is most likely due to insufficient depth of sequencing (in spite of the 38X coverage, see below).

This was primarily a language issue and in particular we apologise for confusion arising from our use of the word “continuous” (which has some history) was unfortunate. We have now changed ‘continuous’ for ‘gradual’ throughout the manuscript. Crucially, what we are proposing is that there is a

quantitative difference in the degree of separation and molecular organisation between primary (embryonic born) and secondary (larval born) cell types. This variation is more gradual and organised along an obvious low-dimension manifold for secondary neurons. This observation holds regardless of coverage, i.e. secondary neurons are next to each other while primary neurons are further away, increased coverage might show that secondary cell types are actually very close to each other without being continuous, but they will still be more similar than primary neurons. Crucially, we see the same in the connectome, secondaries are more similar than primaries, very strongly supporting the transcriptomic results. See response to point 3.7 for more details.

3.4- The paper also explores sex-dimorphic neurons and shows that male and female neurons can have different fates, including death. This has been documented in the past and in the recent bioRxiv paper above. This is an add-on to the paper that is not necessary.

We do not think that the section on sexual dimorphism is an unnecessary add-on to the paper, rather it provides a strategy to refine the match between transcriptomics and connectomics (e.g. it allowed us to cross-match fru+ neurons at nearly cell-type resolution as shown in Fig. 6g). Thanks to the comparative connectomics approach, it provides a valuable resource where connectomic and transcriptomic dimorphisms can be interrogated at scale. It also confirms a number of predictions that can be made starting from the analysis of the developmental transcriptional atlas, further supporting the quality of the dataset and its annotation.
As the reviewer points out, the cell death observation is not new per se, but our analysis shows for the first time its widespread use in the VNC. We have added references to recent studies reaching similar conclusions on the developmental mechanisms driving sexual dimorphism in the central brain [**lines 595-597**]

3.5- In general, one major issue with the paper is that the authors appear to not be aware of, or to ignore results from several groups that have done similar developmental transcriptomic analyses on the visual or olfactory systems (papers cited but not discussed appropriately) that present a much more favorable situation because neurons of each type are present multiple times.

As the reviewer rightly points out we cited those papers and we would argue their important conclusions for framing our work were discussed. Due to space constraints and because of the type of discussion we wanted to give the manuscript, we chose to not comprehensively list similarities and differences in terms of genetic strategies and players between cell types specification in the OL and VNC.
Furthermore, as mentioned before, what the reviewer considers an experimental advantage of the visual system, the high degree of cellular repetition, looks to us as a fundamental difference between the OLs and the rest of the CNS, where cell types are present in 2-3 cells per side (see 3.2). We then asked, can we extrapolate what we learnt in the OL to the entire CNS? We think different parts of the nervous system need to be examined on their own merits; for example the genes we identified as operating across the central brain and VNC (shTFs) are either not deployed or, given their expression patterns, are likely to fulfill different roles in the OL. Conversely concentric genes or other important OL genes are likely, given their expression patterns, to fulfill different roles in the CB+VNC.

The bioRxiv paper above makes very similar points and should also be referenced.

The papers have now been cited. Please refer to 3.1 for further assessment of the evidence supporting this point in Clowney's paper.

These papers made similar points, or contradict some of the most important (related) statements made here, e.g. continuum of cell types, and discrepancy between transcriptome and connectome.

We no longer refer to continuum and instead talk about “gradual” to better reflect the changes between types of secondary neurons observed in the connectome (see also 3.3 and 3.7). We don’t think our conclusions framed this way contradict previously reported conclusions in the OL, but rather reflect the different cellular organisation of these regions of the nervous system as also observed in the connectome.

Evaluation:

3.6- One strength of the paper is the 38x depth of scRNAseq, with several hundred thousand neurons. This is necessary since each neuron is represented only once or a few times bilaterally in each organism, which makes it difficult to cluster efficiently. The data are of quality. However, the 38x coverage includes all stages merged together. This is problematic as the same resolution cannot be attained early on (P6), no matter the number of cells because newborn neurons are much harder to tell apart from each other, especially if they are developmentally related (diversity will increase at the time of synaptogenesis). This is supported by the fact that the authors do not observe the continuum for mature primary neurons.

We agree that not all different developmental stages present in the dataset have the same power to resolve cell types. Nevertheless, while a minority of neurons at 6 and 24h remain undifferentiated, the majority (> 60% at 6h and ~80% at 24h) are located in trajectories rather than in the “precursor” region. Interestingly, trajectories become longer as development progresses, likely due to further increases in transcriptional diversity. However, key cell identity markers such as neuropeptides, maintain invariant positions in relation to one another, indicating that cell types don’t “slide” along trajectories as maturation progresses (see also response 1.5). Previous work has shown that cell types can be identified in poorly differentiated stages (0h) or adults, when using markers from the most discriminative developmental period (i.e. Xie et al in 2021 for PNs, and Ozel et al. 2021 and Kurmangaliyev et al. 2020 for OL), which served as rationale for our integration approach. Thus, while most cells in trajectories from 6 and 24h have already reached a maturation stage suitable for identification of their end-point type identity, especially if “guided” by more mature instances of themselves, existing at later developmental stages. We have now changed the way we refer to 38x coverage: while we had previously used “global” coverage we now talk of “aggregate” coverage and refer to **Extended Data Fig.1f** for per-stage coverage [**line 175**].

The reviewer also suggests that primary neurons fall into discrete clusters rather than extended trajectories due to the fact that they have had more time to mature. While we certainly considered this explanation, multiple lines of evidence argue strongly that this is not the case.

1. The birthtime difference between late born primary neurons and early born secondary neurons is less than the difference between early born secondary and late born secondary and still all secondaries populate trajectories, in integrated as well as non-integrated UMAPs (and upon integration of only late developmental stages, as shown in **Reviewer Fig. 5**).
2. While constituting the larval nervous system, primary neurons undergo substantial reorganisation (pruning and regrowth) before being integrated in the adult one. This is reflected by the strong expression of the regulator of pruning Sox14, which is strongest at 6h (**Reviewer Fig. 4**). These cells therefore undergo a new cycle of synaptogenesis, which could re-set their identity. We have added in Fig. 1c an example of nSyb expression in non-integrated space, showing that nSyb levels increase with developmental stages in both primary and secondary neurons.
3. Similar differences between Imp+ (primary) and Imp- (secondary) cells are also present in a recently published atlas of the adult CB (ref. to Fig. 2d and Fig. 2k in Allen et al. 2025).
4. Crucially, the same differences observed in the transcriptome exist in the connectome, a fact that should not be brushed away (see also response to 3.7).

5. The genetic mechanisms for specifying primary and secondary neuron identity (Imp/Syp vs transcriptional cascades, see Discussion of the current manuscript) are different, opening the door to other differences down the road such as the ones we observe.
6. We note that the larval nervous system is numerically much smaller, with many cell types present in one copy. This provides a potential functional justification for the discrete nature of its cell types.

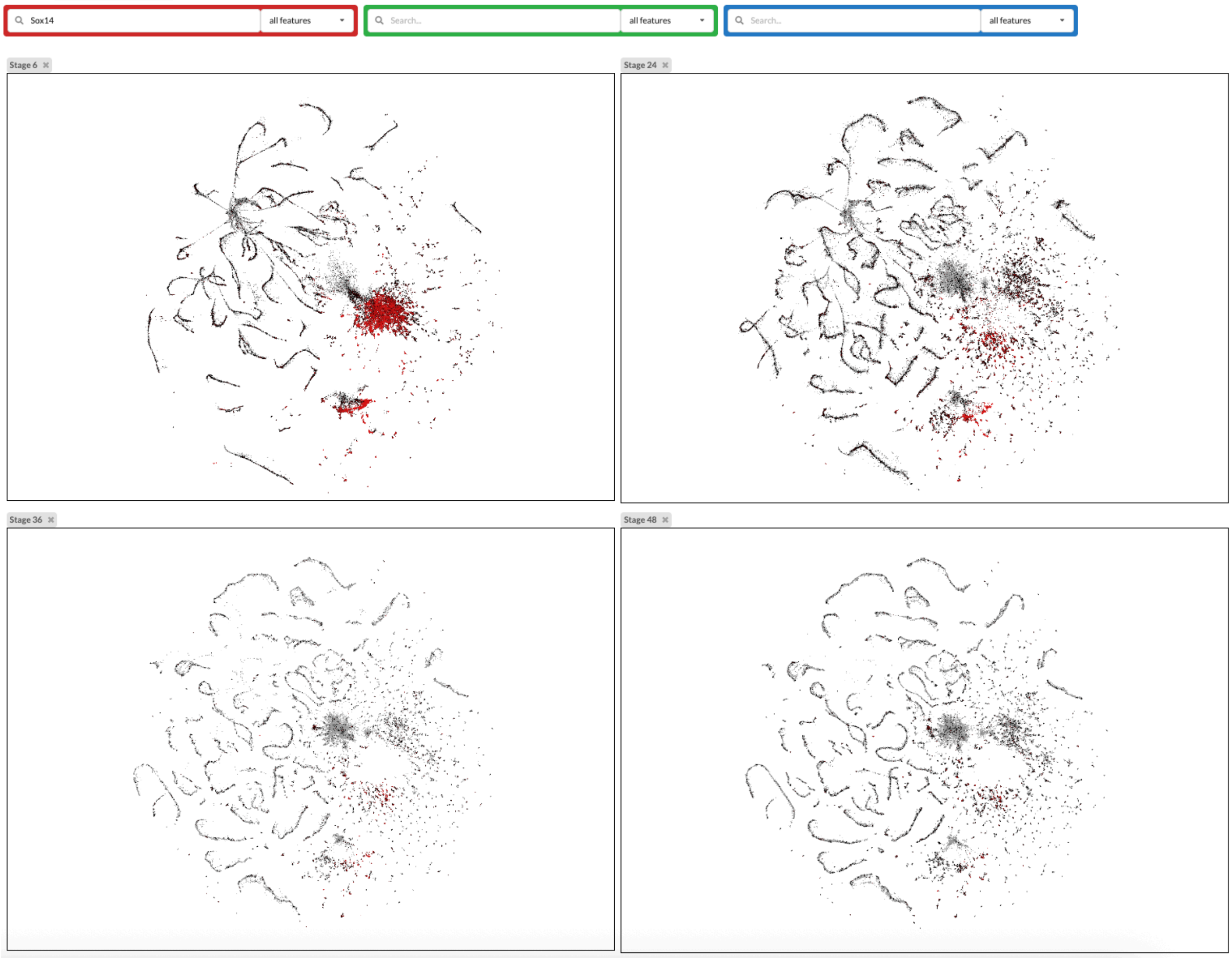

Reviewer Fig.4: Sox14 expression across development.
Sox14 expression is restricted to primary neurons and strongest at 6h APF. Plots show non-integrated UMAP, with cells from each stage are plotted independently.

It is suggested to re-do the clustering analysis using only the late, or P36+48 datasets (when all neurons had enough time to differentiate from their terminal division) and the continuum might disappear, although now the coverage at these stages might not be sufficient to resolve individual cell types. In general, one should be much more careful about overinterpreting UMAPs and their depth, which is commonly done in mammalian data that are almost always underpowered.

We have re-done the analysis as suggested by the reviewer. The re-analysis supports our initial conclusions. **Reviewer Fig. 5** shows that there is no qualitative difference in the organisation in the UMAP space of primary and secondary groups, or of different hemilineages, when compared to our original figure. We would also like to highlight that in the manuscript the data are integrated using 24h as a reference set, which should minimise the effect of poorly differentiated 6h neurons in the integration process. While this information was present in Methods, we have now added it to Fig. 1c legend. We have also added the following points to discussion:
1- secondary hemilineages are not always represented by a single trajectory, but can have discontinuities **[lines 636-638]**;

2- point the reader to the analysis of individual hemilineages at 48h only (Extended Data Fig. 7) where local thickenings can be observed, which might correspond to more abundant cell types **[lines 638-639]**.
3- acknowledge the potential limit of our atlas coverage that, while remaining exceptional, might not be sufficient to fully separate connectomic types of secondary neurons **[lines 639-641]**.

We agree with the reviewer that existing mammalian datasets are mostly underpowered, we have now shortened the parallel to mammalian studies and moved it from Results to Discussion. Nevertheless, the gradual, lineage-related changes observed in the Drosophila secondary neurons along transcriptional and anatomical axes may be conceptually related to reports of dorsoventral transcriptional "gradients" in mammals (https://doi.org/10.1016/j.neuron.2019.11.004). Discretisation of these gradual changes into stereotypical types, will probably only be clearly evident when we can link anatomical (connectomic) and molecular cell types at scale.

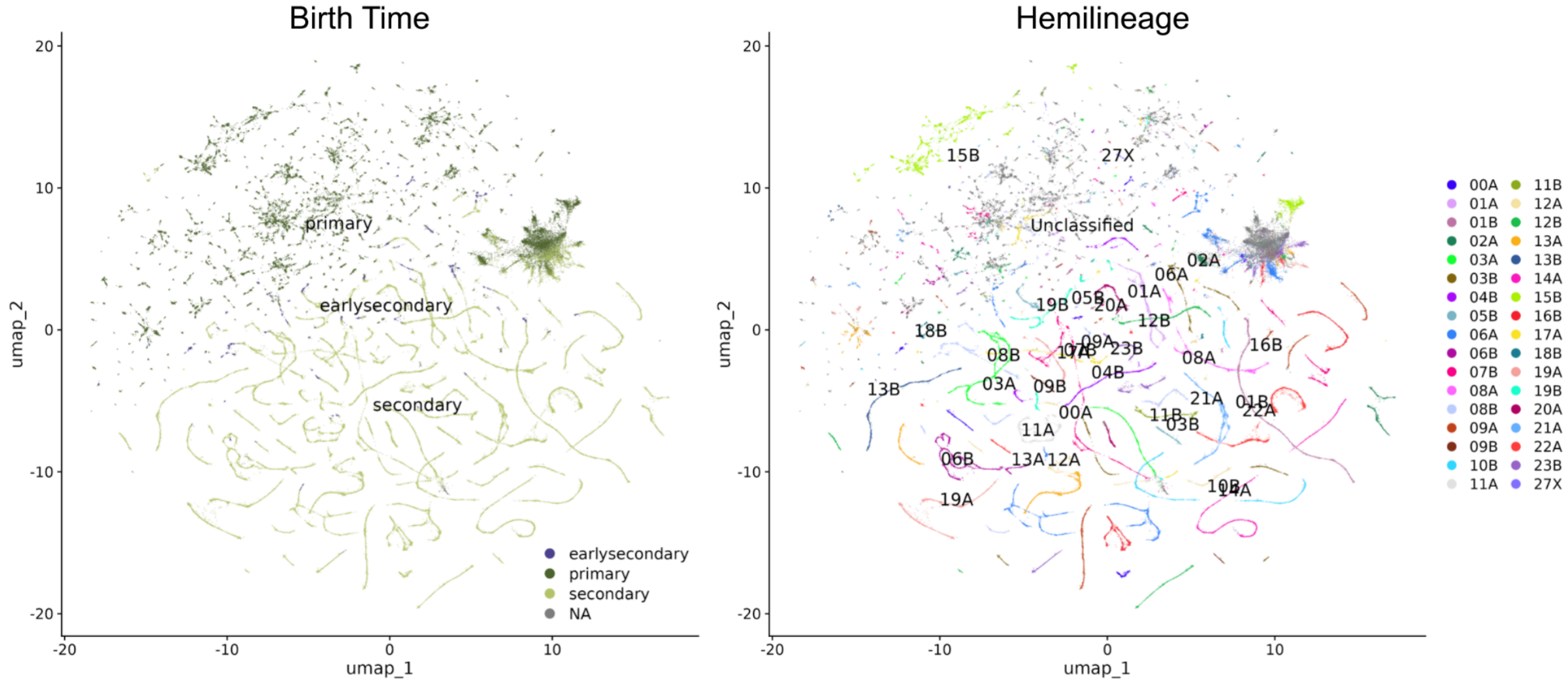

Reviewer Fig. 5: Integration of 36h and 48h datasets using 48h as reference.
The displayed metadata correspond to the annotation described in the manuscript.

3.7- A significant result is the distinction between early embryonic mature neurons and the late larval neurons born immature neurons formed after the neural stem cells (neuroblasts) have been reactivated.
The authors then conclude from this separation that embryonic born neurons are discreetly specified while larval born neurons form continuous trajectories. However, the demonstration of this continuum, which would reflect the fact that there is no transcriptional distinction among neurons, is not clear.
The same argument of a continuum has been made for scRNAseq of human brain neurons where clusters are most often poorly separated due to insufficient depth (e.g. 3,000 neuron types in the mouse brain, which is almost as many neurons as have been defined in current transcriptomics in flies!). The fact that there are more cell types in mice than in humans illustrates this as well. Furthermore, neuronal specification in Drosophila is fairly rigid, with little reported examples of plasticity. Thus, the continuum likely reflects the fact that even 38X (with the caveat mentioned above about focusing only on late time points) is not enough to separate closely related neurons and the authors should be very careful making the argument of a continuum as this will likely be dismissed when deeper sequencing is possible.

We would like to reiterate that while the reviewer ascribes the difference we highlight between embryonic-born neurons and larval-born neurons to their difference in maturation stage, we strongly believe there are persistent genetic, anatomical and functional differences identifiable also in adults between these two subpopulations.

As mentioned in point 3.3 the use of the term "continuous" was driven by the fact that gene expression variation within hemilineages follows a very obvious low dimensional manifold. We have carefully reviewed the language that we used, substituting the word "continuous" with "gradual" (i.e. we don't mean that secondary cell types are not going to be separable based on gene expression). We have now added a sentence clarifying that further increase in coverage might better separate secondary types **[lines 639-641]**. What we do conclude (and we think evidence is very strong for this) is that there is a clear qualitative and quantitative difference in the degree of separation of primary vs secondary cell types by gene expression, i.e. the distance between one neuronal type and the next one born in the sequence is significantly less pronounced in secondary neurons than in primaries. Increasing coverage would not change this point.

As suggested, we have repeated the analysis shown in Fig. 1h using only 36h+48h data to exclude the contribution of less differentiated neurons, and recapitulating the same results (**Reviewer Fig. 6b**). We also quantified the median distance in PCA space for primary and secondary neurons to their k^{th} nearest neighbour, which increases faster for primary than for secondary neurons (**Reviewer Fig. 6c**), further supporting the existence of transcriptional heterogeneity differences between primaries and secondaries.

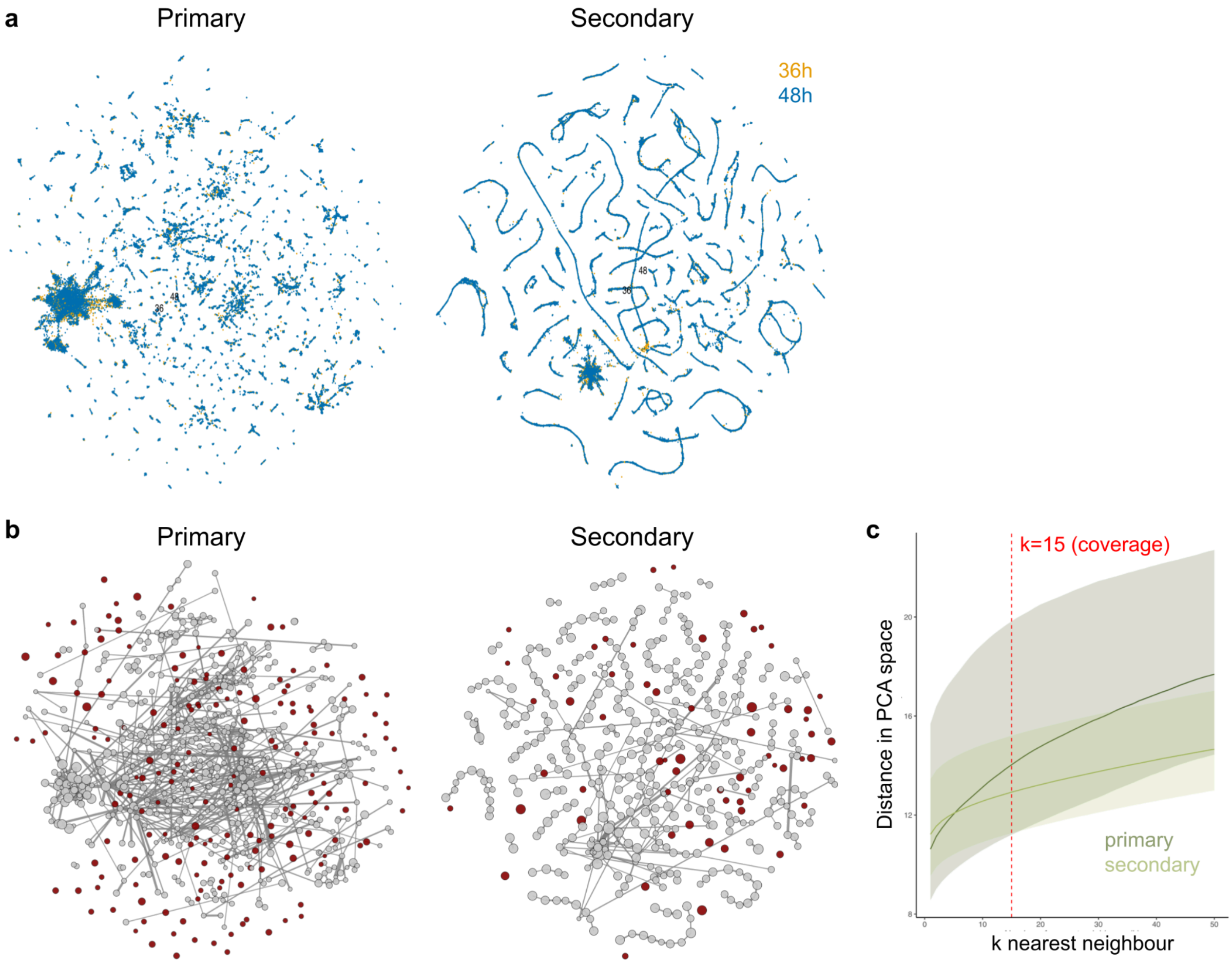

Reviewer Fig. 6: Differences between primary and secondary neurons in transcriptomics space limited to 36h+48h

a, UMAP embedding of primary and secondary neurons from 36h and 48h only after stage variability regression (using 48h as reference). **b,** Constellation plots showing inter-cluster overlaps in primary and secondary neurons, equivalent to what shown in Fig.1h, but using kNN=10 due to the reduced coverage of the dataset after removal of 6h and 24h. Identical parameters have been used for both subsets. Brown clusters don't have any shared neighbours to other clusters. **c,** Distance to nearest neighbours in PCA space for primary and

secondary neurons. Median and interquartile ranges are shown. For each cell, transcriptional distance diverges faster in primary than in secondary neurons.

Crucially, the molecular findings are also reflected in the connectome, as quantified in **Fig. 1i**. We have now added **Fig. 1j**, illustrating the point that the nearest neighbors of secondary neurons are more likely to share the same developmental origin (hemilineage identity) than those of primary neurons. This is further illustrated in **Reviewer Fig. 7**, where we provide (a) some examples of neighbouring neurons in the connectome, both at anatomical and connectivity levels, to help communicate this point and (b) a quantification of the connectivity similarity to the kth nearest neighbour across primary and secondary neurons, showing that secondaries are persistently more similar at increasing values of k.

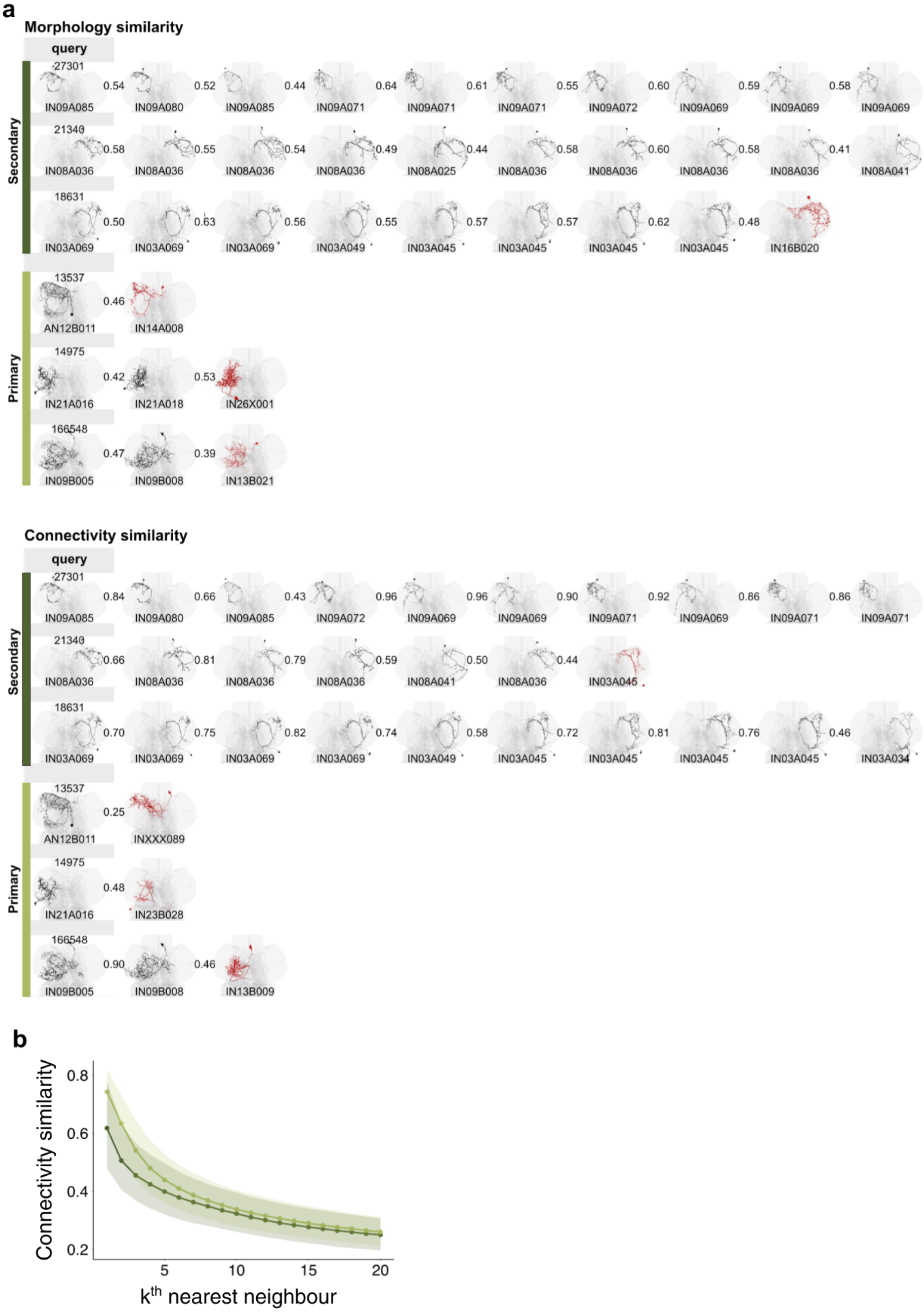

Reviewer Fig.7: Similarity across nearest neighbours in primary and secondary neurons in the connectome

a. Examples of similarity chains for primary and secondary neurons. Starting from a query neuron, we identify its nearest neighbour in either morphological (NBLAST similarity; top) or connectivity space (cosine similarity;

bottom). The chain is then extended iteratively by selecting the nearest neighbour of the most recently added neuron, choosing the second-nearest candidate if the first has already been included in the chain. Chain construction terminates when a neuron belonging to a different hemilineage is encountered. **b.** Connectivity similarity to the k-th nearest neighbour. The graph shows the median and interquantile range for primary and secondary neurons.

3.8- The authors argue that adult scRNAseq grossly underestimates cell types because genes encoding connections between neurons are no longer represented in the adult. This is correct and this argument has been made in several developmental atlases on other regions of the brain, olfactory system, visual system and central brain. If these papers show that there is indeed more diversity early, this cannot account for the huge discrepancy between connectome and transcriptome observed in the current paper. The discrepancy in sensory systems is minimal because each neuron type is present multiple times in the brain and can be clustered and subclustered much more precisely to match neurons from the connectome. Therefore, if a few identical neurons can acquire different morphologies either through stochastic processes (e.g. DCN neurons) or by connecting to different neurons, this is not enough to explain the difference with the connectome observed here. A recent paper on the fish retina also made the same argument that functional cell types cannot be distinguished by their transcriptomes (doi: 10.1038/s41586-024-08518-2) but it is likely due to insufficient coverage and the late time when scRNAseq was performed. Thus, as argued above, even the many neurons in the current paper are likely not be enough to cluster related neurons.

One of the important points we wanted to convey was the idea that the transcriptional diversity observed in our atlas has the potential to explain the number of different types observed in the connectome. However it seems from this comment that we weren't clear enough on this point. For example, unlike the zebrafish paper mentioned by the reviewer, we are not saying that connectomic types don't have corresponding transcriptomic types, quite the opposite. At the current resolution, discrete clusters of secondary neurons are not overtly evident in our data, but our working hypothesis is that enough cell type diversity lies along the trajectories to account for the observed neuronal diversity. For example, in Fig7d, we clustered neurons aiming to obtain half the number of cell types described in the connectome as target number of clusters - we did not use the full number of types because 48h data alone only has 10x coverage and clusters can become unstable. Even using a small number of genes (the 17 shTFs) we obtained well-defined clusters along trajectories and we propose in the discussion that shTFs provide a way to further improve the resolution of the match between transcriptome and connectome. However, we were careful to not claim that we identified a specific number of clusters and these matched (or not) the number of types in the connectome as it is beyond the scope of this paper to develop the genetic toolkit to validate those results; without this validation, reporting >3000 clusters seemed to have little value. Instead we annotated the atlas hierarchically, reaching the highest matching resolution for fru+ neurons (Fig. 6f-i, Extended Data Fig. 12).
We have added a sentence to the introduction discussing strengths and limitations of cell-type matching obtained in the optic lobe **[lines 70-84]** and stated in the discussion that a further increase in coverage might unequivocally separate secondary clusters corresponding to connectomics types [**lines 639-641**].

3.9- The manuscript also confirms that the last NotchON/NotchOFF division of GMCs greatly affects the outcome of the lineages and that one should only consider hemilineages. The notion of hemilineages is old, going back to earlier MARCM studies and it has been made in earlier studies that showed that Notch can distinguish between excitatory (on) and inhibitory (off) neurotransmitters (see also the bioRxiv paper referred to above).

We agree with the reviewer that the notion of hemilineage has been known for a long time. We are not claiming merit for their discovery. In addition to the previously cited review articles, we have added the

reference to the original publication describing the role of Notch signalling in determining neuronal phenotypes in secondary lineages (https://doi.org/10.1242/dev.041749).
What we are claiming is that we did not find a universal signature of Notch identity across lineages. In comparison, previous studies in the optic lobe have suggested common features across NotchON neurons, where Apterous is considered a marker for NotchON neurons (https://doi.org/10.1101/2024.02.05.578975, 10.1038/nature12319, 10.1038/s41586-022-04564-w), although there seem to be exceptions there too (https://doi.org/10.1101/2025.09.01.673531). Interestingly in the VNC *ap* is a marker for a NotchOFF secondary hemilineage (04B).
We would like to highlight that in the VNC neurotransmitter identity is not globally bound to Notch status, as it can be appreciated in **Extended Data Fig. 3b**. This is also true in the CB. The example of CREa1 lineage reported in the mentioned bioRxiv, where the NotchON neurons (CREa1A) are dopaminergic and -OFF neurons are gabaergic, is just one example, but -ON neurons can also be inhibitory, as well as -OFF neurons can be excitatory in the CNS. It is completely lineage-dependent. In the VNC there is also an example of both hemilineages being gabaergic (lineage 06).
The main Notch-related take home for this paper is that post-mitotic neurons don't have a pan-Notch signature across lineages.

3.11- The paper defines a series of transcription factors that reflect birth order. The concept of terminal selectors that correlate with birth order regardless of lineage is not a novel concept. (see concentric TFs in the visual system). As mentioned above, the authors avoid any discussion of optic lobe findings that would be important for their arguments. There are always exceptions, because the expression of the same TF can easily be expressed independently in different lineages (see Fig 4g). However, if one should be careful suggesting that a given TF is a perfect marker of a temporal window across an entire brain region, the argument is of interest. The authors should compare their temporal terminal selector TFs with those in the bioRxiv paper mentioned above and with neurons in sensory systems.

As detailed in 3.1 we have now included a reference to concentric genes in the Discussion [**lines 671-674**], highlighting the conceptually analogous role, yet distinct identity and more confined expression. We have also incorporated (1) a reference to an older PNs paper (Xie et al 2021) that had identified some of the shTFs as markers for specific PNs, whose birth order is well known and supports the birth-order to specific shTF expression window relation [**lines 677-679**]; and (2) the reference to the recent CB studies (Elkahlah et al. 2025; Allen et al. 2025), see 3.1. Unlike our work, these two studies analysed only a subset of hemilineages in the brain and therefore they cannot address the generality of the results across lineages. As a consequence, they identified TFs which are not part of the global code identified by us because they are temporally expressed only in a subset of hemilineages in the VNC (e.g. Zfh1 and Zfh2)[**lines 680-683**]. We have added **Table 3** summarising the identification of our shTFs across studies. While not trying to undermine these papers, we would like to highlight a specific strength of our manuscript, namely its global scope.
If the reviewer is concerned about the fact that shTFs are ordered differently in different brain areas, we carefully inspected the order of expression of shTFs across studies (especially those displaying a single peak of expression: br, Eip93F, dan/danr) and found their order of expression is maintained, with br/Eip93F labelling mid-born neurons, while dan/danr labelling later born ones.
We are not sure which part of Fig. 4g the reviewer is referring to. The top part shows expression in 03A-T1, while the bottom part shows expression peaks across hemilineages. Several shTFs display multiple expression peaks and are therefore not bound to a single temporal window, but the order is maintained.

Minor points:

3.12- It is surprising that only 6 glial types are identified. In other brain regions many more numerous glial types have been identified.

We are aware that the annotation of glial types is not exhaustive. Although this study primarily focuses on neurons, glial cells are present in the dataset and we expect them to constitute a valuable resource for researchers studying glia. Accordingly, we provide broad annotations of the major known glial classes, while leaving comprehensive subtype annotation of the glial population to future studies.

3.13- When it comes to serial lineages, the presence of a dominant Hox gene is in strong support of the position along the rostro-caudal part of the axis. The authors appear concerned by the overlap between Hox genes which is not an issue due to posterior dominance.

We are not sure where in the text the reviewer perceives a "worry" for Hox genes co-expression. The main point we make about co-expression is that it makes segment identity annotation more difficult. We have now provided additional evidence in support of Hox genes expression based segment annotation (see response 1.1)

3.14- One potential caveat is that clusters might not represent pure hemilineages as neurons from hemilineage might jump clusters and become more similar to neurons from another one. The authors are careful about this and include Gal4 lines labeling specific lineages in the sequencing. However, in Fig. 2c, sections of the labeled trajectories are missing the label, which do not look random (e.g. due to GFP dropout). Depending how clean and complete those Gal4 lines are, this points to evidence that hemilineages do mix in their "trajectories". Most lineage-related neurons remain similar to each other because all seem to have a "main" cluster where most of the label is, but it is clearly not that neat.

While in the optic lobe it is commonplace that the same cell type is produced by numerous progenitors, from connectomics work we can confidently state that in other parts of the *Drosophila* nervous system this is exceptional, especially for type I neuroblasts. Kenyon Cells are a notorious case, but their development is different (mushroom body neuroblasts produce only one neuronal fate at a time, no Notch-ON, Notch-OFF hemilineages). The only other reported case we are aware of involves some dopaminergic neurons in the CB (doi: 10.7554/eLife.53518), while we did not come across any such case in secondary VNC neurons in the connectome.
We would like to point out that in Fig. 2c the majority of neurons come from non-labelled lines. Reviewer Fig. 8 shows how data from a single animal would look like in the UMAP. We think that it is more informative to focus on the absence of labelled cells decorating other trajectories than the expected one (with the exception of 10B and 09B, which are not the primary lineages labelled by the labelled lines, but for which we confirmed expression through stainings and confocal imaging).
There are multiple reasons why parts of trajectories might be missing the label, some of which are specific to the type of reagent employed (immortalisation or split-GAL4). These reasons include:
-dropouts (more or less pronounced depending on the driver and reporter, immortalisation lines have lower expression levels)
-delayed or patched expression of the driver (for split-GAL4)
-stochastic recombination, either lateralised or NM restricted producing a mix of GFP+ and GFP- cells (only for immortalisation lines)
-delayed recombination (only for immortalisation lines, notably, most of the "holes" in expression appear in the part of trajectory bearing early-born neurons. It is to be noted that while recombination should be limited to dpn+ expressing cells, GMCs may inherit the recombinase, resulting in independent recombination events that are not propagated to the rest of the lineage).

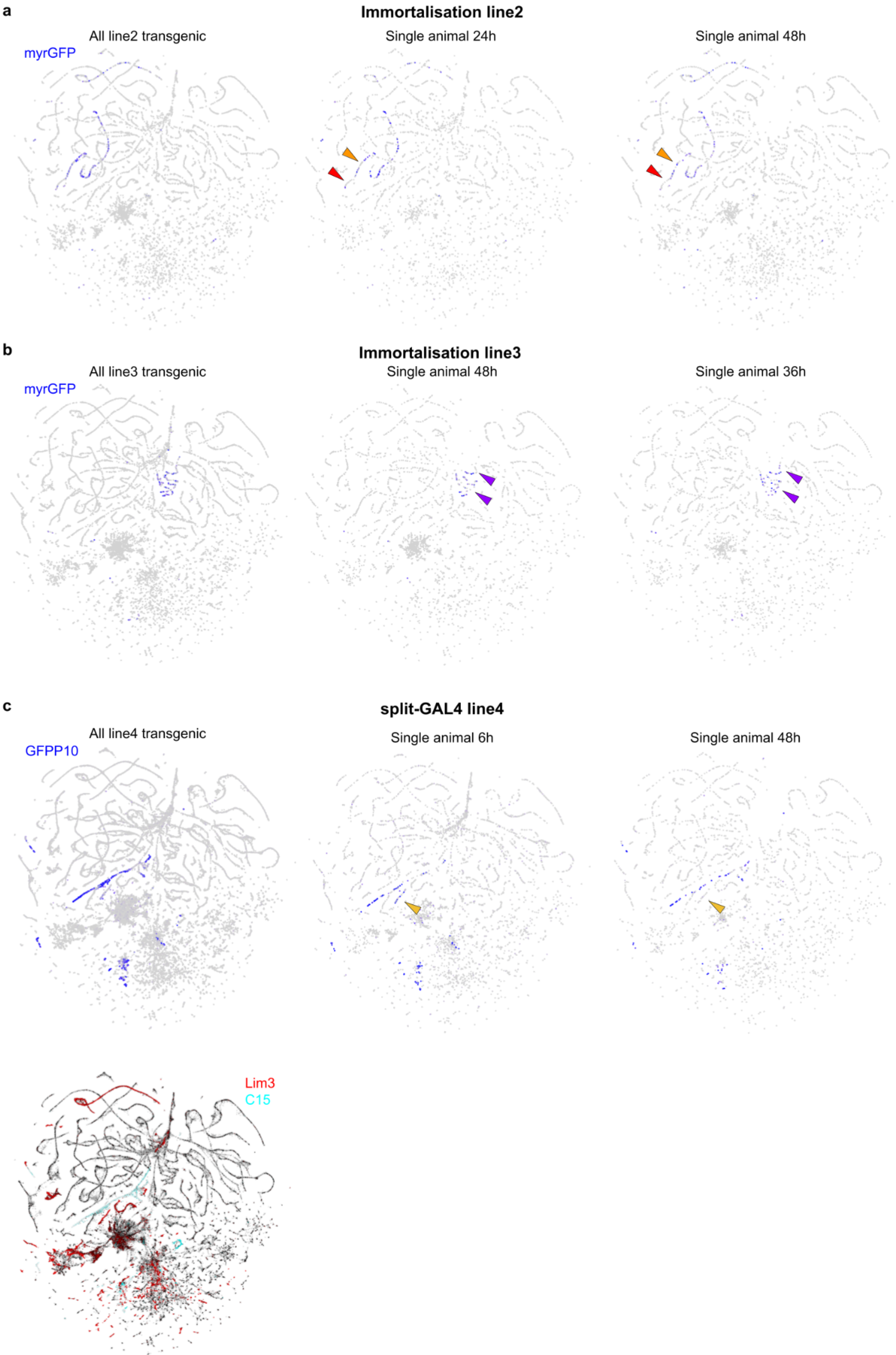
a
Immortalisation line2
All line2 transgenic
myrGFP
Single animal 24h
Single animal 48h
b
Immortalisation line3
All line3 transgenic
myrGFP
Single animal 48h
Single animal 36h
c
split-GAL4 line4
All line4 transgenic
GFPP10
Single animal 6h
Single animal 48h
Lim3
C15

Reviewer Fig. 8: Labelled lines - single animal expression. Expression of the GFP reporter only in samples of the correct genotype in two immortalisation lines (a-b) and one split-GAL4 line. Left column has cells from all the animals of the correct genotype. Middle and right column have cells from a single VNC from the indicated developmental stage. a, Red arrowheads point to trajectory location devoid of GFP expression across animals, corresponding to early-born secondary cells in the lineage. Orange arrowheads point to a location with no expression in the 24h animal, but labelled in the 48h sample. b, Purple arrowheads point to locations with no expression in the 48h sample, but labelled in the 36h sample. c, Yellow arrowheads point to 09B hemilineage trajectory, labelled in the 6h sample but not at 48h. The expression pattern of the two genes used for the intersection is shown (lim3 in red and C15 in cyan), where 09B does not show C15 expression. This gene is expressed early in development and the GFP detected at 6h is likely resulting from slow degradation rate of the transgene.

In conclusion, this is an interesting atlas of the ventral nerve cord but several of the conclusions should be strongly dimmed or eliminated since these arguments have already proven to not be correct in other, much easier systems. The authors should be much more scholarly and look beyond the nerve cord or even the central brain and discuss data from the visual or olfactory systems that are not consistent with their own arguments, simply because even current brute force is not enough to go deep enough in the transcriptome of very sparse neurons.

Referee #4 (Remarks to the Author):

"A Developmental Atlas of the Drosophila Nerve Cord Uncovers a Global Temporal Code for Neuronal Identity" by Cachero and colleagues presents a transcriptional atlas of the developing adult Drosophila nerve cord, leveraging scRNA-seq from four pupal time points. The authors provide a comprehensive annotation of neuronal populations developmental stages. They link transcriptional identities to morphological and functional characteristics, investigate sexual dimorphism, and provide contextual insights into the general principles of neural development. This study is a significant advance in the field, providing high-resolution characterization of multiple developmental features (birth time, hemilineage, birth order, sex). Uniting transcriptomic, developmental, and connectomic variation at scale offers novel insights into neurodevelopmental mechanisms. The manuscript identifies generalizable principles in neural development and circuit assembly. The authors unite single-cell transcriptomics and connectomics to address the longstanding challenge of linking molecular identity to circuit architecture across the nervous system. This resource will be valuable for both developmental and systems neuroscience.
Sequencing across developmental stages allows the authors to interrogate the role of temporal patterning, hemilineage, and segmental regionalization in neurodevelopment. Dimensional reduction (UMAP) convincingly segregates primary (embryonic) and secondary (larval) neurons in transcriptomic space. Manual annotation using established molecular markers is appropriate, and the clear segregation of neuronal clusters corroborates previous findings. Integration of lineage information from connectomics and molecular markers is an asset that facilitated automated neuronal classification. The authors identify transcription factor combinations for hemilineage classification, with nearly half identifiable by a single marker. These markers allow for classification across developmental time, overcoming annotation challenges for immature neurons. The use of Hox transcription factors to assign segment identities is convincing and supported by developmental and anatomical context. The finding that serial homology is evident in transcriptomic and connectomic data is novel and provides mechanistic insight to neuronal morphology. Pseudotime calculation for each hemilineage reveals temporal transcription factor dynamics, which are clearly presented. Identification of sustained temporal transcription factor suggests terminal selector function, an important distinction from the role of temporal transcription factors in embryonic neurogenesis. Because the temporal transcription factor code is shared with the developing brain, the findings herein likely highlight general principles of neurodevelopment.

Additionally, the authors investigated sexual dimorphism in the nerve cord, identifying differentially abundant populations which are validated by sex-determination gene expression. They find that this dimorphism is biased by neuronal birth time such that male enriched neurons are mostly secondary neurons while female enriched are mostly primaries. Transcriptional dimorphisms align with knowns from connectomic data, and their atlas can be used to confidently predict dimorphisms. The authors find that female-specific apoptosis underscores dimorphism to some extent. Furthermore, differential guidance cue receptor expression between sexes is suggested to drive morphological dimorphism, a significant contribution to linking molecular and morphological features between transcriptomes and connectomes, respectively.

Emphasis on developmental timing and predictive modeling is well supported. Methods are well-suited and appropriately chosen. Data quality and presentation are high. Statistical treatment and assessment of uncertainties are sufficient.

The conclusions are strongly supported by the methodology. The multi-level annotation, careful consideration of segmental identity genes and sex, and integration with connectomic data all substantiate the authors' claims. The manuscript is overall clearly written and provides ample methodological and scientific context. The abstract, introduction, and conclusions frame the findings well for both expert and non-expert readers.

Suggested Improvements

4.1 Clarify terminology such as “VNC hemisphere” (e.g., line 11) and check for spelling consistency (organization/organisation).

We thank the reviewer for spotting these inconsistencies and unclear terminology. We have carefully revised the use of British vs American English spelling (adhering to British English) and substituted “VNC hemisphere” with “one lateral half of the VNC connectome”.

4.2 The discussion around lines 66–69 is confusing and could be more explict.

We apologize for line numbering inconsistency due to the absence of the hundred digit. We think in this comment the reviewer is referring to the atlas not being fully aligned to the connectome. We have revised **lines 620-630** providing more details on how transcriptomic and connectomic types can be further cross-matched by exploiting shTFs expression.

4.3 Explicitly differentiate "birth time" (embryo/larva) from "birth order" within lineages in discussion (lines 87–98)—this may be confusing for readers unfamiliar with insect neurodevelopment.

We thank the reviewer for this suggestion. While we had tried to be as consistent as possible, we have realised there were sections in the manuscript where the use of similar words to refer to different concepts was prone to confusion. We have revised the text, now using the terminology “neurogenesis wave” when referring to the distinction between primary and secondary neurons and “birth order” for temporal differences within lineages in secondary neurons. We hope this solution helps clarify the context.

4.4 Add citation for lines 89–90 as appropriate.

As mentioned above, the line numbering in the manuscript appears to be inconsistent. We therefore suspect this comment refers to the prediction of neurotransmitter identity. We have now added a reference in **line 257** for neurotransmitters usage in MANC.

4.5 The claim of "38x coverage of the half-VNC" may be slightly overstated, as sequencing was performed over 4 developmental stages rather than on mature tissue.

We have revised our coverage claim, adding the word “aggregate” when referring to all developmental stages combined. We have also added a clear reference to **Extended Data Fig. 1**, where per stage coverage is reported [**line 175**]. See also response 3.6.

This work is a valuable contribution to neurodevelopmental biology and Drosophila connectomics. With minor revisions for clarity and citation, it will be suitable for publication.
